# Soy and Isoflavones: Revisiting Their Potential Links to Breast Cancer Risk

**DOI:** 10.3390/nu17162621

**Published:** 2025-08-13

**Authors:** Catherine Bennetau-Pelissero

**Affiliations:** Department Feed & Food, Bordeaux Sciences Agro, 1 Cours du Général de Gaulle, Gradignan Cedex, 33175 Bordeaux, France; catherine.bennetau@laposte.net

**Keywords:** breast cancer, soybean, isoflavones, cell culture, rodents, clinical trials, population studies, food preparation

## Abstract

Soy has a long history of consumption in Asia and was traditionally prepared by rinsing, cooking, and simmering, methods which remove estrogenic isoflavones (Isofls). Population studies have indicated that soy and/or Isofls may be associated with a decreased risk of breast cancer (BC), while in vitro and experimental data indicate dose-related proliferative effects of Isofls on breast cells. This review attempts to decipher the role of soy and Isofls in the risk of BC in women, since previous studies have suggested a lack of association with BC. Several dozen population studies conducted in Asian and Western countries were analyzed, as were data collected during in vitro animal and clinical trials of relevant doses of soy and Isofls. Although soy intake has been estimated well in Asian countries and could be related to preventive effects on BC risk, this has not been the case in the West, where the consumption of hidden soy is often omitted. However, in both cultures, the Isofl intake is misestimated, and the groups are misclassified. Indeed, in Asia, the origin of soy foods, i.e., homemade or industrial, has never been reported, and in the West, the amount of Isofls consumed in hidden soy has not been determined. Moreover, in most cohort studies, only a few subjects were exposed to active doses of Isofls on breast cells. Similarly, clinical interventions showed estrogenic effects of Isofls at relevant doses. Finally, population studies have not shown any convincing link between soy or Isofl intake and BC risk, likely because they have opposite effects on this pathology. Thus, based on in vitro, experimental, and clinical data, a deleterious effect of Isofls cannot be excluded when active doses are ingested, even if the soy food matrix can be protective.

## 1. Introduction

Breast cancer (BC) is a critical public health problem worldwide [1]. It is the second most common cancer diagnosed and a leading cause of death in women [1]. The reality of BC is complex [2,3,4], with some tumors sensitive to estrogens and expressing ERα and/or ERβ [2] and others expressing HER2, an epithelial growth factor [3], while others are classified as triple-negative BC (TNBC) [4]. TNBC comprises a variety of cancers, some of which can respond to estrogens and xenoestrogens via a transmembrane receptor—the G-Protein coupled Estrogen Receptor (GPER). The affinity of this last receptor for Isofls and several drugs used in BC management, such as tamoxifen or fulvestran, may lead to a multidrug-resistant pattern that makes treatment particularly difficult [5].

**Figure 1:**
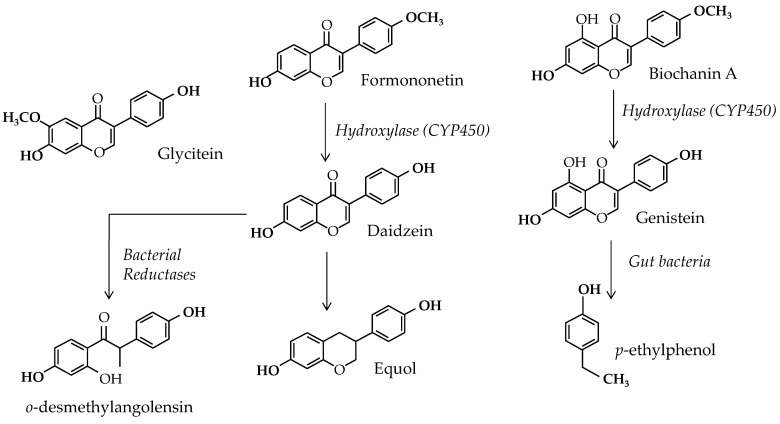
Soy isoflavones and parent compounds, with metabolic links between them.

In this context, some authors continue to seek natural preventive or curative treatments and debate the potential effect of herbal medicines or functional foods to reduce BC risk [6]. The potential role of soy and Isofls in estrogen-dependent BC is part of this debate. Soy Isofls are weak estrogenic polyphenols belonging to the flavonoid family present in legumes, where they exert several crucial effects. Indeed, they have been shown to attract symbiotic organisms: rhizobium bacteria and mycorrhizae fungi [7]. These partners form nodules on legume roots that enable atmospheric nitrogen fixation and high-level protein synthesis [8]. These Isofls are also known to act as alert substances when the plant is attacked by pests [9]. Soy contains three types of Isofls, i.e., genistein, daidzein, and glycitein, but these substances can be obtained from precursors present in several clover species and alfalfa. They can also be transformed into active—equol—and inactive—*o*-desmethylangolensin and *p*-ethylphenol—metabolites by the gut microflora. The Isofls and their parent compounds are shown in Figure 1.

The effect of estrogenic Isofls on BC is still a subject of controversy. Indeed, results obtained from biological studies show discrepancies, in which some effects are beneficial, while others are deleterious [10]. Questions remain because in human populations, Isofls are associated with soy consumption [11]. Indeed, soy was shown to contain other substances that potentially prevent BC [12] and is usually consumed in a context where other BC inductors are reduced [13]. In addition, this legume is associated with traditional diets in Asia [14] and with a healthy diet in Western countries [15]. Therefore, confounding factors could bias the effects of both the food and the nutrient, as well as the interpretation of the results.

Indeed, it was recently determined that traditional and domestic Asian cooking methods can reduce Isofls in soy foods [16] thanks to basic water treatments. This reduction mainly occurs when soy is prepared at home by following family recipes. Population studies in Asian countries may miss this context when attempting to show the effect of soy and Isofls on health issues. In fact, in these studies, the estimation of exposure to Isofls is usually calculated from Isofl concentrations measured in industrially prepared soy foods obtained in marketplaces [17]. Worse, in some cases, the database used lacks values obtained from local soy foods [18]. At any rate, in these circumstances, the commercial soy foods were prepared at a large scale, with reduced water treatments. Consequently, these commercially available soy foods are most likely to contain higher amounts of Isofls than homemade soy foods. Since the food frequency questionnaires used in population studies usually do not assess the origin of the soy foods eaten by the participants, the estimated Isofl intake may be biased.

To resolve this problem, this review examines available relevant data on the roles of soy and Isofls in BC. The aim is to clarify the effects of soy and Isofls on BC risk.

## 2. Isoflavones and Breast Cancer In Vitro

### 2.1. Different Types of Breast Cancers

In vitro cell models are simplified systems that cannot always be superimposed on the in vivo situation. Many different types of BC can only be identified via specific target cells, i.e., canal, lobular, basal, and parenchymal cells [19]. Some are invasive and induce metastasis. These cancers are also characterized by many different receptor expressions. Among the crucial receptors presently considered in clinical approaches are the canonical estradiol receptors (ERs), from which tumors are then identified as being either ER+ or ER−. However, ER+ tumors can express various proportions of the two estradiol receptor forms: ERα and ERβ. While such a detailed description is not always assessed, it could be crucial since xenobiotics may have different affinities for these two receptors [20]. Indeed, Isofls have a higher affinity for ERβ. Additionally, these forms can be present at the nucleus in an active form, in the cytoplasm as a reserve form, or below the cell membrane as forms involved in rapid cell signaling [21]. Several isoforms of both ERs obtained by alternative splicing during transcriptional steps also exist. They can induce different reactions to xenobiotics depending on their resulting ligand binding abilities and the conformation of their activation functions [22]. The detection of PRs (both PR-A and PR-B) is also a clinical characteristic of breast tumors, since PR synthesis is under the control of an ER−-dependent transcriptional activity [23]. Therefore, a breast tumor can be identified as PR+ or PR− based on its ability to express these receptors. Note that in normal cells, the proportions of PR-A and PR-B are usually equal and that a significant imbalance is usually observed in tumor cells [24]. In addition, HER2 is also considered in clinical classification, as it is a growth factor receptor that worsens the prognosis of BC [25]. Again, clinical classification considers tumors that can be either HER2+ or HER2−. Finally, another estrogen receptor has been found in almost all BC cells, including in TNBC. It is called GPER and is a transmembrane estrogen receptor that is expressed in specific conditions in vitro [26]. For the moment, GPER is not clinically diagnosed, although it may be involved in several resistance pattern to various drugs [27]. Furthermore, in vitro, there seems to be two distinct pathways activated via GPER. At low doses, estradiol (E2) and other estrogens stimulate the EGFR-dependent pathway, inducing cell growth, but at high doses, ≥1 µM, they induce a cAMP-dependent pathway preferentially involved in cell apoptosis. The ligand-binding domain of GPER is large and can be filled with two molecules. It is likely that the GPER conformation differs depending on whether one or two ligands are bound to the binding domain. This may explain why the response via GPER is different according to the availability of the ligands. Isofls from soy have been shown to bind to ERα, ERβ [28], and GPER [29] at doses that can be achieved in vivo.

### 2.2. Methodological Limits of In Vitro Studies

#### 2.2.1. Doses Tested

In vitro, cells are in survival conditions. They usually lack the main cell interactions that exist in vivo and are maintained in simple media that often lack the basic factors existing in vivo. More important, they are often submitted to doses of hormones, drugs, or xenobiotics that are very different from those existing in vivo. Indeed, if E2 is usually tested in the pM range, which is the normal range of concentrations in pre-menopausal women, other substances can be tested at pharmacological concentrations that are much higher than those occurring in vivo. This is often the case for Isofls. Indeed, several studies on the pharmacokinetics of Isofls [30,31] or the disposition of these compounds in breast tissue [32,33] showed that their concentrations are in the nM range and, at the very best, around 1 µM. These doses correspond to the sum of conjugated and aglycone substances, and the aglycones only represent 1/10 of the total Isofls [31]. At these doses, their effects can be either estrogenic or null. However, many studies looking for protective effects of Isofls on BC were and are still performed at doses from 10 to 500 µM [34,35]. Indeed, the scientists who used these doses probably assumed that the higher the dose, the more pronounced the effect. However, this assumption is wrong. Looking closer at the cell machinery, the signalization pathway has been found to depend on the dose of endocrine disruptor used [35]. Indeed, genistein from soy is known to be an inhibitor of tyrosine kinase, with an IC_50_ at 20 µM [36]. It was sold by chemical providers as a specific inhibitor of tyrosine kinase until the end of the 1990s. Since many intracellular pathways involve tyrosine kinase activity, it is not surprising to find that genistein at doses over 10 µM inhibits cell growth, and this effect has nothing to do with an estrogenic or anti-estrogenic effect. In this review, we will only consider works performed with physiologically relevant doses, and thus, always <5 µM.

#### 2.2.2. Forms and Cocktails

Some studies have dealt with the estrogenic potency of soy or other legume extracts directly tested in vitro. The system used was either a yeast system transfected with basic estrogen receptor machinery [37] or a cell model. In some cases, several enzymatic reactions were preserved, such as in hepatocyte cultures [38], but in others, the cells were models for breast tissues [39]. Although such tests are used in pharmacology as a first screening step to check for the presence of active compounds in herbal preparations, they have numerous limits, one of which is linked to plant physiology. Indeed, in plants, the active compounds may not be in metabolic forms equivalent to those found in human consumers. Hence, in soy matter, Isofls are essentially in conjugated forms, i.e., glucosides, acetyl, or malonyl forms, which are all soluble in water. However, an extract is only a part of a plant, and its composition essentially depends on the solvent used for the extraction. If this solvent is organic and not edible, there is a risk that the extract will not reproduce what is absorbed during oral intake. Additionally, during the ingestion process, the gut and the liver sort the different substances of a mixture. As a result, the substances present in the blood are not in the same forms nor in the same proportions as in the plant. If there are different substances in an extract and if these substances do not have the same effect in a basic test, then their results could not be used to explain the in vivo situation in humans. Finally, extracts can be tested at pharmacological concentrations that are far from being reproducible in vivo. Therefore, in vitro tests performed with plant extracts have only indicative values and cannot be extrapolated to the in vivo situation.

As already mentioned, Isofls such as E2 are mainly present in the blood and lymph as well as breast tissue in conjugated forms. These are glucurono- and/or sulfo-conjugated Isofls that are physicochemically unable to enter a cell. This explains why the distribution volume of Isofls is low in animals and humans [40]. It is thought that some cell types, including hepatocytes, can free the aglycone forms in vitro [41], but this remains disputed for other cell lines, including breast cells. Indeed, the ability of a cell line to free xenoestrogens from their conjugating residues would be crucial to be considered in cell specificity approaches [42].

Additionally, after soy intake, many substances can reach a breast cell. This includes the three main Isofls from soy, i.e., genistein, daidzein, and glycitein, as well as the metabolites equol and *o*-desmethylangolensin. All these substances are of course under aglycone and mainly under conjugated forms. Therefore, before considering the effect of Isofls on BC cells in vitro, it should be remembered that they are cocktails of substances that reach the cells in vivo and that these cocktails can also include E2 and its other estrogenic metabolites, i.e., estrone (E1), estriol (E3), or estetrol (E4). Finally, eating soy food also results in the digestion of other substances such as saponins and sapogenins, carbohydrates, fibers, peptides, enzymes, complex polyphenols like tannins, phytic acids, minerals, and vitamins that can induce complex responses from the gut and interfere directly or via secondary messengers with target cell functioning.

### 2.3. Summary of Isoflavone Effects In Vitro on Breast Cancer Cells

The in vitro approach is the most convenient in many cases since it is easily affordable to many scientific teams. Thus, many data are available nowadays on these models, and in many cases, these studies were performed using high doses of Isofls and showed pro-apoptotic effects on BC cells. These studies were designed to explain the seemingly protective effect of soy and Isofl consumption in Asian populations on BC [43]. Although this protective effect will be analyzed later on in this review, scientists have looked for substantial arguments to explain how estrogenic substances could prevent estrogen-dependent diseases. The first approach dealt with a competitive process between the native E2 and the xenoestrogens from soy. In vitro, specific proportions of both E2 and Isofls were found, and they showed an inhibiting effect of the weak plant estrogens on the native E2 [44]. However, this phenomenon occurred when the proportions of E2 and Isofls reached a perfect balance, which depended on the relative affinities of the two types of substances with the ERs. Such a proportion could be maintained in vitro, but in vivo, both Isofl and E2 concentrations fluctuate with time [45,46]. Therefore, the relevant balance between the two types of compounds can only be achieved transiently. In addition, the mechanisms that were involved in BC prevention by high doses of Isofl were numerous. They dealt with antioxidant properties, anti-tyrosine and anti-protein kinase effects, Bcl-2 inhibition, Akt signaling inhibition, anti-angiogenic effects, anti-DNA topoisomerase effects, and NF-κB downregulation [47,48]. More recently, epigenetic footprints were observed at doses of genistein > 50 µM and daidzein > 100 µM [49]. All these effects cannot be achieved in vivo since Isofl concentrations, by the target cells, only reach 2 µM in exceptional conditions. Thus, we will not focus on them. Table 1 below shows the effect of soy Isofls in vitro when tested at physiologically relevant concentrations.

Table 1 mainly shows that Isofls in aglycone forms and at physiological doses can induce cell growth or have no effect on cell proliferation. This depends on the availability of estrogen receptors. At doses achievable dietarily, they do not induce cell apoptosis or reduce BC cell proliferation.

## 3. Soy or Isoflavones and Breast Cancers in Animals

### 3.1. Toxicological Studies

As mentioned previously, soy Isofls undergo complex metabolism after ingestion, and this affects their forms and concentrations in blood, lymph, and cell environments. Thus, it could be helpful to develop BC cell cultures stimulated by plasma or lymph basic extracts [56]. However, to more accurately represent the human in vivo situation, tests can also be developed in animal models. In these cases, it is crucial to respect oral exposure to Isofls, since other routes can shunt metabolic reactions and deeply impair models’ metabolome [57]. The main limit of animal models is that they have their own metabolism. Indeed, rats, mice, monkeys, and pigs have different gut and liver enzymes, and this affects the resulting composition of the biological fluids that reach mammary cells in vivo [58]. Considering soy Isofls, one of the main differences between animal models and human beings is the ability of their gut flora to produce equol [5]. Indeed, rodents and monkeys are all equol producers, while only a portion of humans and pigs harbor the competent bacteria [58,59]. Moreover, the ability of liver enzymes to produce glucurono- and sulfo-conjugates of Isofls is not the same in rodents, non-human primates, pigs, or humans [58,59]. Although the conjugates are all considered to be ineffective, the proportion of aglycone forms directly impact the estrogenic potency of the Isofl in the different models [58,59]. Nevertheless, after an oral challenge, the microenvironment of BC cells is closer to that of human consumers than that in in vitro cell culture media.

#### 3.1.1. Official Toxicological Studies

In 2008, the National Toxicology Program (NTP) of the USA published a comprehensive multigenerational, 2-year dietary study on the potential carcinogenic effect of genistein, one of the main forms of dietary soy Isofls [60]. The study was performed on Sprague Dawley rats and started during the gestation of dams. The dietary exposure concentrations were 0, 5, 100, and 500 ppm and led to serum genistein levels possibly occurring in human consumers. The ingestion rate was calculated for the animals at different periods of life. It was found to be 0, 0.5, 9, or 45 mg/kg body weight per day during female pregnancy and 0, 0.7, 15, or 75 mg/kg bw/day during lactation. A minimal transfer of genistein to pups was observed via the dams’ milk and after weaning; the exposure of animals prior to postnatal day (PND) 140 was approximately 0.4, 8, or 44 mg/kg bw/day for females and 0.4, 7, or 37 mg/kg bw/day for males. For the period between PND140 and the end of the study, mean ingested doses were approximately 0.3, 5, or 29 mg/kg bw/day for females and 0.2, 4, or 20 mg/kg bw/day for males. The study included three exposure arms:

1. Continuous exposure from conception through the 2 years, designated F(1)C;
2. Exposure from conception through PND 140 (20 weeks), followed by a control diet until 2 years, designated F(1)T140;
3. Exposure from conception through weaning (PND 21), followed by a control diet until 2 years, designated F(3)T21. The animals of this group were a third generation of animals exposed to genistein from F0 pregnancy and issued from the multigenerational reproductive toxicology study of NTP USA [61].

For the study, 50 animals per sex were assigned to each exposure group in each arm of the study. Animals of the control groups that were moribund or that died prematurely were sampled for histopathology, and the observations were included in the study. The study showed that the survival was similar in all groups, ranging from 62 to 86% for males and 43 to 64% for females.

The mean body weights of 500 ppm F(1)C females and F(1)T140 animals were always lower than that of the control group. Females of the F(1)C, F(1)T140, and F(3)T21 groups fed 500 ppm genistein all showed the early onset of aberrant estrous cycles, suggesting early reproductive senescence. These phenomena were also observed in the F(3)T21 females at 5 and 100 ppm genistein. Pituitary gland weights were significantly increased in females of the F(1)C and F(1)T140 groups fed 500 ppm genistein, as was also the case in the females of the F(3)T21 group fed 100 ppm genistein. In F(1)C females, there was a significant positive trend in the incidences of mammary gland adenoma or adenocarcinoma with genistein doses. Moreover, the incidence of mammary gland adenoma or adenocarcinoma was significantly greater in the 500 ppm genistein-fed group than in the controls. Parallelly, the incidence of benign mammary fibroadenoma tended to decline with genistein doses in F(1)C females, and it was significantly reduced in the 500 ppm group compared with the controls. In F(1)T140 females fed 5 and 100 ppm genistein, the combined incidences of adenoma and adenocarcinoma tended to be reduced compared with the control or to the groups fed 500 ppm genistein. A positive trend was observed in the incidences of mammary adenoma or adenocarcinoma in F(3)T21 females. There were positive trends in the incidences of adenoma or carcinoma in the pars distalis of the pituitary gland of females in the F(1)C and F(1)T140 arms. The pars distalis contains the gonadotropic cells controlling reproductive cycles. Moreover, in the F(1)C group, the incidence of these adenomas or carcinomas of the pituitary gland was greater in the 500 ppm group than in the controls.

In F(1)C males, the incidences of combined adenoma or carcinoma of the pancreatic islets significantly increased dose-dependently, but this incidence was not significant in the 500 ppm group compared with the control group. Additionally, there was no evidence of a carcinogenic effect of 5, 100, or 500 ppm genistein in male Sprague Dawley rats exposed continuously for 2 years, from in utero life until PND140, or for the third generation from in utero life to weaning.

However, in female Sprague Dawley rats continuously exposed to genistein for 2 years, there was some evidence of carcinogenic activity of genistein based on an increased incidence of mammary adenoma or adenocarcinoma and pituitary neoplasms. In parallel, in these females, there was a significantly reduced incidence of benign mammary gland fibroadenoma when female rats were fed 500 ppm genistein. In female Sprague Dawley rats exposed to genistein from conception through to PND140 and then to a control diet until euthanasia, there was equivocal evidence of carcinogenic effect based on increased incidences of pituitary gland neoplasms. In female F(3)T21 offspring, issued from three prior generations of animals treated with genistein and exposed from conception through weaning to Isofls before shifting for 2 years to a control diet, there was equivocal evidence of carcinogenic activity of genistein based on increased incidences of mammary adenoma or adenocarcinoma. Exposure to genistein also accelerated the onset of aberrant estrous cycles in all female Sprague Dawley rats fed 500 ppm genistein.

The effects of genistein on estrous cycling and the incidences of common hormonally related spontaneous neoplasms of female Sprague Dawley rats were consistent with an estrogenic mechanism of toxicity. The study, which followed the OECD procedures, clearly indicated that genistein and its blood metabolites could induce mammary tumors after long-term continuous exposure to dietary achievable doses of genistein. Such an effect is carcinogenic and not only growth-promoting. As a result, considering the reliability of this toxicity evaluation, it can be said that genistein may not be safe for the mammary gland. This is true for continuous exposure to a diet containing significant amounts of genistein.

#### 3.1.2. Studies Involving Tumorigenic Substances

The previous paragraph illustrates the ability of genistein to induce carcinoma and adenocarcinoma de novo. However, based on population studies, the question may also be to determine if Isofls from soy can or cannot prevent the tumorization process from normal cells to tumor cells in vivo. To answer this question, experimental carcinomas were induced and the effects of Isofl or soy extracts on this process were analyzed. In these cases, the recorded effects concerned the ability of the Isofl to prevent or accelerate the early cancerous process, while a proliferative effect would only indicate a growing effect on already-tumoral cells. Experiments have been performed by numerous scientists to date involving either purified Isofls, extracts enriched in Isofls, or soy proteins and even soy foods such as soy milk. The previous authors tended to answer several different questions. In some cases, they tried to determine a preventive effect, and soy or Isofl treatments were performed or started before the chemically induced cancerization. On the contrary, they could also try to determine if soy or Isofls could inhibit tumorization once the process was already induced. The following tables gather data already published so far. To try to homogenize these data with those of the USA NTP, the doses tested are expressed in mg/kg bw/day, and the plasma levels were estimated as accurately as possible. Table 2 gathers studies where soy or Isofls were administrated prior to carcinogenic treatment. In all cases, only studies dealing with oral exposure were considered to integrate the metabolic reactions occurring in the digestive tract.

From Table 2, it seems that low doses of genistein (1 to 25 mg/kg bw/day), given orally to dams’ nursing pups, would protect these pups during their adult life, even if according to [60], the genistein amounts transferred to pups was very low. This was seen via a lower incidence of mammary tumors in pups exposed in utero and neonatally until weaning. At higher doses (28 to 40 mg/kg bw/day), no effects were recorded. Additionally, the inhibition seemed significant only if the animals were followed for more than 20 weeks. After weaning but prior to DMBA, genistein had no effect or seemed to activate tumorization. Equol was not efficient when given orally and neonatally and until PND35. The effects of soy protein or a soy diet were not so clear. According to [65], gestational and neonatal exposure to low doses of genistein may induce early differentiation of the mammary tissue, which played a role against the tumorization process induced by DMBA. However, these experiments did not exclude a potential effect of Isofls on the bioavailability of DMBA nor an epigenetic effect on mammary cells preventing the synthesis of an oncogene such as MDM2, which is produced under DMBA treatment [77,78].

Table 3 gathers data obtained when soy or Isofls were administrated after the carcinogen.

Again, Table 3 only gathers data obtained on rats fed known amounts of Isofls. One study by Gotoh et al. [85] reported a preventive effect of soy protein; miso; and biochanin A, a metabolic precursor of genistein, on MNU-induced mammary tumors, but the amount of Isofls could not be determined. Thus, the study does not appear in Table 3. Looking at the results presented in this table globally, the effects of Isofls or soy only administrated after the cancerogenic substances were not so clear. Hence, the study from Lamartiniere [65], which included a first exposure before the DMBA treatment, showed an inhibitory effect. Similarly, the study by Liu et al. [82] showed a slight inhibitory effect with Isofls delivered after DMBA, but only for a 3-week duration. Equally, a study from Ma et al. [83] conducted with high doses of a soy extract showed an inhibitory effect. In that case, at least two hypotheses could be made to explain these results: (1) the effects were linked to the doses used, and (2) the soy extract contained other substances that could be involved in the tumors’ regression [86]. However, the precise composition of the soy extract was not disclosed in the study, and therefore, it was difficult to determine what could be responsible for the inhibitory effect recorded.

Finally, it appears that only neonatal exposure to relatively low levels of genistein, i.e., those present in dams’ milk, would be able to counter the tumorization process induced by the cancerogenic compound DMBA [65]. Such an effect is observed later in a rat’s life and may involve the epigenetic regulation of either oncogenic or anti-oncogenic genes [87]. According to [88,89], a first explanation would be that early exposure to lactational genistein would enhance the maturation of the mammary gland in female pups, and this maturation process, dealing with mammary cell differentiation, would counteract the de-differentiation process involved in cell tumorization. Because the development stages of rats are different from those of humans, it is difficult to determine the equivalent period in humans’ lives. Indeed, rat exposure did not mimic the one produced by soy-based infant formula [61].

### 3.2. Humanized Rodent Models

Most studies performed on humanized rodent models were performed on athymic nude mice implanted with human BC cell lines. Such tests allow the human cells to be in contact with circulating forms of Isofls, although some differences can exist between the human and rodent situations in terms of concentrations, proportions of conjugates and aglycones, and proportions of sulfates and glucuronides [59]. At the very least, models can show that these circulating forms could be active. Provided that the tests were performed at dosages inducing relevant concentrations for human beings, they can be informative. Indeed, Isofl plasma levels were recently assayed in French women, including a subject consuming soy products several times a day [90]. The median and max levels of Isofl in these plasmas were 0.135 μM for genistein and 0.097 μM for daidzein in the general population and 1.73 μM of genistein and 0.99 μM of daidzein in the high-soy consumer. This means that a 2.62 µM concentration of total Isofls (including conjugated phytoestrogens) in plasma is achievable in the blood of soy-eating women. However, it is not possible to strictly derive a dose effect from the rodent model to human beings because the animal is immunodeficient. Indeed, it is well known that the immune system is crucial in the management of cancer cells [91]. Therefore, the model indicates a way of effect progression but not an efficient dose. Despite all these limits, it seemed important to check for the responses of such humanized models in the following table. Hence, Table 4 gathers only data obtained following oral route administration on mice models fed either soy extracts or Isofls, where doses were homogenized for comparisons, and on estrogen-dependent cancer cell lines only.

Table 4 clearly shows that genistein and possibly daidzein in circulating forms could enhance the proliferation of human estrogen-dependent BC cells of the MCF-7 type at plasma doses ≥ 0.7 µM for genistein and ≥0.9 µM for daidzein [92,93,94,95,99,100,103,105,107]. The plasma levels, when available, help in comparing experiments with each other but cannot really help in defining an active dose for human beings. The results also indicated a negative interaction of two drugs classically used to treat estrogen-dependent BC in women, i.e., tamoxifen (TAM), an anti-estrogen, and letrozole, an anti-aromatase [55,96,102]. The effects were not observed for all athymic mice strains. The proliferation effect seemed to require a minimum duration of treatment, i.e., 15 weeks. When the duration was shorter, there was either no effect recorded or an inhibition of proliferation [101,106]. Some studies orally delivered pure compounds, either aglycones or in conjugated forms, but others tested more complex soy matrices containing small amounts of glycitein, a pure ERβ agonist, saponins, or peptides such as lunasin. When using soy matrices, the results were less clear and typically depended on the dosage of genistein and daidzein [97,98,103,105]. This did not exclude the action of other soy components, since the action of soy may result from an interaction of different molecules in an optimal proportion. Although in vitro the daidzein metabolite equol can induce cell proliferation, such a result was not observed on the athymic mice model [107]. Equol potentiated the action of genistein but had no effect per se. However, mice are universal equol producers; thus, when daidzein and soy matrices were tested, equol and its conjugates were present in the animals’ blood and in the cell vicinity.

Besides these tests involving estrogen-responsive BC cells, some experiments were performed on estrogen-non-responsive cells and, essentially, TNBC. A compilation of the main results is presented in Table 5.

Table 5 clearly shows that Isofls from soybean did not stimulate TNBC cells in athymic mice models. This effect may be in conflict with the activation phenomenon sometimes recorded in vitro with Isofls on TNBC. These were obtained via the EGFR-dependent pathway activated by GPER [51]. However, this stimulation pathway seems only to occur at low Isofl doses and GPER expression may not always occur at a sufficient level in implanted cells to exhibit any significant effect. Hence, for the moment, in vivo studies involving athymic mice and dealing with GPER pathways are still scarce [112]. They have been performed on mice implanted with SKBR3 cells.

### 3.3. Other Animal Models

#### 3.3.1. Sow, Gilts, and Piglets

As mentioned earlier, rodents may not always be considered the best models for the human transposition of physiological effects. They may differ from humans essentially in terms of metabolic issues that play a role in xenobiotics’ bioavailability and actions. Regarding equol production, as well as conjugation reactions, pigs may be considered closer to humans than rodents. Unfortunately, there is little data on the effects of soy and/or Isofls on the mammary gland in this species. According to Ford et al. [113], genistein at doses ranging from 50 to 400 mg/day in i.m. injections, exerted an estrogenic effect on gilts, as observed on tissue modifications of the utero-vaginal tract. In [114], it was shown that these i.m. treatments led to genistein plasma concentrations below 1.4 µM. Such a dose was in the range of those tested in rodent models and plausibly occurring in the plasma of human soy consumers. Additionally, the oral treatment of gilts with 2.3 g genistein/day for 93 days significantly induced mammary parenchymal cell hyperplasia [115]. In the study, genistein plasma levels were about 0.63 µM, while daidzein plasma levels were about 0.33 µM. Finally, the feeding of female neonate piglets with soy-based infant formula containing high amounts of Isofls induced the expression of mammary genes involved in cell proliferation [116]. The formula led to genistein plasma levels close to 2 µM and daidzein plasma levels close to 1.3 µM. These plasma concentrations are in the same order of magnitude to those recorded in human infants fed soy-based infant formula [117]. The genes that were significantly upregulated in gilts’ mammary gland were insulin-like growth factor 1 (IGF1), fibroblast growth factor 10 (FGF10), and fibroblast growth factor 18 (FGF18). Additionally, in [116], soy-based infant formula was shown to increase mammary terminal end bud (TEB) numbers in a neonatal piglet model fed during the postnatal period. A few months later, the same team showed that the proliferative effect of soy formulas on mammary tissues in female piglets could be related to the inhibition of some miRNAs that in turn increased the expression of some genes involved in the cell proliferation process. More precisely, soy formulas reduced the expressions of miRNA-1, -128, -133a, -193b, -206, and -27a, which increased the mRNA expressions of the genes Ccnd1, Tgfb3, Igf1r, and Tbx3. These data were consistent with enhanced cell proliferation and the suppression of the apoptotic processes in the developing mammary gland and confirm those of Lamartiniere [65] in rats. Finally, data collected on pig models showed that dietary doses of genistein and/or soy Isofls exhibited a proliferative effect on neonatal piglets and on gilts’ mammary gland and reproductive tracts. However, these effects were only partially estrogenic when compared with those induced by oral E2.

#### 3.3.2. Non-Human Primates

Monkeys were sometimes used as models to decipher the effects of soy or Isofls on reproductive tissues. As mentioned previously, monkeys are universal equol producers and, as such, the results from studies performed on monkeys cannot be translated to human beings. In addition, they seem to be poorly sensitive to Isofls, as indicated by two studies published in 2006 [118,119]. One was performed on ovariectomized female monkeys as models of menopausal women, while the other was performed on pre-menopausal female cynomolgus monkeys. In [118], high doses of Isofls (509 mg/day of genistein + daidzein) and racemic equol (1020 mg/day) were tested on the mammary gland and uterine tissues of ovariectomized cynomolgus macaques. Despite high plasma concentrations of Isofls (2.5 µM) and equol (6.9 µM), no significant effects were recorded on the observed organs. In [119], no effect was recorded on pre-menopausal female monkey with an Isofl daily intake equivalent to 120 mg/day for women. This confirms that the female monkey is much less sensitive than women since, as will be seen later, effects have been recorded in pre-menopausal women’s breast tissue with only 50 mg Isofls/day and on reproductive issues with 45 to 50 mg/day [10]. Nevertheless, the effects of such doses in menopausal women are not so well established.

In addition, a study was published in 1998 on the effect of a soy protein isolate (SPI) containing proteins and soy Isofls [120]. It reported results obtained only on five female macaques (*Macaca fascicularis*). The study compared the effects of E2, SPI alone, and SPI + E2. SPI alone was not estrogenic on the uterus or mammary glands. The amount of Isofls in the SPI was unknown, and thus, the validity of transposition of the study to humans remains in doubt. Nevertheless, although great variability was observed in the criteria studied, E2 + SPI tended to show a lower estrogenic effect than E2 alone. This was observed for mammary epithelial hyperplasia grade, mammary gland thickness, and cell proliferation in mammary gland tissues. However, for mammary gland area, the effect of E2 + SPI seemed to be additive.

To confirm the low sensitivity of monkey models to Isofls, the study by Woods et al. [121] did not show any effect of equol on the mammary gland of post-menopausal macaques. Equol was given at a dose equivalent to 120 mg/day for a human. Similarly, Schwen et al. [122] showed no effect of high dosages of equol on the uterine tract of cynomolgus monkeys.

### 3.4. Summary of Animal Experiments

All animal experiments analyzed here involved oral exposure and therefore induced BC cell contact with a cocktail of aglycone and conjugated molecules derived from soy and Isofls. These results suggest that Isofls delivered via an oral route can also be active in human beings. To summarize the animal approaches, toxicological experiments demonstrated that genistein was carcinotoxic for the mammary and pituitary glands of Sprague Dawley rats at doses that can be relevant for humans. In mice with chemically induced mammary tumors whose estrogen receptor statuses were not always characterized, Isofls inhibited the tumor growth when they were delivered at low doses in utero and before weaning. The effect of soy food seemed to be more conclusive than that of isolated Isofls. When soy or isolated Isofls were given after the cancerogenic substance, the results were less clear. The preventive effects of soy and/or Isofls on chemically induced tumors may be due to an early differentiating effect and an epigenetic effect or effect on the bioavailability and/or metabolism of the cancerogenic substances that counteracted the tumor proliferation. In mice implanted with MCF-7 cells (ER+, PR+, HER2−, and GPER+), Isofls enhanced the BC cell proliferation. The results were less clear with soy, and in several cases, the soy matrix prevented BC cell growth. In SCID mice, the results were different from those obtained on Balb/c mice. In mice implanted with TNBC cells potentially expressing GPER, physiological doses had no effects, while high doses tended to inhibit cell growth, as observed in vitro. The effect may be due to an interaction with GPER. At high doses, Isofls stimulated the cAMP pathway, inducing BC cell apoptosis. Finally, the results obtained in rodent models sustain the idea that Isofls may be protective of BC in the early stage of tumor development but enhance tumor growth when it is present. This is in accordance with the data published by Moller et al. [123]. In pigs, the effect of soy and Isofls at dietary levels was partially estrogenic or affected the mammary gland and the reproductive tract. This mimicked the effects recorded in humans with doses of 45 to 55 mg/day [10]. Finally, non-human primates did not seem to be adequate models for humans because of a poor sensitivity to Isofls.

## 4. Soy or Isoflavones and Breast Cancers in Clinical Trials

Soy and Isofls were tested on the mammary gland of women, and it sounds sensible to examine their actions on pre- and post-menopausal women separately. To understand why, we must recall what occurs at menopause and what could be the consequences at the breast tissue level. Indeed, menopause is ovarian function arrest. Ovaries are the main sources of cycling secretion of E2 and progesterone (P). During fertile life, these hormones are mainly secreted sequentially, but both of them are involved in breast tissue proliferation, differentiation, and maintenance. To summarize, estrogens induce the development of ductal tissue, P helps with ductal branching and lobulo-alveolar development, and during pregnancy and lactation, prolactin regulates milk protein production. When sexual life starts at puberty, E2 and P levels increase in the blood to initiate breast development [124]. Hence, the role of E2 is to induce cell proliferation in both the mammary glands and the uterus, while P, associated with its two PRs, seems to be involved in differentiation processes. These proliferative effects are mediated by ERs and can also concern cancerous breast and uterine cells as long as they express the ERs or GPER. As will be seen in the following paragraphs, the functional availability of ERs and PRs seems to be related to the availability of E2 and its metabolites [125,126], and E2 associated with its ERs induces the synthesis of PRs [127]. In the peri-menopausal stage, the ovarian hormones are still synthesized but in a chaotic way. Thus, the cycles tend to be irregular until they completely disappear. During that period, the ERs and PRs are still present, although the levels of PRs start to decline. Post-menopause, the levels of ERs rise in normal tissue, and it is hypothesized that this increases the tissue sensitivity to the remaining E2. During that period, a supplementation of xenoestrogen tends to attenuate the variations in estrogen plasma levels and to induce the synthesis of the PRs. The large variations in blood E2 occurring peri-menopause can induce hot flushes or night sweats, for instance. Later, when the peri-menopause phase ends, the ovarian hormones are no longer synthesized. Small quantities of E2 and P are essentially derived from adrenal gland synthesis. E2 is also obtained from the aromatization of androgens in the adipose tissue and from brain local synthesis. In this context of E2 deficiency, the functional availability of ER-β and PRs tends to decrease [126]. ERβ is known to have a greater affinity for Isofls than ERα and to counteract its proliferative action. According to [128], the decrease in E2 at menopause directly affects the immune response of women, reducing it, and this increases the risk of tumor development. However, other hypotheses can be drawn to explain the importance of timing in the effect of estrogen supplementation in menopausal women [129,130]. For instance, epigenetic modifications of ER promoters can silence the ERs in specific tissues and not in others [131]. Whatever the mechanism invoked, they explain why the reactions of pre-, peri-, and post-menopausal women to estrogens can be different and should be considered separately.

### 4.1. Clinical Effect of Isoflavones on Breast of Pre-Menopausal Women

Several studies have been conducted on the effect of Isofls on breast tissue of pre-menopausal women. In such cases, estradiol production is still present, and a synergy is potentially possible between Isofls and the endogenous E2.

Chronologically, the first study reporting the effects of soy Isofls on the breast of pre-menopausal women was that of Petrakis et al. [132], which evaluated the influence of the long-term ingestion of a commercial soy protein isolate on breast biology. This was assessed through the examination of nipple aspirate fluid (NAF). The study involved 24 non-Asian women who underwent nipple aspiration of breast fluid and gave blood and 24 h urine samples every month for 1 year. No soy was authorized in months 1–3 and 10–12. Between months 4 and 9 the women had to consume 38 g of the soy protein isolate containing 38 mg of genistein daily. The biological markers investigated were NAF volume, gross cystic disease fluid protein (GCDFP-15) concentration, and NAF cytology. Moreover, plasma concentrations of E2, progesterone, sex hormone binding globulin, prolactin, cholesterol, high-density lipoprotein cholesterol, and triglycerides were measured. Compliance was assessed via genistein and daidzein measurements in urine samples. In pre-menopausal women, there was a 2–6-fold increase in NAF volume during soy exposure compared with the period without soy. No changes were recorded in the biochemical markers followed except for E2. This hormone was erratically elevated throughout a “composite” menstrual cycle which occurred during the months of soy consumption. Still, during this period, a moderate decrease in the mean concentration of GCDFP-15 was observed. Additionally, epithelial hyperplasia was cytologically detected in 7 of the 24 women (29.2%) when they were consuming the soy protein isolate. This pilot study indicated that chronic daily consumption of a soy protein isolate containing 38 mg genistein had an estrogenic effect on the pre-menopausal female breast. In the study, daidzein was not monitored, but because daidzein usually reaches a dose that is 1/3 of the total Isofls in soybean, it could be hypothesized that the Isofl exposure was about 57 mg/day in aglycone equivalent.

Two years later, Mc Michael-Phillips et al. [133] examined the effect of a soy supplement containing 45 mg of genistein and daidzein in aglycone equivalent on the dynamic of breast growth. The proliferation rate and the expression of PRs of histologically normal breast epithelium was examine in 48 pre-menopausal women. The subjects were diagnosed with benign or malignant breast disease and were randomly assigned to receive either their normal diet alone or their diet daily supplemented with a 60 g soy bar. The challenge lasted for 14 days. Samples of normal breasts were biopsied and labeled with [^3^H]-thymidine to detect the number of cells in the S phase. The proliferation antigen Ki67 was searched for via immunocytochemistry. Genistein, daidzein, equol, enterolactone, and enterodiol were measured in serum samples collected before and after the soy intake. The Isofl concentrations of genistein and daidzein increased in the soy group, as measured at day 14. A strong correlation between Ki67 and the thymidine labeling index (r = 0.868, *p* < 0.001) was recorded. The soy supplementation significantly increased the proliferation rate of breast lobular epithelium cells at day 14 d when an adjustment was performed on the day of menstrual cycle and the age of the patient. The expression of the PRs was increased significantly in the soy group.

A third trial was published by Hargreaves et al. [134] a year later. It recorded the effect of 14 days of dietary supplementation with 60 g of soy containing 45 mg of Isofls (genistein + daidzein) in aglycone equivalents on the normal breasts of 84 pre-menopausal patients. The plasma concentrations of genistein, daidzein, and equol were shown to increase after soy supplementation to reach 261 ± 266 ng/mL, i.e., ≈1 µM (*p* ≤ 0.025). Nipple aspirate (NA) levels of genistein and daidzein were higher than paired serum levels before (*p* < 0.001 and *p* = 0.001, respectively) and after soy intake (*p* < 0.001 and *p* = 0.049, respectively). NA levels of apolipoprotein D decreased significantly, and pS2 levels increased with soy supplementation (*p* ≤ 0.002). These markers denote an estrogenic stimulus. The authors did not observe any effect of soy on breast epithelial cell proliferation, ER and PR status, apoptosis, mitosis, or Bcl-2 expression. They concluded that short-term dietary soy had a weak estrogenic effect on the breast and that no anti-estrogenic effect of soy on the breast was detected at the doses tested.

In all trials, Isofls were tested in a soy protein isolate. The active dose seemed to be in the range of 45 to 50 mg/day of Isofls in aglycone equivalents. Such doses are known to induce an Isofl plasma concentration between 1 and 1.5 µM.

Conversely, high doses of Isofls in food supplements tend to decrease mammary density in pre-menopausal women, as shown by Lu et al. [135]. The authors recruited 98 controls and 99 treated participants who received 136.6 mg of soy Isofls in aglycone equivalents 5 days/week for up to 2 years. Changes in breast composition were measured at baseline and at yearly intervals via magnetic resonance imaging. Adherence to treatment was estimated based on regular blood measurements. Globally, mammary density, measured via the percentage of fibroglandular breast tissue (FGBT) at imaging, tended to decrease with soy Isofl intake. Hence, after an average of 1.2, 2.2, and 3.3 years of treatment, the FGBT% decreased by 1.37, 2.43, and 3.50%, respectively, when comparing Isofl exposure with the placebo. Daidzein appeared to be more efficient than genistein. The data were processed and adjusted for confounding factors before analysis. The effect seemed weak, and the ingested dose was high, corresponding to 5 to 6 soy portions/day.

Meanwhile, a study by Maskarinec et al. [136] showed no effect of Isofl supplementation at 10 mg/day on mammary density. Treated and control subjects were compared. In the study, which lasted 1 year and in which compliance was assessed via urinary Isofl and tablet counting, the authors were able to collect full data on 30 subjects only. Mammary density, which was assessed using follow-up mammograms, was not significantly modified by the treatment.

Another 2-year-long trial involved 201 pre-menopausal women (98 treated and 103 controls). The treated women were fed 50 mg of Isofls in aglycone equivalents daily through soy foods [137]. This trial did not reveal a significant effect of the treatment on mammary density. Lifetime soy intake was investigated using a questionnaire, and breast density was determined in screening mammograms at baseline and at the end of the trial. After 2 years, the mean percentage density had decreased by 2.8 and 4.1% in the treated and control women, respectively. Women who reported eating more soy showed higher densities than women who ate little soy. This difference was significant only in Caucasians. The authors also reported that lower soy intake in early life and higher soy intake in adulthood predicted a greater reduction in the percentage density during the study period.

A comprehensive review of 18 randomized control trials (RCTs) was published in 2021. The studies analyzed were conducted on healthy subjects on Isofls and considering BC risk factors [138]. In the studies retained, Isofls were administered through soy foods or supplements in amounts varying from 36.5 to 235 mg/day and for periods lasting from 1 to 36 months. Breast density was one of the biomarkers followed. However, in most of the studies, differences between the Isofl and control/placebo treatments were not detectable. Globally, compliance with Isofl treatment was found to be good, and a lack of a significant effect was seen irrespective of the kind of intervention, the dose of Isofl used, or the duration of Isofl treatment.

Finally, although breast density is an independent biomarker of BC risk, the clinical trials that involved pre-menopausal women generally concluded that Isofls had an estrogenic effect on breast tissue when Isofls from soy were ingested at plausible dietary levels, i.e., one or two industrial soy portions/day. In any case, dietary interventions showed an anti-estrogenic effect and a preventive effect on BC risk. In such studies, it remained very difficult to distinguish the effect of pure Isofls from that of soy, possibly because the doses of Isofls that were used were sufficient to counteract a potential preventive effect of other soy components.

### 4.2. Clinical Effects of Isoflavones on Breasts of Post-Menopausal Women

In 2015, the European Food Safety Authority (EFSA) gathered a working group to determine if Isofls could have deleterious effects on menopausal women. The scientific report produced [139] showed that the findings with respect to the mammary gland were non-threatening. Because Asian diets likely contain extra soy and Isofls, the panel only considered studies involving Western women. Considering the interventional trials included, which gathered 816 women, the expert panel concluded that there were no breast density or histopathological changes induced by soy Isofl/soy extracts, soy protein, daidzein-rich Isofls, genistein, or clover extracts. Based on the studies examined, the EFSA panel concluded that there were no observable adverse effects on the mammary gland in healthy post-menopausal women undergoing Isofl treatments. Table 6 summarizes the data from the studies retained by the EFSA panel.

Most of the studies performed on post-menopausal women did not consider the time since menopause. However, as the timing of estrogen treatment is considered crucial, no significant effect was expected to be seen in interventional trials dealing with the effect of Isofls on mammary tissue in post-menopausal women. Considering trials involving clover, it is important to note that the Isofls in this legume are methylated on the fourth carbon and require a hepatic transformation to be active [150]. Consequently, the bioavailability of genistein and daidzein in women’s plasma is lower than that in soy Isofls, and, again, an effect was not expected owing to the dosages applied. Moreover, the vast majority of the interventional studies involving women considered a current diagnosis or a history of BC as exclusion criteria. Therefore, the conclusion of the EFSA panel could not be applied to this subpopulation. Finally, considering the available data, the EFSA panel mentioned that it could not establish a clear statement for peri-menopausal women or for post-menopausal women with a current diagnosis or history of estrogen-dependent cancer. In addition, many studies on the effect of Isofls on physiological issues in post-menopausal women have been published. They did not notice an effect on breasts, but they were not designed for this purpose.

### 4.3. Clinical Effects of Isoflavones on Estrogen-Positive Breast Tumors

To the best of our knowledge, only one study on the effects of Isofls on female BC cells in vivo has been published so far, namely, the study by Shike et al. [151], which examined the effects of soy Isofl supplementation on breast-cancer-related genes and pathways. The study gathered 140 women recently diagnosed with early-stage BC. These women were randomly assigned to soy protein supplementation (*n* = 70) or placebo (*n* = 70) groups for 7 to 30 days depending on the time between diagnosis and surgery. Compliance with the treatment was determined via plasma genistein and daidzein measurements. Gene expression changes were evaluated using NanoString in tumors before and after ablation. Genome-wide expression analysis was performed on the post-treatment tissue. Biomarkers of proliferation (Ki67) and apoptosis (Cas3) were assessed via immunohistochemistry. Compliance was not optimal in the study, as evidenced by the levels of Isofls in the women’s plasma. Indeed, the lowest Isofl concentration was recorded in a volunteer from the treated group. Nevertheless, the study showed that globally, the levels of Isofls rose in the plasma of the soy group and did not change significantly in the placebo group, wherein the levels were not null. In an analysis pairing samples collected before and after food treatment, 21 genes (out of 202) exhibited altered expression. Some genes, such as FANCC and UGT2A1, were modified in terms of the magnitude and direction of their expression. A genomic signature obtained from a microarray analysis of tumors was associated with a high genistein intake and plasma concentrations. It consisted of 126 differentially expressed genes. This signature included an overexpression (>2-fold) of cell cycle transcripts, including genes promoting cell proliferation, i.e., FGFR2, E2F5, BUB1, CCNB2, MYBL2, CDK1, and CDC20. Because of non-optimal compliance and the short duration of treatment, Isofls did not induce statistically significant changes in Ki67 or Cas3 between the soy-treated and control groups. However, an increase in biomarker levels was observed when comparing pre- and post-treatment tumors in the soy-treated group. Finally, soy intake and high genistein levels in plasma were associated with the overexpression of FGFR2 and genes that drove cell cycle and proliferation pathways. These data contrasted with the effect of high doses (>10 µM) of in vitro Isofls on cell apoptosis [34,35], and they raised the concerns that soy Isofls could adversely affect gene expression in these tumors in women suffering from estrogen-dependent breast cancer.

## 5. Soy or Isoflavones and Breast Cancer in Population Studies

When performing population studies with estrogenic substances, there are many endocrine-related confounding factors to consider, and because Isofls are of nutritional origin, there are also many dietary factors that should be taken into consideration since they are known to be positively or negatively linked to soy intake and can modify the risk of BC. From an endocrine point of view, the risk of BC in women is affected by genetic traits that can be estimated by examining family histories of breast diseases and considering exposure to reproductive hormones, especially estrogens [152]. Therefore, an early full-term pregnancy has protective effects [153], while the risk increases with the number of menstrual cycles experienced during one’s lifetime. The risk is also increased by shorter cycles, a younger age at menarche, and a late onset of menopause [154]. A link between the number and duration of lactations and BC risk has not been demonstrated. Exposure to xenoestrogens is also a deleterious factor, as seen with medical treatments such as hormonal contraception or hormone replacement therapy or through exposure to environmental endocrine disruptors [155]. Exposure during the perinatal period has a particularly strong effect on BC risk [156]. Furthermore, when dietary issues are considered, it should be noted that some food components (such as fibers), fruits, vegetables [157] (including cruciferous vegetables [158]), green tea [159], and adherence to a Mediterranean diet [160] were shown to reduce the risk of cancer, including BC, even if the negative association is sometimes only suggestive. However, high-fat diets, meat [161], high-fat cheese [162], and alcohol-containing diets [163] have been associated with an increased risk of BC. Moreover, socioeconomic factors [164], BMI at the onset of menopause [165], lack of physical activity [166], alcohol, and tobacco have been shown to enhance the risk of BC. The age of the subject is generally associated with certain types of cancers and can be used as a criterion with which to decipher the effects of soy or estrogenic Isofls. Moreover, some studies have also sorted BC according to its clinical classifications, i.e., ER, PR, and HER2 status. These are characteristics of the tumors and should be considered separately. However, when subgroups are formed according to menopausal status or receptor status (the common practice), the number of cases in each subgroup lowers, reducing the power of the analysis and its significance. In all studies dealing with chronic diseases and nutritional factors, researchers adjust their data based on the age of the participants and energy intake. However, when the effects of soy and Isofls on BC are examined, the confounding factors for which adjustment of the data from population studies would ideally be required must be considered. These factors are listed in Table 7.

The numbers listed in Table 7 are used in the following tables to evaluate the reliability of the population studies dealing with the effects of soy and/or Isofls on BC risk. On top of this list, all the studies analyzed adjusted their data on age and energy intake. As will be seen later, few population studies actually adjusted their observations according to all these confounding factors.

### 5.1. Limits of Population Studies

In Asian countries, specific food frequency questionnaires (FFQs) were developed to assess consumers’ soy and Isofl intakes [167]. They were used in population studies [168]. Some of these FFQs were validated either through reiteration—correlating responses between a first inquiry and other successive inquiries [169]—or comparison with urine or serum Isofl levels [170]. Usually, the correlation between two retrospective FFQs is around 50% at best; this can be explained by the time between the inquiry and the period of life investigated [171]. Similar correlation levels were recorded with Isofl measurements in biological fluids [167], and they seem rather low. Hence, when Asian soy foods were recorded in these studies, it can be assumed that the Asian soy intake was reasonably well determined. However, when Isofl were considered, the low correlation between putative intake and biological measurements in human tissues may indicate that the estimation is not optimal. Because exposure to soy and Isofls is considered constant in Asian countries, a steady-state level of Isofls would be expected in biological samples. Because exposure to soy and Isofls is considered constant in Asian countries, a steady-state level of Isofls would be expected in biological samples. However, this is not the case, and the poor correlations between soy intake and blood levels of Isofls could be explained if a significant proportion of the soy consumed was still prepared at home and in a traditional fashion. In doing so, Asian people would be applying water treatments, which would reduce the Isofl levels in their foods. Unfortunately, this is not the case when soy is prepared at industrial scales, wherein using tons of water can be tricky [16]. Therefore, at least three biases can undermine the Asian studies on soy and Isofl consumption. The first one is an overestimation of the Isofl intake if it is estimated from measurements taken cotemporally from industrially prepared soy foods. The second one is memory failure, preventing the estimation of soy intake a long time before an inquiry. The third bias is the evolution of the preparation processes over time. The quality of and, most probably, the Isofl concentrations in soy foods traditionally prepared at home have been modified. This bias should not be underestimated when researchers are trying to determine the effect of soy intake among adolescents on the risk of BC at the onset of menopause.

Conversely, in Western countries, where soy products are largely industrially produced, the estimation of Isofl intake is not compromised by this bias. However, aside from the soy foods identified earlier and declared in the FFQ assessing soy intake, there is also hidden soy incorporated in many transformed foodstuffs. While soy foods are generally consumed occasionally in Western countries, the contribution of Isofls from hidden soy to phytoestrogen exposure is proportionally high. Failing to account for this source of soy can induce a major bias in Isofl exposure estimation, compromising the correlation between soy records and Isofls in biological fluids [172] and potentially affecting the accuracy of the stratification of a cohort into subgroups. Additionally, earlier in this review, we mentioned that Isofls can have an effect on breast development in pre-menopausal women when the intake of Isofls (aglycone equivalents) reaches 45 to 50 mg/day [132,133,134]. However, in Western countries, such a dose is usually reached only occasionally or with food supplements. Thus, when the population is stratified, only a few subjects from the upper quartile can potentially be affected by their Isofl intake, and they may be hidden by the majority of the group. Finally, the estimation of Isofl intake seems to be deeply biased in both Western and Asian studies for various reasons.

As explained previously, investigating the effects of soy on human health requires accounting for genetic, hormonal, and environmental factors (endocrine disruptors and diet patterns) because Isofls have endocrine effects. As mentioned previously, the female populations studied are usually very heterogenous even if classified in the same category. Indeed, the hormonal response varies greatly in women while menopause progresses. Therefore, according to Monier et al. [173], a relationship between soy or Isofls and health issues should only be considered relevant if the *p*-value is <0.005. Indeed, if the estimation of intake is biased, many confounding factors are missed, or the population observed is very heterogenous, all results from the corresponding meta-analysis would have to be considered cautiously.

### 5.2. Case–Control Studies Involving Soy and Isoflavones

The oldest case–control studies on this issue essentially dealt with soy intake. In the previous century, Isofl assays were not frequently performed, and it is plausible that industrial soy foods were not as widespread as they are now. Thus, if it is assumed that homemade soy foods created following traditional recipes contained less Isofls than industrial ones [16], a pure soy effect would have been more likely to have been seen 30 years ago. When Isofls were included in these analyses, it was essentially through an estimation calculated via measurements gathered from databases. These measurements were made using commercial products and may not completely reflect the composition of traditional homemade soy foods. Furthermore, it appears crucial to use databases adapted to the potential food items eaten by the subjects. Thus, a Japanese database should be used in Japan, just as a Chinese database should be used in China. Additionally, measurements made a long time before or after exposure may be irrelevant. This could be the case when assessing Isofl exposure during adolescence with respect to post-menopausal women. Indeed, because the industrialization of food production has progressed globally, it might be erroneous to assume that the tofu eaten 40 years ago is the same as that eaten nowadays.

Table 8 shows that the effects of soy and/or Isofls were not always consistent.

**Figure 2:**
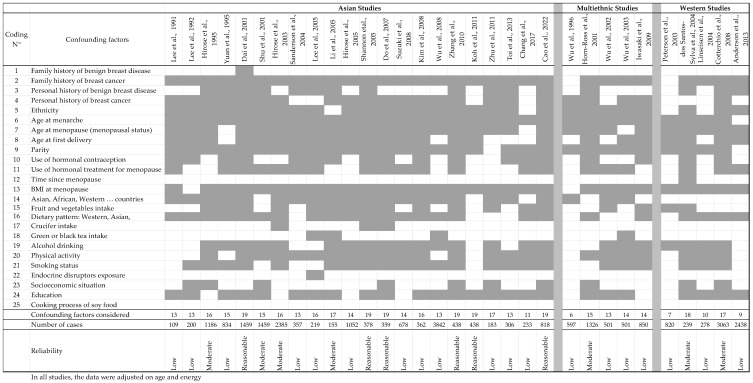
List of relevant confounding factors that should be taken into consideration when analyzing results from case–control studies on soy and/or isoflavones, and breast cancer risk, and the factors analyzed in the different studies. The studies mentioned in the tables are [21,174,175,176,177,178,179,180,181,182,183,184,185,186,187,188,189,190,191,192,193,194,195,196,197,198,199,200,201,202,204]. Finally, a reliability score was assigned by combining the number of cases included in the studies and the missing confounding factors.

Table 8, all studies considered the women’s age and energy intake to be potential confounders. Nevertheless, several confounders were hardly or never taken into consideration, as seen in Figure 2, including family history of benign breast diseases, time since menopause, exposure to endocrine disruptors, and origin of the soy foods eaten (commercial or homemade). In addition, crucifers and tea consumption were only recorded a few (two or three) times.

In Table 8, the studies are sorted based on studied population, Asian, multiethnic, or Western populations, because exposure to soy and Isofls appeared to be quite different in terms of quality and quantity. Finally, most of the studies were of low quality, often having a low number of subjects and numerous missing confounders (Figure 2). Looking closer at the results, the most recent studies seem to be the most reliable, taking into account more confounding factors and gathering more subjects. Unfortunately, during data interpretation, the authors often split their population according to menopausal or receptor status, which significantly decreased the reliability of their conclusions while decreasing the number of subjects. On some occasions, especially in Asian studies, no *p*-value was given.

Hence, in the earliest studies performed in Singapore [174,175], soy consumption was rather low, with soy food intake being assessed at a maximum of 55 g/d. In these studies, the amount of soy protein consumed was estimated at less than 4 g/d. However, in studies published a few years later, the soy food intake in China was 5 or 6 times higher [177,178,181], as was the soy protein intake. While the first few studies showed a preventive effect of soy on BC risk, this was no longer the case afterward, in studies with higher amounts of soy intake. In this context, the study by Zhu et al. [191] showed low reliability, reporting soy consumption at the same order of magnitude as that in previous Chinese studies. The number of subjects was low, and several confounding factors were not considered (parity, breast feeding, hormonal treatments for contraception or menopause, endocrine disruptors, etc.). Moreover, the study showed a protective effect of soy on ER+PR+ BC but no effect on other types of BC, indicating that soy and Isofls estimated in the range of <7.56–>28.83 mg/d could act as anti-estrogens. In 2022, Cao et al. [194] showed a protective effect of soy associated with fruits and vegetables on ER−/PR− BC but not on ER+/PR+ BC. While this result is in conflict with previous studies, it showed that the effect of soy can be affected by the concomitant consumption of healthy foods. Therefore, it should be noted that the more recent the studies, the better reliability due to the more comprehensive assessments of confounding factors.

The deleterious effect of high soy consumption was confirmed in the study by Lee [182] conducted in Taiwan, where the soy food intake was 10 times higher than that recorded previously in Singapore. In the study, soy consumption was associated with an increased risk of BC, although this association was not significant and many confounding factors were not considered. Later, Chang et al. [193] reported that soy associated with a vegetarian diet was protective against BC. In that study, the highest consumers consumed soy foods more than once a day. However, many confounding factors were not considered in that study, and its reliability remained low.

Looking at data obtained in Japan, the same tendency to increase soy consumption was observed. In 1995, Hirose et al. [176] fixed their highest tertile at a consumption of three soy foods/week, while in 2003, the highest quartile was >five times/week [180]. In 2005, the study by Hirose reported a highest soy consumption at around 56 g/d [18]. In that study, based on dietary recalls from the previous year, tofu was considered protective against BC, while fried tofu was not, indicating that the protective effect of soy and/or Isofls was limited and counteracted by fat intake. This finding also means that the effects of soy or Isofls, if any, can be easily masked by those of other food components.

Two studies were retained here that were performed in Korea. It is difficult to address the total amount of soy and Isofl consumed from these studies since they looked at peculiar soy-foods items only, without indication on other soy-food items. Nevertheless, the first study [185] showed that soybeans and soy paste have potentially protective effects, while soy milk, soy curd, and total soy do not. This would suggest that Isofls are not protective substances since they are present in all soy foods. The study by Kim et al. [187] showed a protective effect pre- but not post-menopause. However, some confounding factors such as family history of BC were not addressed, and the numbers of cases and controls were low, especially after stratification based on menopausal status.

Multiethnic approaches allow for a comparison of a vast range of soy and Isofl consumption amounts, optimizing the chance to observe an effect. These studies were mainly performed in the USA [195,196,197,198], except one involving Japanese and Brazilian subjects [199]. Soy appeared to have a protective effect against BC in four out of five studies. The only study that did not show a preventive effect was [196], which reported low levels of soy and Isofl intake. Isofl exposure was estimated based on tofu and miso consumptions. The frequencies were classified as zero or ≥once per week, and the highest median of Isofl intake was 2.775 mg/day. The reliability of the study was considered to be moderate based on the number of participants and the confounding factors considered. At this dose, phytoestrogens are not likely to exhibit any effect.

Regarding the other studies, a progressive increase in soy consumption was again noticed. In the study by Wu et al. [195], the highest tofu intake was over 55 times/year and the lowest was below 12 times/year. Later, in 2002, the same team recorded a highest intake at more than four times per week and a lowest below once a month [197]. In that study and the following one [198], Isofl intake was estimated, with the highest median intake at 12.88 mg /1000 kcal and a range of 20 to 25 mg of Isofls/day. This dose has not been shown to exhibit any effect on women’s breast. However, soy intake can be associated with a more vegetarian dietary profile as well as higher green tea consumption, which have been shown to have preventive effects on breast cancer. Based on the number of subjects involved and the confounders missed in the analyses, these studies were considered to be of low reliability. Finally, the study by Iwasaki et al. [199] should be analyzed. Their estimations of soy and Isofl intake were based on dietary recall from a previous year and on the Isofl concentrations recorded for commercial soy foods. The study only analyzed miso and tofu consumption in paired cases and controls, with 390 Japanese, 81 Japanese Brazilian, and 379 non-Japanese Brazilian participants. The highest median intake of Isofls was 71.3 mg/day, which may be proliferative on breast cells. This study showed that the higher the soy and Isofl intake, the lower the risk of BC. However, the lowest Isofl intake was observed on Brazilian women whose BMI was significantly higher, meat intake was higher, and vegetable consumption was lower. The analysis did not adjust the data for these parameters, conferring low reliability to the study. Finally, the highest Isofl intake was recorded in Japanese women based on a database established on commercial soy foods. In Japan, a significant proportion of menopausal women prepare their soy foods at home with lower Isofl concentrations, but this fact was not assessed.

In the studies performed on Western populations and listed in this review [200,201,202,203,204,205], the exposure of the populations to soy and Isofls was low, and in all those studies, the majority of the population was estimated to be exposed to less than 1 mg/day of Isofls. Only the study by Cotterchio et al. [204] reported a maximum intake of 158 mg Isofls in the fifth quintile of population. In all these studies, soy or Isofl consumption was not associated with BC risk except in the study by Anderson et al. [205], which showed an increase in the risk of ER−PR− BC with low doses of Isofls. However, the reliability of that study remains low, especially because stratification of the population based on estrogen receptor status resulted in a low number of subjects in each subgroup.

Scientists have tried to determine the effect of soy and Isofls based on menopausal status; however, the effect remains unclear. In Asian populations, four studies showed a protective effect pre-menopause [176,180,187,189], while one showed a protective effect post-menopause [191]. Only the studies in [176,189] had moderate and reasonable reliability, and both advocate for a preventive effect pre-menopause.

Regarding the receptor status of tumors, three studies reported a preventive effect on PR+ BC risk [178,186,191], while one reported a preventive effect on ER− risk [194] and another reported a deleterious association of low Isofl exposure with ER−PR− BC [205]. However, case–control studies cannot really be used to resolve this lack of clarity, especially because the reliability of those studies was either low or reasonable at best.

As mentioned previously, when soy or Isofl intake is estimated in the adolescence of pre- or post-menopausal women, the level of reliability is low due to memory recall bias and probably a difference in the quality of soy foods eaten now compared with in the past. Even if soy intake can be reasonably well estimated, this is not the case for Isofls, which definitely impedes conclusions from being made.

### 5.3. Observation Studies on Soy

Based on Medline and previous metanalyses [206,207,208,209], the main cohort studies conducted on the effect of soy or Isofls on BC risk were analyzed. In the following table (Table 9), Isofl exposure was always estimated from FFQs and databases but not measured in biological fluids. The preparation mode of soy food was not queried.

**Figure 3:**
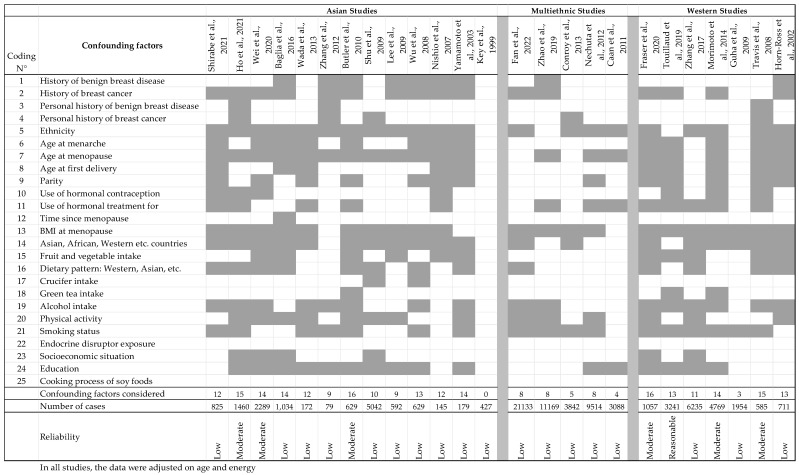
List of relevant confounding factors that should be taken into consideration when analyzing the results from population studies on soy and/or isoflavones and breast cancer risk, and the factors analyzed in the different studies. The studies cited in this table are [168,208,210,212,213,214,215,216,217,218,219,220,221,222,223,224,225,226,227,228,229,230,231,234,235] Finally, a reliability score was assigned by combining the number of subjects included in the studies and the missing confounding factors.

Based on the confounding factors considered in these studies and the number of subjects involved, the reliability of the studies was either low or moderate except for the study in [228] (see Figure 3 below).

Figure 3 shows that some confounding factors, such as cooking process for soy foods, endocrine disruptor exposure, crucifer consumption, green tea intake, time since menopause, and family history of benign breast disease, were never or hardly considered. For endocrine disruptor exposure, knowing if the women were exposed to pesticides or anthropic substances with hormonal effects (in factories, for instance) could be of help. Consequently, of the 25 studies analyzed here, 6 were considered to have moderate reliability and 1 reasonable [228]. That one study is the only one conducted on the effect of soy-based food supplements on BC risk. It allows the effect of Isofls to be compared with that of soy. However, because the composition of food supplements was varied, no quantitative association with Isofl intake could be concluded, thus resulting in the study having only reasonable reliability.

The study in [222] was essentially performed to determine whether soy intake has a protective effect against BC induced by atomic bomb radiation in Nagasaki. However, the study did not adjust the risk for any other confounding factors, probably because the authors considered that the effects from radiation would be far greater than that from any other factors. Moreover, multiethnic studies usually adjust their data on only a few confounders, possibly explained by a difference in cultures, influenced the nature of their inquiries and willingness to answer.

The high proportion of studies with low reliability is in accordance with that found in all meta-analyses [206,207,208,209] published so far, which have indicated that better-quality studies are required for fully convincing conclusions. The consumption of dairy product was inversely correlated with soy intake and may be considered as another confounding factor to adjust for [227]. These confounding factors can either impact the reliability of the cohort stratification or the outcome occurrence. That said, this induced strong heterogeneity in the studies, preventing conclusions from being formed based on a formal meta-analysis [173]. Looking at the putative results of the above 25 studies, 11 tended to indicate an association of soy or Isofls with a reduced BC risk. However, in some cases, only the effect of soy was significant [208]; in most studies, there were other strong dietary confounding factors which were not considered [223,224,225,226,227,228,229,230,231,232,233,234,235]: dietary patterns, fruit and vegetable consumption, dairy product intake, and green tea and crucifer consumption. In addition, 10 studies did not notice any significant effect of soy or Isofls on BC risk, and 2 reported a mild increase in BC risk. In some cases, a positive effect is noticed only in pre-menopausal women [213], while in others, in post-menopausal women [216,219,231]. Some studies report a preventive effect on ER+ BC [213], while others report a preventive effect on ER− BC [229]. Moreover, in some studies, not all types of soy Isofls had protective effects [231]. In addition, when the effect of early soy or Isofl exposure was examined, the risk of memory bias on dietary recall was greater, as was that of changes in food quality [213,218]. This is particularly true for Isofls, since 40 or 50 years ago, the availability of industrial soy foods was not the same as that nowadays and the quality of commercial soy foods may be different from that cooked at home.

Finally, it remained very difficult to separate the effect of soy itself from that of Isofls. The study by Touillaud et al. [228] is the only one that seriously considered Isofl exposure through food supplements, showing an increased risk of ER− BC, as was also concluded by Anderson et al. [205] in their case–control study. However, the number of ER− cases was low, and some supplement users may have been missed in that study. In addition, the protective effect of Isofls on ER+ BC is just significant. Thus, a deleterious effect of Isofls on ER+ BC cannot be excluded. Finally, 1215 women with a first diagnosis of BC were excluded for one or more reasons, which could have led to misleading conclusions.

Considering BC recurrence, three cohort studies performed in Asia tended to show a lowering effect of soy consumption and exposure to Isofls. However, all these studies were considered to be of low reliability because they missed potential strong confounders. Since phytoestrogens can enhance breast cell multiplication at intake over 45 mg/day, a deleterious effect of Isofls could be expected at high soy intake. Therefore, regarding the studies on BC recurrence, of note is that the subjects involved only had a simple tumor ablation or a mastectomy. Indeed, if light surgery was employed, as in the case in France, as a first intention to treat, the risk of leaving tumor cells that could induce further recurrence is higher. If these tumor cells are ER+, a proliferative effect of estrogenic Isofls at doses over 45 mg/day cannot be excluded. Mastectomy is definitively better at preventing recurrence in survivors.

### 5.4. Population Studies Conducted Specifically on Isoflavones

Several case–control studies have been performed using biomarkers of Isofl exposure to ascertain Isofl intake in order to reduce uncertainties in their estimation. Historically, Isofls were first assayed in urine samples since their concentrations are higher in urine than in plasma and, therefore, more accessible using techniques with medium sensitivity, such as HPLC-DAD [236], HPLC-Coul array [237], or GC-MS [238]. Moreover, such assay techniques have become more sensitive over the years and are now sensitive enough to assay Isofls in serum. Of note, the portion of Isofls in plasma is more relevant, since it reflects the concentrations of Isofls found in the vicinity of target cells. Conversely, Isofls in the urine are eliminated Isofls that no longer act in vivo.

#### 5.4.1. Isoflavones in the Urine

In the urine, Isofls follow a pharmacokinetic profile and their concentrations fluctuate with time. In human beings [30], the T*_max_* of daidzein and glycitein is 12.7 h, while that of genistein is 15.7 h. After this time-point, Isofl urine levels decrease when human subjects are challenged with unique intake. Considering these pharmacokinetics, the daily intake of Isofls twice within a 24 h period would result in these substances arriving in the urine before the previously ingested one has yet to be eliminated. That said, a pharmacokinetic plateau can be reached, which stabilizes urine levels but only with chronic intake. This can be observed in Asian populations but not always in Western populations where soy consumption is occasional. Hence, a spot urine analysis on Western subjects may be at risk of misestimation of soy and Isofl consumption. This misestimation depends on the delay between ingestion and when sample collection occurs. This is why, to obtain a better correlation between urine levels and soy intake, the time to the last meals should be recorded [90] or urines samples should be taken overnight or first thing in the morning. The following Table 10 summarizes the results obtained in the seven studies on urinary Isofl and BC risk.

Except for the study by Ward et al. [242], with moderate reliability, all the other studies were considered to be of low reliability. This is because the number of subjects enrolled in these studies were generally low and many confounding factors were neglected, particularly ones known globally to be linked with dietary patterns. In addition, some other important factors, such as family history of BC, were not always evocated in these studies.

More precisely, the studies performed on Asian populations were not very reliable. In the study by Zheng et al. [236], no difference was found in soy intake, while there was one in urine levels. This result is surprising, since specialists have found that urine Isofl concentrations often reflect soy consumption well. The only explanation for this surprising result is that the excretion rate of Isofls is different between cases and controls. However, this difference was not reported by other authors. Thus, the study, which involved only 60 cases and controls, could be considered of low reliability. In another study, by Dai et al. [239], the authors subdivided their population of subjects in two groups: pre- and post-menopausal women. However, this subdivision caused the number of subjects to became too low, decreasing the power of the analysis. Additionally, the authors showed that the amount of enterolignans was higher in the control group, suggesting a higher consumption of fruits and vegetables, which is known to decrease BC risk. Therefore, the results obtained in that study could reflect a larger consumption of healthy foods other than soy by the control group. Finally, in the study by Dai et al. [240], the soy intake was higher in the controls than in the cases, explaining the difference in urinary Isofl levels between the two groups. However, the inverse association of Isofls with BC risk was only observed in women with BMI ≥ 25, E2 levels > 5.73 pg/mL, E1-S < 0.96 ng/mL, and SHBG < 81.4 nM. Additionally, the number of subjects was low.

Considering the studies performed on a populations of Western volunteers, the reliability is not optimal either. In the study by den Tonkelaar [241], the individual variations in urine concentrations of Isofls or enterolignans were analyzed as being high, indicating that urine samples could not be used reliably as a soy intake biomarker, since the pharmacokinetic parameters of these substances were not taken into account. In parallel, the study did not adjust its results on dietary patterns that could be strong confounding factors of BC risks. To reinforce previous results, Grace et al. [238] showed that the correlation of urine Isofl concentrations with soy intakes was low. As that study was performed in the UK, the intake of hidden soy could have been omitted, altering the estimation of soy intake. For this reason, the reliability was considered to be low, since the number or subjects involved in the study was also only 114. Finally, the study by Goodman et al. [243] reported urinary levels of daidzein which were about 5 times higher than those of genistein. Such profiles are quite unusual, especially in Western populations, where genistein intake is usually twice that of daidzein [244] and where the Isofl exposure is rather occasional. This might reflect an analytical problem, which of course reduced the reliability of the results.

#### 5.4.2. Isoflavones in Serum or Plasma

Like in urine, Isofl levels largely vary in plasma after unique intake [30], with a maximum concentration occurring after 8 to 9 h depending on the substance analyzed. When the dietary intake occurs twice a day, a pharmacokinetic steady state can be observed [245]. Such a phenomenon is likely to occur in Asian people, who are known to consume soy foods regularly, but may be less common in Western populations. Thus, one could expect that the Isofl concentrations in serum would be a better biomarker of soy intake in Asia than in Western countries. Nevertheless, several studies completed their case–control investigations with measurements of Isofl concentrations in plasma. Table 11 below summarizes their observations.

Four of the studies reported in Table 11 were considered to be of low reliability because they did not analyze the time of blood sample collection in relation to the time of last food intake. Therefore, the levels of Isofls in the blood, especially in Western populations, could be deeply affected by the pharmacokinetics of such substances. Additionally, major food patterns that influence BC risk were not considered.

The only study with a moderate reliability was that of Iwasaki et al. [247]. However, as mentioned for other studies, major dietary confounders were not assessed in their study, constituting a first limitation. The second limitation relates to the daidzein measurements. Surprisingly, the daidzein plasma levels used in that study were not in the average range of concentrations usually reported for genistein and daidzein when assessing Japanese soy intake [249], indicating a methodological problem with their Isofl assay. A methodological problem would explain why the authors did not find a link between the plasma levels of daidzein and BC risk. However, the genistein plasma levels in another study were in the range of those previously performed in intervention studies when dealing with human exposure to Japanese soy foods [250].

In addition, Lampe et al. [246] studied different levels of Isofl exposure in women from Shanghai, a methodology optimal for determining the effect of Isofls. When the concentrations were expressed in molar units, the levels recorded in the fourth quartile were 0.168 µM for daidzein and 0.285 µM for genistein. From these concentrations, only 10% were in aglycone form. Thus, the levels of estrogenic substances in the consumers’ blood were low, and no deleterious effect on BC could be expected from such exposures. In this context, the recorded effect may be associated either with soy itself, with Isofls being a biomarker of soy intake, or with other dietary or healthy life confounders. See Table 7 for details. Indeed, the study by Lampe et al. [246] did not consider any dietary characteristics, the consumption of tobacco or alcohol, or levels of physical activity, which are known to influence the BC risk.

In [238,242,248], performed in Western populations, exposure was low, and the correlation between plasma levels and recorded soy intake was for the best around 50%. This suggests a misestimation of the exposure to Isofls. However, when plasma levels are low, measurements of Isofls could be challenging and may require the use of tandem mass spectrometry. Nevertheless, no clear conclusion can be made from these studies since one showed an increased risk of BC, one reported no effect, and the third one revealed a reduction in BC risk. In all cases, the concentrations of Isofls that were determined after hydrolysis of the conjugated forms were much lower than those classically used in cell culture experiments. At such doses, no estrogenic action can be expected, and the observed effect would have to be analyzed in consideration of other confounding factors.

## 6. Discussion

The aim of this review was to determine the role of soy and of Isofls on BC risk.

### 6.1. In Vitro Data

Based on in vitro data, doses in the range of 0.1 to 1 µM of aglycone compounds could induce the proliferation of ER+PR+ BC cells. This effect was shown to be due to the ability of soy Isofls—genistein, daidzein, glycitein, and equol—to bind to the nuclear estrogen receptors ERα and ERβ [20]. These compounds were also shown to activate rapid cell signalization pathways involving either ERs at the cell membrane or GPER. However, only a few studies have thus far shown a response in ER− BC cells. These responses may be mediated by GPER, to which Isofls have a high affinity [29]. The nature of the response of the cell (proliferation or inhibition) is expected to be linked to the concentration of Isofls. At concentrations of Isofls lower than 0.1 µM, the estrogenic effect of Isofls is generally not observed. At doses over 5 or 10 µM, Isofls induce cell apoptosis. However, such concentrations would not be achieved in vivo. Therefore, depending on the concentrations reached in vivo, the data obtained in a cell culture would indicate that Isofls could be either estrogenic and induce the proliferation of ER+ breast cells or could have no effect when their concentrations are too low.

### 6.2. Animal Data

#### 6.2.1. Toxicological Studies

The data obtained in rat models in toxicological studies [60] indicated that Isofls may induce adenoma and adenocarcinoma of the mammary glands and the pituitary at plasma doses that can be achieved dietarily in humans. However, exposures to low doses were less conclusive, even if some effects were recorded on reproductive tissues and on fertility [61,251].

#### 6.2.2. Cancers Induced In Vivo

On rodent models bearing chemically induced mammary tumors, soy and Isofl consumption was shown to have various effects on tumor cells. In these experiments, the ER and GPER status of the tumor cells was generally unknown, and the effect of Isofls may have depended on the availability of these receptors. Nevertheless, neonatal exposure to relatively low levels of genistein, i.e., those present in dams’ milk, would be able to counteract the tumorization process induced by the cancerogenic compound DMBA when administrated after Isofls [65]. This effect may involve epigenetic regulations of either oncogenic or anti-oncogenic genes [73] or be due to an early maturation of the mammary gland in female pups [88,89]. This maturation process would induce mammary cell differentiation that would prevent later cell tumorization. The transposition of this beneficial effect from rats to humans is hard to achieve. When rats were exposed to Isofls later in life, the effects on chemically induced tumors were less clear, with either a proliferative effect, an inhibition, or no effect. This was also the case when Isofls were administered after the cancerogenic substances. However, in many cases, the circulating concentrations of Isofls were not available, and therefore, these results were difficult to be translated to humans.

#### 6.2.3. Humanized Models

Rodent models implanted with human cells allowed for the effect of Isofls to be checked in the conjugated forms present in plasma and lymph [31]. However, since rodents are universal equol producers, their reactions to soy and daidzein treatments could be different from those of human beings. Nevertheless, when immune-depressed rodent models were implanted with human BC cells, the effect was different according to the ER status of the tumor cells. In mice implanted with MCF-7 cells, genistein at plasma doses ≥ 0.7 µM and daidzein at doses ≥ 0.9 µM enhanced the proliferation of human estrogen-dependent BC cells. The two types of Isofls exhibited an additive effect. A negative interaction was also found with two drugs used to treat estrogen-dependent BC in women: tamoxifen (TAM) and letrozole. However not all mice strains reacted the same way, and the effect was dependent on the treatment duration. Isofls were tested as pure aglycone or as glycosides, as well as in food matrices. With the latter, the results were less clear, suggesting that other soy products could have an effect on breast tumors.

#### 6.2.4. Other Models

Monkeys are not the best models for humans since their Isofl metabolism is high and their sensitivity to such substances is low. In contrast, pigs, sows, and piglets have been shown to be more similar to humans, and the data collected from these models showed that dietary doses of genistein and/or soy Isofls induced the proliferation of mammary tissue in neonatal piglets and gilts. Genistein and daidzein also induced partial estrogenic effects on the reproductive tract at dietary doses.

Finally, in vivo animal studies showed that soy Isofls at doses achievable in human plasma could exert estrogenic actions and enhance the proliferation of BC cells. However, the effect of the soy matrix could be different.

### 6.3. Data from Intervention Studies in Women

In healthy pre-menopausal women, 45 to 50 mg of Isofls, which can be found in a mug of soy milk, a portion of soy burger, or a portion of industrial tofu, had estrogenic effects on estrogen-dependent proteins from the mammary fluid, on the volume of this fluid, and on breast cell proliferation. While no effect was recorded on healthy post-menopausal women, the experimental data were not sufficient to make any conclusions in healthy peri-menopausal women. However, in women with estrogen-dependent BC, the only study that was performed so far showed an increased expression of genes involved in cell proliferation. The protocol of that study could be improved, but considering the corroborating evidence available, such a study may not be authorized by ethical committees in the near future. Therefore, a proliferative effect of Isofls on breast tumor cells cannot be excluded.

### 6.4. Data from Case–Control Studies

A large set of case–control studies were analyzed in this review, several of which included urinary and/or plasma measurements of Isofls. However, no clear conclusion on Isofls’ effects could be drawn from the comparison. Some studies tended to show a preventive effect, while others showed no effect, and others found a deleterious effect.

These case–control studies probably allowed for a better analysis of the pure effect of soy compared with that of Isofls. Thus, soy consumption recently increased in Asian countries; however, the protective effect observed 30 years ago can no longer be found nowadays [175,182]. This change suggests that soy may be a healthy food but that its positive effect may be negated when Isofl exposure becomes too high. Such a conclusion should be ascertained by performing a full diet analysis since many confounders were present in the healthy diets recorded in the studies analyzed here.

To try to reduce the uncertainties linked to the estimation of Isofl intake, several authors performed their analyses with Isofl measurements in plasma and/or in the urine of their subjects. However, the number of subjects was often low, and many confounders were often omitted. Additionally, the correlations between food intake and Isofl measurements in biological fluids were generally low because the time between the subjects’ last meal and the sample collection was not taken into account. Only the studies where urine samples were collected overnight or in the morning could really allow for comparisons between groups and subgroups of populations [236,241,243]. Thus, most of the case–control studies including Isofl measurements in biological fluids were of low reliability and could not really be used to determine the effect of soy intake on BC risk. Hence, in the five studies involving serum markers of soy intake, two showed a preventive effect, one showed a deleterious effect, and two showed no effect. Similarly, among the seven studies that measured Isofl levels in urine samples, one showed a preventive effect of genistein associated with daidzein, two showed a deleterious effect of equol, and four studies showed no or partial effect or an effect of lignans but not an effect of Isofls. In addition, in some studies, some soy foods had preventive effects while other did not, even though they all had similar Isofl concentrations [18,185].

### 6.5. Data from Cohort Studies

Although quite a large number of cohort studies were gathered in this review, no clear conclusion could be made on the effect of soy and Isofls on BC, largely due to the poor quality of the studies. Finally, some studies indicated a protective effect of soy, while others showed a deleterious effect of Isofls and others showed no effect. Indeed, although soy and Isofls would fall into the domain of food, Isofls affect reproductive physiology and pathology. Therefore, confounding factors belonging to these two scientific areas should be taken into account. This review showed that this is hardly the case. Hence, the reliability of the studies remained low. As mentioned in this study, exposure to soy is reliable in Asian countries but not in Western countries, where soy is present in many transformed food items at doses close to those estimated from soy foods. Hence, while consumers of hidden soy can absorb a few mg of Isofls/day, because soy is only consumed occasionally in the West, hidden soy is often missed, thus inducing an important bias in such studies. Similarly, in Asia, the method of food processing can impact the concentration of Isofls in soy foods, but such information is never recorded. Again, this bias can prevent the determination of the effect of soy and/or Isofls on breast cancer. Nevertheless, in some studies, the authors reported a preventive effect of soy but not Isofls on BC risk [216,217], perhaps because Isofls are not involved in this process or the estimation included an uncertainty that altered the significance of the results.

In this context, the study by Chen et al. [12], which used data mining to explore the relationship between the intake of soy, fibers, Isofls, or proteins/peptides and BC development, found interesting results. Soy fibers; proteins; peptides such as lunasin; and the protease inhibitor Bowman–Birk, had positive impacts on various types of BC. However, according to the study, soy Isofls exhibited an inconsistent effect on BC development. In the 478 data points collected from 201 publications, daidzein exhibited the highest negative impact on BC, followed by soyasapogenol, genistein, and equol. In the present review, a similar conclusion could be reached since soy matrices were found to induce different effects on BC compared with the effects of Isofls alone in animal and/or human studies. Hence, when athymic mice or population studies were analyzed, a protective effect of soy could not be excluded [97,110]. However, regarding the large number of studies on genistein, this type of Isofl could not be concluded to be less harmful than daidzein. Moreover, in the oldest studies performed in Singapore reporting low soy intake with low levels of Isofls, a significant reduction in BC risk was found, although these investigations were of low reliability [174,175]. This preventive effect was no longer observed in later studies with better reliability when the amounts of soy and Isofl intake were increased [176,178]. Finally, Zhao et al. [208] found that soy was associated with a reduced risk of BC, while Isofls were not.

In Western countries, the exposure is too low to be accurately determined on biological samples unless pharmacokinetic parameters were used to correct blood or urine levels. Additionally, to achieve Isofl effects on breast tissue, doses of 45 to 50 mg/day would be required for several weeks [132,133,134], achievable with chronic consumption of food supplements or soy foods, though more rarely for the latter. In the population studies gathered here, for Western consumers, the population stratification in all subgroups was usually inadequate to highlight the regular consumption of foods containing high Isofl amounts. Additionally, when only a low consumption of soy products is achieved, the bias induced by Isofls from hidden soy is proportionally higher.

## 7. Conclusions

Soy Isofls are weak estrogens, but at dietary levels, they can reach plasma concentrations that can be 10,000 or 100,000 times higher than that of endogenous E2 [252]. At such doses, physiological effects can be expected since both E2 and Isofls are under conjugated forms in plasma. However, the effect of estrogenic Isofls on BC is still a subject of controversy. This review pointed out that at doses potentially occurring in human plasmas, Isofls induce the proliferation of estrogen-dependent BC cells in vitro. Such an effect was not observed so far on TNBC, even if Isofls can activate the GPER pathway. This review also recalled that the USA-NTP showed the induction of adenomas and adenocarcinomas of the mammary glands in Sprague Dawley rats exposed to genistein at human dietary doses in a multigenerational experiment. Based on studies performed on chemically induced tumors in rodents, this review pointed out that early exposure to low doses of Isofls could prevent BC in rodent models. However, in mice implanted with estrogen-dependent BC cells, Isofls induced tumor growth, indicating a preventive effect at early stages of tumorization and a proliferative effect later on. Soy was responsible for a preventive effect even when Isofls induced cell growth. While non-human primates were shown to be poor human models, pigs were found to be more similar, showing an estrogenic response to dietary Isofl treatments.

In humans, estrogenic effects have been found for dietary Isofls at doses of 45–55 mg/day in pre-menopausal women’s breast. Such doses can be achieved with one or two portions of industrial soy foods/day. In healthy post-menopausal women, interventional studies involving soy-based food supplements failed to show any deleterious effects, while some cohort and case–control studies did show an increase in ER− BC risk. While the higher risk of BC in women occurs peri-menopause, the data are insufficient to make conclusions about this specific menopausal status nor to exclude a deleterious effect in women with an already present estrogen-dependent BC. Finally, no consensus can be made based on the clinical data about the actions of Isofls on ERα+, ERβ+ PR-A PR-B, ER−, PR−, HER2+/−, or TNBC.

This review showed that population studies are not as reliable as they should be. Besides the many omitted confounders, the estimation of soy and Isofl intake could be improved. In fact, their misestimation limits the interpretation of all data, including retrospective investigations on exposure during adolescence. In the earliest population studies, when soy was probably less concentrated with Isofls, a protective effect on BC was noticed. However, the reliability of these studies was low. Later, when the intake of Isofls was estimated, its link with BC risk was not systematic and depended on the soy products considered, as well as on other food items. As mentioned by Chen et al. [12], all the data collected here indicate that soy foods with low levels of Isofls could be protective against BC. However, with the increased concentration of Isofls in modern industrial soy foods, soy no longer exerts a protective effect on estrogen-dependent BC risk.

Therefore, this review leads to a new interpretation. A soy matrix with its fibers, peptides, and protein components may prevent BC risk, while estrogenic Isofls could have a deleterious effect on estrogen-dependent BC. These antagonizing effects of different soy components mainly lead to inconsistent results when the amounts of soy and high Isofl intake are concomitant.

## Figures and Tables

**Table 1:** Effects of soy isoflavones on breast cancer cells in vitro.

Cell Types	Isoflavone Concentrations	Effects	References
MCF-7 ER+/PR+/HER2+/GPER+	Genistein 0.1 µM	Proliferation	[50]
	Equol 0.1 µM	Proliferation	[50]
	Daidzein 1µM	Proliferation	[50]
MDA-MB-231 ER−/PR−/HER2−/GPER+	Genistein 1 µM	Synthesis of cfos	[51]
	Daidzein 1 µM	Inhibition	[52]
	Daidzein 0.1 µM	No effect on growth	[52]
	Equol 1 µM	Inhibition	[52]
	Equol 0.1 µM	No effect on growth	[52]
SKBR3 ER−/PR−/HER2+/GPER+	Genistein 1 µM	Synthesis of cfos	[51]
	Genistein 1 µM	No proliferation	[53]
	Daidzein 1 µM	No proliferation	[53]
MDA-MB-468 ER−/PR−/HER2−/GPER+	Genistein 1 µM	No effect on growth	[54]
T47D ER+/PR+/HER2+/GPER+ Tam-resistant	Genistein 0.1 µM	Proliferation	[55]
	Daidzein 0.1 µM	Proliferation	[55]
	Equol 0.1 µM	Proliferation	[55]
ZR-75.1 ER+/PR+/HER2−/GPER+	Genistein 1 µM	Slight growth induction	[54]
	Daidzein 1 µM	No effect on growth	[53]

Regarding in vitro studies, only those which tested isoflavone concentrations ≤ 1 µM are mentioned.

**Table 2:** Summary of studies on the oral administration of soy and/or soy isoflavones to rats prior to the chemical induction of mammary tumorigenesis.

Ref	Animals	DMBA Treatment	Isoflavones	Isoflavones mg/kg bw/day	Duration of the Study	Tumor Incidence (%) *	Effect	Isoflavone in Plasma (µM)
[62]	Sprague Dawley Female rats 7 weeks old	PND50 80 mg/kg bw	Genistein	2.5	38 weeks Isofl G1–PND21	53	Inhibition	0.04
				25.0		23	Inhibition	0.45
[63]	Sprague Dawley Female rats 5 weeks old	PND50 50 mg/kg bw	Soy diet	3	18 weeks Isofl PND35–end	80	No effect	0.1 ^b^
				40		74	No effect	1.5 ^b^
				80		57	No effect	3 ^b^
[64]	Sprague Dawley Female rats 5 weeks old	PND50 50 mg/kg bw	Genistein	20	17 weeks Isofl PND43–end	90	No effect	0.9
			Daidzein	20		100	Inhibition	1
			Gen + Dai	10 + 10		100	No effect	1 ^a^
			Soy prot +	22 + 12		95	Inhibition	1.37
			Soy prot −	0		89	Inhibition	0
[65]	Sprague Dawley Female rats Age unknown	PND50 80 mg/kg bw	Genistein	2.5 G1–PND21	38 weeks	77	Inhibition	0.04 ^b^
				25.0 G1–PND21		53	Inhibition	0.45 ^b^
				25.0 G1–G21		100	No effect	0.45 ^b^
				25.0 PND0–21		53	Inhibition	0.45 ^b^
[66]	Sprague Dawley Female rats 6 weeks old	60 mg/kg bw Age unknown	Genistein	20	16 weeks Age unknown	40	Inhibition	0.45 ^b^
			Daidzein	20		50		0.6 ^b^
			Gen + Dai	20 + 20		20		1 ^b^
[67]	Sprague Dawley Female rats Age unknown	PND50 80 mg/kg bw	Equol-*S*	40 PND0–21	20 weeks	97	No effect	0.8
				22 PND21–35		97		0.65
			Equol-*R*	40 PND0–21		86		0.8
				22 PND21–35		86		0.65
[68]	Sprague Dawley Female rats Pregnant GD 11	Offspring PND50 40 mg/kg bw	Milk ± Isofl to pregnant dams	High: 28 GD11–GD21	18 weeks post-DMBA	75	Inhibition	0.12
				Low: 7 GD11–GD21		75	Inhibition	0.12
[69]	Zucker Female rats Lean 5 weeks old	PND50 65 mg/kg bw	Soy protein	140	15 weeks Isofl PND42–147	62	Inhibition	3 ^b^
	Zucker Female rats Obese 5 weeks old					100	No effect	3 ^b^
[70]	Wistar Female rats 6 weeks old	PND55 80 mg/kg bw	Genistein	2–3 times/w	20 weeks Isofl PND40–end	60	Inhibition	0.1 ^c^
			Genistein + Lycopene	2 + 20 3 times/w		40	Inhibition	0.1 ^c^
[71]	Donryu Female rats 5 weeks old OVX	PND35 50 mg/kg bw	Soy isolate	15	40 weeks Isofl PND35–end	100	Activation and estrogenic	Unknown
			Genistein 52%					
			Daidzein 42%					
			Glycitein 6%					
[72]	Sprague Dawley Female rats 6 weeks old	F1, PND50 100 mg/kg bw	Genistein	1: GD1–PND21	20 weeks	25	Inhibition	0.05 ^d^
			Genistein + Vinclozolin	1 + 1: GD1–PND21		50	Inhibition	0.05 ^d^
[73]	Sprague Dawley Female rats and 10-day-old female pups	PND48 25 mg/kg bw	± Genistein ± TAM	44	38 weeks Isofl PND15–30 s	100	Activation but delayed	1 ^e^
[74]	Sprague Dawley Female rats Age unknown	PND60 80 mg/kg bw PND90 40 mg/kg bw	Genistein Nanoparticles	0.2	20 weeks Isofl PND40–end	100	Activation	Unknown
			Microparticles	0.2		80	Activation	Unknown
			Macroparticles	0.2		100	Activation	Unknown

* Expressed as a percentage of corresponding control. ^a^ Estimated from [60]; ^b^ estimated from [59]; ^c^ estimated from [62]; ^d^ estimated from [75]; and ^e^ estimated from [76]. DMBA: dimethylbenz(a)anthracene; GD: gestation day; and PND: postnatal day.

**Table 3:** Summary of studies on the oral administration of soy and/or soy isoflavones to rats mainly after the chemical induction of mammary tumorigenesis.

Ref	Animals	Carcinogen Treatment	Isoflavones	Isoflavones mg/kg bw/day ^a^	Duration of the Study	Tumor Incidence (%) *	Effect	Isoflavone in Plasma (µM)
[65]	Sprague Dawley Female rats Age unknown	DMBA PND50 80 mg/kg bw	Genistein	37.5/ /25.0	38 weeks PND0-21/ /100–end	33	Inhibition	0.45 ^b^
[79]	Sprague Dawley Female rats Age unknown OVX	DMBA PND50 50 mg/kg bw	Genistein tumors +	1.5	43 weeks Isofl: PND133–385	140	Activation	0.04 ^a^
				15.0		45	Inhibition	0.40 ^a^
			genistein tumors −	1.5		165	Activation	0.04 ^a^
				15.0		170	Activation	0.40 ^a^
[80]	Sprague Dawley Female rats 20 days old	MNU PND21 75 mg/kg bw	Genistein	75	Isofl OVX-TD90	400	Activation estrogenic	3.1
[81]	Sprague Dawley Female rats 6 weeks old	DMBA PND50 25 mg/kg bw	Soy milk estimated 178 µg/g ^b^	18 ^c^	13 weeks Isofl PND51–end	100	Activation	0.35 ^a^
[82]	Sprague Dawley Female rats 8 weeks old OVX	DMBA 56 PND 50 mg/kg bw	Genistein Daidzein Equol	0.73 0.69 or 6.9 0.65	3 weeks PND57–end	100 33–46 30	No effect Inhibition Inhibition	Unknown
[83]	Sprague Dawley Female rats 7 weeks old	DMBA 57 PND 10 mg/kg bw	Soy extract Genistein 49.7% Daidzein 5.3% Glycitin 34.5%	Low: 8 Medium: 40 High: 80	24 weeks Post-DMBA Isofl PND71–end	26.7	Inhibition	0.4 ^c^
						6.7		2.0 ^c^
						6.7		4.0 ^c^
	Sprague Dawley Female rats 7 weeks old OVX			Low: 7 Medium: 35 High: 70		13.3	Inhibition	0.4 ^c^
						6.7		2.0 ^c^
						13.3		4.0 ^c^
[76]	Sprague Dawley Female rats and 10-day-old female pups	DMBA 48 PND 25 mg/kg bw	Genistein PND15-30	25 Gen PND55–end	38 weeks	100	Activation but delayed with genistein	0.45 ^c^
			Genistein + TAM PND15-30	TAM tumor–end 25 Gen tumor–end				
			Genistein	25 Gen PND55–end				
			Genistein + TAM	TAM tumor–end 25 Gen tumor–end				

* Expressed as a percentage of corresponding control. ^a^ Estimated from [60]; ^b^ estimated from [84]; and ^c^ estimated from [76]. DMBA: dimethylbenz(a)anthracene; MNU: 1-methyl-1-nitrosourea; PND: postnatal day; and TAM: tamoxifen.

**Table 4:** Summary of the effect of oral isoflavones on athymic nude mice implanted with estrogen-dependent human breast cancer cells.

References	Animals	Cell Lines Implanted	Treatment	Dosages mg/kg/day ^a^	Test Duration	Effect	Concentration in Plasma µM
[92]	Females OVX 5 weeks old	MCF-7	Genistein	150	12 weeks	Proliferation uterotrophy	2.1
[93]	Females OVX 4 weeks old	MCF-7	Genistein	2.4	10 weeks	No effect	0.1
				24		Proliferation	0.7
				48		Proliferation	1.4
[94]	Females OVX 4 weeks old	MCF-7	Genistein	120	11 weeks	Same prolif.	0.44
			Genistein	240	11 weeks	Same prolif.	0.51
[95]	Females Balb/c 4 weeks old OVX	MCF-7	Genistein	20	22 weeks	No effect	0.39
				40		Proliferation	1.2
				80		Proliferation	2.8
				160		Proliferation	3.4
[96]	Females Balb/c 4 weeks old OVX	MCF-7	Genistein ± TAM	160	32 weeks	Proliferation ± TAM	3.9 5.0
[97]	Females OVX 4 weeks old	MCF-7	Soy meal + Isofl	212 ^b^	10 weeks	No effect	3.7 ^b^
			Molasse + Isofl	215 ^b^		Proliferation	4.5 ^b^
			Novasoy	213 ^b^		Proliferation	5.5 ^b^
			Mixed Isofl	184 ^b^		Proliferation	7.5 ^b^
			Genistin	133 ^b^		Proliferation	2.0 ^b^
[98]	Females SCID 5–8 weeks old	MCF-7	Soy extract	100	8 weeks	Inhibition	Unknown
			Soy extract	500		Inhibition	Unknown
			Soy Isofl	56		Inhibition	Unknown
			Soy Isofl	280		Inhibition	Unknown
[99]	Females Balb/c 4 weeks old OVX	MCF-7	Daidzein	20	22 weeks	No effect	0.3
				40		No effect	0.6
				80		Proliferation	0.9
				160		Proliferation	1.8
[100]	Females Balb/c 4 weeks old OVX	MCF-7	Genistein ± E2	80	18 weeks	Additive prolif.	2.8
[101]	Females OVX 8 weeks old	MCF-7	Isofl glyc. 5 days/week	1.23	5 weeks	No effect	0.2 ^c^
				2.56		No effect	0.25 ^c^
[55]	Females Balb/c 6 weeks old OVX	T47D TAM-resistant	Genistein ± TAM	32	14 weeks	Proliferation ++	1.0
			Daidzein ± TAM	23		Proliferation ++	0.3
			Soy diet	Unknown	10 weeks	Proliferation	Unknown
[102]	Females Balb/c 4 weeks old OVX	MCF-7	Genistein ± letrozole	40	20 weeks − L 32 weeks + L	Proliferation	1.5–1
				80		Proliferation	2.8–1.5
				160		Proliferation	4.8–6
[103]	Females Balb/c 4 weeks old OVX	MCF-7	Oral genistein	80	23 weeks	Proliferation	2.8 ^c^
			Soy flour	64		Proliferation	2.0 ^c^
[50]	Females Balb/c 4 weeks old OVX	MCF-7 E10	Genistein	40	25 weeks	No effect	1.5 ^c^
			Genistein	80		No effect	2.8 ^c^
			S-equol	40		No effect	Unknown
			S-equol	80		No effect	Unknown
			SE5-OH	40		No effect	Unknown
			SE5-OH	80		No effect	Unknown
[104]	Females Balb/c 4 weeks old OVX	MCF-7	Genistein + TAM	40	35 weeks	No effect	2.6 ^c^
			Gen + TAM + E2	20		Proliferation	1.4 ^c^
			Gen + TAM + E2	40		Proliferation	2.6 ^c^
			Gen + TAM + E2	80		Low prolif	3.3 ^c^
[105]	Females Balb/c 4 weeks old OVX	MCF-7	Genistein	120	15w+/9w−/7w+	+ GEN: prolif. − GEN: regression	2.1
			Genistein	80	23w+/11w−	+ GEN: prolif. − GEN: no regression	1.8
			Soy protein	30	32w+/8w−	+ GEN: prolif. − GEN: no regression	0.8
			E2	Menopausal E2 serum	19w+/6w−/6w+	+ E2: prolif. − E2: regression	None
[106]	Females Balb/c 4 weeks old	MCF-7	Genistein	16 160	4 weeks	Prolif. reduced	0.6 ^c^ 6 ^c^
		MCF-7/ERβ				Prolif. reduced	
		MBA-MD-231/ERβ				No effect	
[107]	Females Balb/c 4 weeks old	MCF-7	Genistein	80	18 weeks	Proliferation	2.8 ^c^
			GEN + Eq	80 + 40		Proliferation	2.8 ^c^ + 2.2 ^c^
			GEN + Eq	80 + 80		Proliferation	2.8 ^c^ + 4.5 ^c^
			GEN + Eq	80 + 160		Proliferation	2.8 ^c^ + 6.8 ^c^
			Eq	40	13 weeks	No effect	2.24
			Eq	160		No effect	6.84

^a^ These doses were calculated assuming a daily food consumption of 4 g and a weight of 25 g for Balb/c mice and of 20 g for other athymic mice; ^b^ from [108]; and ^c^ estimated from other studies. GEN: genistein; OVX: ovariectomized; prolif.: proliferation; and TAM: tamoxifen.

**Table 5:** Summary of the effect of oral isoflavones on athymic nude mice implanted with estrogen-independent human BC cells.

References	Animals	Cell Lines Implanted	Treatment	Dosages ^a^ mg/kg bw/day	Test Duration	Effects	Concentrations in Plasma (µM)
[109]	Females OVX 5 weeks old	MDA-MB-231	Genistein	150	9 weeks	Proliferation	1.6
					1 + 9 weeks	No effect	1.6
[101]	Females OVX 8 weeks old	MDA-MB-231	Isofl glycosides 5 days/week	1.23	5 weeks	No effect	0.2 ^b^
				2.56		No effect	0.25 ^b^
[110]	Females Balb/c 4 weeks old OVX	MDA-MB-231	Genistein 3 weeks	150	13 weeks	Inhibition	2.1 ^b^
			Soy extract 3 weeks			Inhibition	Unknown
[111]	Females 5 weeks old	MDA-MB-231	Genistein	50	5 weeks	Inhibition	1.4 ^b^
[103]	Females Balb/c 4 weeks old	MDA-MB-231	Genistein	16	4 weeks	No effect	0.6 ^b^

^a^ These doses were calculated assuming a daily food consumption of 4 g and a weight of 25 g for Balb/c mice and of 20 g for other athymic mice; ^b^ estimated from other studies.

**Table 6:** Summary of the studies retained by the EFSA panel in 2015 [139].

References	End Point ^1^	Substance Tested ^2^	Isoflavones (mg/day) ^3^	Number of Subjects Treated/Control	Duration (Months)	Effect
[140]	MD	SI	37	65/62	12	No
[141]	MD	SI	100	40/32	10	No
[142]	MD	SI	99	100/70	12	No
[143]	MD	SI	80	120/115/123 ^4^	24	No
[144]	MD	Genistein	54	198/191	12	No
[145]	MD	Genistein	54	30/30	12	No
[146]	MD	CI	43.5	102/103	12	No
[147]	MD	CI	40	39/38	12	No
[148]	AC	SI	235	49/49	6	No
[149]	AC	SI	60	26/25	3	No

^1^ MD: mammary density; AC: atypical cytology; ^2^ SI: soy Isofl; CI: clover Isofl; ^3^ in aglycone equivalents; ^4^ high-dose/low-dose/control.

**Table 7:** List of the main confounding factors that should be considered for adjustment in population studies on the effects of soy and isoflavones on breast cancer risk.

N°	Family Factors	N°	Endocrine Factors	N°	Environmental Factors
1	History of benign breast disease	6	Age at menarche	14	Asian, African, Western, etc., countries
2	History of breast cancer	7	Age at menopause	15	Fruit and vegetable consumption
3	Personal history of benign breast disease	8	Age at first delivery	16	Dietary pattern: Western, Asian, etc.
4	Personal history of breast cancer	9	Parity	17	Cruciferous consumption
5	Ethnicity	10	Use of hormonal contraception	18	Green tea consumption
		11	Use of hormones for menopause	19	Alcohol consumption
		12	Time since menopause	20	Physical activity
		13	BMI at menopause	21	Smoking status
				22	Endocrine disruptor exposure
				23	Socioeconomic situation
				24	Education
				25	Cooking process for soy food

All studies on the association between soy and/or isoflavones and breast cancers and cited in this review were adjusted for women’s age and energy intake.

**Table 8:** Summary of case–control studies on soy and/or isoflavones and breast cancer risk.

Ref	Number of Subjects	Soy Exposure	Doses of Isoflavones	Results	Effect Recorded	Confounding Factors Missed	Reliability
**Asian**							
[174]	109/207	Soy food 16.8 to 55.1 g/d Soy protein 1.4 to 3.5 g/d	No estimation	After adjustment Q1 vs. Q5 OR (95% CI) | *p* Soy protein 0.47 (0.24–0.98) | 0.08 Total soy foods 0.39 (0.19–0.77) | 0.02	Less meat and more PUFAs, β-carotene, and soy proteins ↓ BC risk pre-menopause but not post-menopause.	1, 3, 12, 15, 17, 18, 19, 20, 21, 22, 23, 25	Low
[175]	200/420	Quantitative FFQ Soy foods (g/d) T1: <20.3 T2: 20.3–54.9 T3: ≥55	No estimation	T1 vs. T3 OR (95% CI) | *p* Soy protein (g) 0.4 (0.2–0.8) | 0.01 Soy/total protein 0.3 (0.1–0.6) | 0.001 Total soy products (g) 0.4 (0.2–0.9) | 0.01	Soy foods and soy protein ↓ BC risk pre-menopause.	1, 3, 11, 12, 13, 15, 17, 18, 19, 20, 22, 25	Low
[176]	1186/23,163 607 cases Pre-menop. 445 cases Post-menop.	Bean curd T1: ≤3/month T2: 1–2/week T3: ≥3/week	Not applicable	Bean curd T1 vs. T3 OR (95% CI) pre-menopausal women 0.78 (0.6–1.00) post-menopausal women 0.96 (0.70–1.31)	No significant effect of soy or other vegetables. Ham and sausages ↑ BC risk post-menopause.	1, 10, 11, 12, 17, 18, 22, 24, 25	Moderate
[177]	834/834	Soy protein 1.6–12.4 g/d	Not applicable	RR (95% CI) Soy proteins (g) 1.0 (0.7–1.4) Soy protein (%) 1.0 (0.7–1.5)	No effect of soy proteins on BC risk. Fat ↑ BC risk. Fibers, green vegetables, carotenoids, and vit C $ BC risk.	1, 7, 8, 12, 17, 18, 20, 23, 25	Moderate
[178]	1459/1556	Total soy food (g/wk) mean ± SD 947.8 ± 889.0 Median (25th, 75th) Percentile 654.5 (350.0, 1249.5)	Isofl (mg/wk) mean ± SD 286.3 ± 276.5 Median (25th, 75th) Percentile 232.4 (130.9, 373.1)	Soy food intake D1 vs. D10 OR (95% CI) | *p* All subjects 0.66 (0.46–0.95) | 0.28 Subjects on same diet 0.46 (0.28–0.75) | 0.02	No clear dose–effect relationship between soy intake and BC. Soy food may ↓ the risk of BC, especially the ER+ and PR+ forms.	12, 17, 18, 22, 23, 25	Reasonable
[179]	1459/1556	Tofu (g/d,): Q1: <0.44 Q2: 0.44–0.88 Q3: 0.88–1.32 Q4: 1.32–2.2 Q5: >2.2 All soy food (g/d) Q1: <2.20 Q2: 2.2–4.4 Q3: 4.41–6.6 Q4: 6.61–11.0 Q5: >11.0	Not applicable	Soy food intake in youth and BC risk as adult OR (95% CI) | *p* Q1: 1.0 as reference, Q2: 0.75 (0.60–0.93) Q3: 0.69 (0.55–0.87) Q4: 0.69 (0.55–0.86) Q5: 0.51 (0.40–0.65) | <0.001.	Soy food intake in youth ↓ BC risk.	1, 10, 12, 17, 18, 21, 23, 25	Moderate
[180]	2385/19,013	Q1: 1–3 t/month Q2: 1–2 t/wk Q3: 3–4 t/wk Q4: ≥5 t/wk	Not applicable	Not significant after adjustment	No significant effect of soy intake on BC risk.	1, 3, 10, 12, 18, 22, 23, 24, 25	Moderate
[181]	357/357	All women (g/d) T1: <6.96 T2: 6.96–12.21 T3: ≥12.22 Pre-menopause T1: <6.89 T2: 6.89–11.85 T3: ≥11.86 Post-menopause T1: <7.28 T2: 7.28–13.18 T3: ≥13.19	Estimation from database	Not significant after adjustment	No significant effect of soy protein on BC risk.	1, 4, 12, 15, 16, 17, 18, 19, 21, 22, 23, 25	Low
[182]	219/250	FFQ for soy foods (g/wk) Q1: ≤114 Q2: 114–191 Q3: 192–341 Q4: >341	Not applicable	**Women ≤ 40** Not significant **Women > 40** Not significant	Fat ↑ BC risk. Vitamin linked to control status. Beef and pork ↑ BC risk. Soy tends to ↑ BC risk, not significant.	1, 4, 12, 17, 19, 21, 23, 25	Low
[183]	155/1070	Soy food intake Q1: ≤121 t/y Q2: 122–219 t/y Q3: 220–368 t/y Q4: ≥369 t/y	Not applicable	OR (95% CI) | *p* Benign fibroadenoma Q2, Q3, Q4 0.8 (0.5–1.3) 0.7 (0.4–1.2) 0.6 (0.3–1.0) | 0.04 Cancerous fibroadenoma Not significant	No significant effect of soy intake on BC risk.	1, 5, 11, 12, 17, 22, 23, 25	Moderate
[18]	1052/23,163	Soy food intake (g/100 kcal) Tertile medians Pre-menopause T1: 17.2 T2: 29.7 T3: 47.9 Post-menopause T1: 20.1 T2: 35.3 T3: 56.5	Estimation from the USDA Database	After adjustment Tofu pre-menopause T2, T3 OR (95% CI) | *p* 0.44 (0.22–0.90) 0.49 (0.25–0.95) |0.03 Fried tofu post-menopause 1.95 (0.98–3.86) 2.28 (1.15–4.51) | 0.02	Tofu and estimated Isofl ↓ BC risk in pre-menopause and fried tofu ↑ BC risk in post-menopause.	1, 3, 10, 11, 12, 17, 18, 22, 23, 24, 25	Low
[184]	378/1070	Soy food intake via specific FFQ Q1: ≤2.6 t/wk Q2: 2.6–4.4 t/wk Q3: 4.4–7.7 t/wk Q4: ≥7.7 t/wk	Not applicable	Not significant after adjustment	Fruits and vegetables ↓ BC risk. No effect of soy.	1, 12, 18, 22, 23, 25	Reasonable
[185]	359/708	Soybeans (g/d) Q1: <0.31 Q2: 0.31–1.62 Q3: 1.62–3.03 Q4: >3.03 Soybean paste (g/d) Q1: <1.82 Q2: 1.82–5.38 Q3: 5.38–9.24 Q4: >9.24	Not applicable	OR (95% CI) | *p* Soybeans Q1 vs. Q4 0.67 (0.45–0.91) | 0.02 Soybean paste Q1 vs. Q3 0.69 (0.39–1.09) | 0.04	No association of total fruits, vegetables, or soy food and BC risk. Grapes, tomatoes, and cooked soybeans ↓ BC risk.	1, 3, 12, 18, 22, 25	Reasonable
[186]	678/3390	Soy food (g/d) T1: 1.1–27.4 T2: 27.4–51.2 T3: 51.2–326.3	Not applicable	OR (95% CI) | *p* T1 vs. T3 **ER+** 0.74 (0.58–0.94) | 0.01 **HER2−** 0.78 (0.61–0.99) | 0.04 ER+/PR+/**HER2−** 0.73 (0.54–0.97) | 0.03	Soy intake ↓ BC risk in ER+, HER2− ER+/PR+/HER2− Tumors.	1, 3, 10, 12, 15, 17, 18, 22, 23, 24, 25	Low
[187]	362/362 pre-menopause 235/235 post-menopause 127/127	Soy protein (g/d) Q1: <4.24 Q2: 4.25–6.34 Q3: 6.35–8.09 Q4: 8.10–10.54 Q5: ≥10.55 Tofu intake (g/d) Q1: <7.73 Q2: 7.74–14.39 Q3: 14/4–23.59 Q4: 23.6–49.49 Q5: ≥49.5 Tofu slice (N°) T1: ≤2 t/wk T2: ≤1 t/d T3: >1 t/d	Not applicable	**Pre-menopause** Total soy protein Q1 vs. Q5 OR (95% CI) | *p* 0.39 (0.22–0.93) | 0.03 Total tofu Q1 vs. Q5 0.23 (0.11–0.48) | <0.01 Tofu slices T1 vs. T3 0.26 (0.13–0.55) | <0.01 **Post-menopause** Total soy protein 0.2 (0.06–0.88) | 0.16	Soy and tofu ↓ BC risk pre- menopause, not post-menopause.	1, 3, 12, 17, 18, 22, 23, 25	Low
[188]	8 studies: 1: 109/207 2: 1459/1556 3: 501/594 4: 179/209,354 5: 1052/23,163 6: 219/250 7: 378/1070 8: 359/708	Soy recorded from FFQ 165 foods and beverages expressed in eq tofu	Estimated from FFQ and database Low: ≤5 mg/d Moderate: ≈10 mg/d High: ≥20 mg/d stratification for 10.6 mg/d for 1000 kcal intake	RR (95% CI) Moderate vs. low All studies 0.88 (0.78–0.98) High vs. low All studies 0.71 (0.60–0.85)	Soy ↓ BC risk significantly in Asia. No effect in Western population. No *p*-values.	1, 3, 8, 10, 11, 12, 15, 17, 20, 22, 23, 25	Low
[189]	438/438	Soy protein (g/d) Q1: <0.93 Q2: 0.93–2.33 Q3: 2.33–4.66 Q4: >4.66	Estimated (mg/d) Q1: <3.26 Q2: 3.26–8.07 Q3: 8.07–16.89 Q4: >16.89	ORs (95% CI) | *p* **Soy protein** Q1 vs. Q4 0.62 (0.40–0.96) | <0.001 **Soy Isofl** Q1 vs. Q4 0.54 (0.34–0.84) | <0.001	Soy and estimated Isofl intake ↓ BC risk pre-menopause. Less clear post-menopause.	1, 12, 17, 18, 22, 25	Reasonable
[190]	403/662	Semi-quantitative FFQ for Singapore food validated by 24 h dietary inquiries	Estimated Isofl (mg/1000 kcal) G1: <10.6 G2: ≥10.6	No significant effect of estimated Isofl independently to the genotype identified	Isofl slightly ↓ BC risk with MDM2 SNP309 GG genotype. No effect on other genotypes.	1, 3, 4, 10, 11, 12, 14, 15, 16, 17, 18, 19, 20, 21, 22, 23, 25	Low
[191]	306/662	6 soy foods with tofu and miso 3 periods: 10 to 12 y old 20 y old 10 to 15 y prior to the study	Estimated from Japanese database (mg/d) Q1: <18.76 Q2: 18.76–28.81 Q3: 28.81–43.75 Q4: >43.75	Fermented drinks OR (95% CI) | *p* 0.65 (0.42–1.00) | 0.048 Soy food intake Q1 vs. Q3 Q4 0.53 (0.35–0.81) 0.48 (0.31–0.73) |0.0002	*Lactobacillus*-rich drinks in youth and soy associated with ↓ BC risk. Possible interaction.	1, 8, 11, 12, 15, 16, 17, 18, 19, 22, 23, 25	Low
[192]	183/192	Soy protein (g/d) Q1: <2.12 Q2: 2.12–7.03 Q3: 7.03–13.02 Q4: >13.03	Estimated Isofl (mg/d) Q1: <7.56 Q2: 7.56–17.31 Q3: 17.32–28.82 Q4: >28.83	Q1 vs. Q4 OR (95% CI) Isofl intake 0.42 (0.22–0.80) soy protein 0.46 (0.24–0.88) Post-menopause 0.57 (0.29–0.83) 0.50 (0.38–0.95) ER+/PR+ Isofl 0.47 (0.19–0.85) ER+/PR+ soy protein 0.63 (0.45–0.97)	Estimated Isofl and soy proteins ↓ overall BC risk. Also ↓ of ER+/PR+ BC risk post-menopause. No *p*-value.	1, 8, 12, 16, 17, 18, 22, 25	Low
[193]	233/236	Tofu Soybean milk T1: 9 t/month T2: 4 t/wk T3: 1 t/d	Estimated (mg/d) Vegetarians 25.9 ± 25.6 Non-vegetarians 18.1 ± 15.6 (*p* < 0.001) BC: 17.2 ± 16 Cont: 26.3 ± 24.7 (*p* < 0.001)	OR (95% CI) | *p* Fruit/vegetables/soy diet 1 t/d 1.01 (0.82–1.26) | 0.98 Isofl intake (mg/d) >22 vs. <22 0.37 (0.24–0.60) |<0.001	Meat and processed meat ↑ BC risk. Vegetarian and soy food ↓ BC risk. Estimated Isofl ↓ BC risk.	1, 3, 4, 5, 7, 8, 12, 14, 17, 18, 22, 23, 25	Low
[194]	818/935	FFQ, 149 Chinese food items (g/d) Q1: 0–3.3 Q2: 3.4–28.6 Q3: 28.6–57.1 Q4: >57.1	Not applicable	OR (95% CI) | *p* Vegetable–fruit–soy diet pattern Q1 vs. Q4 post-menopause 0.57 (0.41, 0.80) |< 0.001 ER− subtypes 0.63 (0.37–0.94) | 0.003 ER−/PR− subtypes 0.64 (0.41, 0.93) | 0.012 Soy (g/d), Q2, Q3, Q4 0.68 (0.51, 0.91) 0.65 (0.50, 0.86) 0.52 (0.39, 0.69) |< 0.001	Vegetable–fruit–soy diet ↓ BC risk post-menopause in ER−BC subtypes. No effect in ER+ BC subtypes.	1, 12, 17, 18, 22, 25	Reasonable
**Multiethnic**							
[195]	597/966	Tofu (times/year) Q1: ≤12 Q2: 13–42 Q3: 43–54 Q4: >55	Not applicable	OR (95% CI) 0.85 (0.74–0.99)	Tofu slightly ↓ BC risk No *p*-value.	1, 2, 3, 8, 10, 11, 12, 13, 15, 16, 17, 18, 19, 20, 21, 22, 23, 24, 25	Low
[196]	1326/1657	Recall from the previous year Tofu ≥ 1/m vs. 0 Miso ≥ 1/m vs. 0	Estimated from specific database Total Isofl (mg/d) Q1: <1.048 Q2: 1.048–1.647 Q3: 1.648–2.774 Q4: ≥2.775	OR (95% CI) Q1 vs. Q4 Total Isofl/1000 µg/d 0.99 (0.98–1.01) Total lignans/100 µg/d 1.08 (0.98–1.14) Total phyto/100 µg/d 0.99 (0.98–1.01)	No effect of Isofl but exposure is low No *p*-value.	1, 12, 14, 15, 17, 18, 19, 21, 22, 25	Moderate
[197]	501/594	Tofu in youth Q1: <1 t/month Q2: 1–3 t/month Q3: 1–3 t/wk Q4: >4 t/wk	Isofl in adults (mg/1000 kcal) Q1: ≤1.79 Q2: >179–6.24 Q3: >6.24–12.68 Q4: >12.68	OR (95% CI) | *p* **Tofu in youth** Q1 vs. Q3 0.65 (0.42–0.92) |0.04 **Isofl in adults** 0.61 (0.39–0.97) | 0.04 **Q4 Tofu in youth and Q4 Isofl in adults** 0.65 (0.43–0.97) | 0.03	Moderate tofu in youth ↓ BC risk in adults. Isofl ↓ BC risk in adults. High tofu in youth and high Isofl in adults ↓ BC risk.	1, 3, 4, 8, 10, 12, 17, 18, 22, 23, 25	Low
[198]	501/594	Soy food	Estimated from FFQ and USA database mg/1000 kcal Q1: ≤ 1.79 Q2: >1.79–6.24 Q3: >6.24–12.68 Q4: >12.68	OR (95% CI) Soy intake youth/adult reference is low/low **No green tea** Low/high 0.81 (0.52–1.27) High/high 0.40 (0.24–0.66) **Green tea** Low/low 0.45 (0.26–0.78) Low/high 0.52 (0.31–0.85) High/high 0.41 (0.25–0.65)	Soy ↓ BC risk. Intake in youth ↓ BC risk. Effect amplified by green tea intake No *p*-value.	1, 3, 7, 10, 12, 15, 16, 17, 22, 23, 25	Low
[199]	850/850	Recall from the previous year Tofu ≥ 1/m vs. 0 Miso ≥ 1/m vs. 0	Estimated from database; median Isofl (mg/d) Q1: 8.7 Q2: 23.1 Q3: 33.8 Q4: 45.7 Q5: 71.3	All subjects OR (95% CI) | *p* 0.69 (0.44–1.09) 0.54 (0.31–0.94) 0.45 (0.26–0.77) 0.34 (0.19–0.62) 0.43 (0.24–0.76) | 0.01	Estimated Isofl ↓ BC risk.	1, 3, 11, 12, 14, 17, 18, 22, 23, 24, 25	Low
**Western**							
[200]	820/1548	No soy exposure but FFQ	Estimated from the US database median (mg/d) Q1: 0.01 Q2: 0.2 Q3: 0.2 Q4: 0.3 Q5: 0.8	Isofl Q1 vs. Q5 OR (95% CI) | *p* 1.07 (0.97–1.18) | 0.17	No effect of estimated Isofl on BC risk.	1, 2, 3, 4, 5, 10, 11, 12, 14, 16, 17, 18, 20, 21, 22, 23, 24, 25	Low
[201]	239/475	Only through Asian diet Western foods not considered	Estimated from FFQ and database Total Isofl mg/1826 kcal Q1: ≤0.125 Q2: 0.126–0.253 Q3: 0.254–0.469 Q4: ≥0.470	Adjust OR (95% CI) |*p* Tot Isofl, Q2, Q3, Q4 1.39 (0.87–2.22) 1.29 (0.79–2.12) 0.65 (0.38–1.13) | 0.15 Tot lignans, Q2, Q3, Q4 0.72 (0.43–1.20) 0.81 (0.49–1.34) 0.69 (0.40–1.18) | 0.27	No effect of estimated Isofl on BC risk.	1, 4, 14, 17, 18, 22, 25	Moderate
[202]	278/666	Validated FFQ [203]	Estimated from 4 databases of European foods Median (mg/d) Cases: 0.279 Control: 0.289	Adjust OR (95% CI) |*p* Genistein Q3, Q4 0.68 (0.44–1.05), 0.47 (0.29–0.74) | 0.002 Daid + Gen Q3, Q4 0.63 (0.41–0.96), 0.56 (0.36–0.87) | 0.005	After adjustment, Genistein and Gen+Daid tend to ↓ BC risk but not total isoflavonoids. No quartile value.	1, 3, 4, 5, 8, 10, 11, 12, 14, 15, 17, 18, 22, 23, 25	Low
[204]	3063/3430	Soy-based diet sold in Canada	Estimated from FFQ and database (mg/d) Q1: 0–0.082 Q2: 0.083–0.154 Q3: 0.155–0.344 Q4: 0.345–1.236 Q5: 1.237–158.983	OR (95% CI) All women Lignans Q5 vs. Q1 0.81 (0.65, 0.99) Overweight women pre-menopause all phyto Q5 vs. Q1 0.51 (0.30, 0.87) lignans Q5 vs. Q1 0.70 (0.53-0.93)	Total phyto and lignan intake ↓ BC risk in overweight women only. No effect of Isofl. Low exposure. No *p*-value.	1, 12, 14, 15, 17, 18, 22, 25	Moderate
[205]	2438/3370	Validated FFQ	Estimation of Isofl lignans and total phyto from FFQ, databases Isofl (mg/d) Adults T1: <0.122 T2: 0.123-0.496 T3: ≥0.497 Adolescents T1: <0.01 T2: 0.011–0.02 T3: ≥0.02	OR (95% CI) | *p* Post-menopause, **ER+/PR+ subgroup** Total phyto 0.79 (0.65–0.96) | **ER−/PR− subgroup** 1.38 (1.05–1.81) | 0.01 **ER−/PR− subgroup** Pre-menopause 1.65 (1.06–2.57) | 0.04 Isofl post-menopause 1.50 (1.05–2.15) | 0.04	Total phyto ↓ BC risk in ER+/PR+ subgroup post-menopause. No significant effect of Isofl intake in youth. Isofl ↑ BC risk in ER−PR− subgroup. Exposure is low.	1, 4, 9, 10, 12, 14, 15, 17, 18, 19, 20, 21, 22, 23, 25	Low

**Table 9:** Summary of cohort studies on soy and/or isoflavones and breast cancer risk.

Ref	Number of Subjects	Soy Exposure	Doses of Isoflavones	Results	Effect Recorded	Confounding Factors Missed	Reliability
**Asia**							
[168]	825/47,614	Soy foods (g/d) Q1: 30.1 ± 10.4 Q2: 56.4 ± 6.8 Q3: 83.3 ± 9.4 Q4: 169 ± 107	Estimated from Japanese database	QI vs. Q4 HR (95% CI) | *p* Nonlocalized BC fermented soy 0.53 (0.28–0.99) | 0.02	Fermented soy food associated with ↓ nonlocalized BC.	3, 4, 12, 15, 17, 18, 20, 22, 23, 24, 25	Low
[210]	1460 BC patients	FFQ recording soy products	Estimated from Hong Kong soy food database [211] <3–12.5 mg/d	No significant effect	Tendency to ↑ BC risk.	6, 8, 9, 12, 15, 17, 18, 22, 24, 25	Moderate
[212]	2289/300,000	FFQ repeated Rarely Monthly 1–3 times/wk ≥4 times/wk	Estimation (mg/d) Q1: 4.5 Q2: 7.2 Q3: 14.4 Q4: 19.1	No effect of soy potential effect of Isofl (/10 mg)	No significant effect of soy potential effect of Isofl.	1, 3, 4, 8, 11, 12, 17, 18, 21, 22, 25	Moderate
[213]	1034/70,578	Validated FFQ for soy proteins adulthood T1: 4.5 g/d T2: 8.2 g/d T3: 13.5 g/d Soy protein in youth T1: 2.6 g/d T2: 6.2 g/d T3: 12.5 g/d	Estimation from FFQ and database of Shanghai soy foods Adults (mg/d) T1: 14.5 T2: 27.8 T3: 46.7 Adolescents (mg/d) T1: 6.4 T2: 16.0 T3: 34.1	HR (95% CI) | *p* T1 vs. T3 all adults ER+/PR+ 0.75 (0.58–0.98) | 0.03 Pre-menopause 0.46 (0.22–0.97) | 0.04 Post-menopause 0.72 (0.53–0.96) | 0.02 High for all ages Pre-menopause 0.53 (0.32–0.88) Low in youth and high in adulthood Post-menopause 0.63 (0.43–0.91)	Preventive effect in women only in some cases. Limited Significance.	3, 4, 9, 10, 11, 17, 18, 19, 21, 22, 25	Low
[214]	172/15,607	Validated FFQ of 169 items; mean ± SD (g/d) Q1: 40.2 ± 17.5) Q2: 71.9 ± 6.9 Q3: 97.9 ± 8.8 Q4: 162.8 ± 64.6	Estimation from FFQ and database Isofl mean ± SD (mg/d) Q1: 19.9 ± 10.0 Q2: 33.9 ± 7.8 Q3: 44.7 ± 10.1 Q4: 67.4 ± 26.3	HR (95% CI) | *p* Q1 vs. Q4 Post-menopause 0.63 (0.39–1.01) | 0.023 0.52 (0.32–0.85) | 0.046	↓ in BC risk linked to soy and Isofl in post-menopausal women. Low number of cases.	1, 2, 3, 4, 10, 12, 15, 16, 17, 18, 22, 23, 25	Low
[215]	79/616	validated FFQ with Soy proteins (g/d) Q1: <2.12 Q2: 2.12–7.03 Q3: 7.03–13.03 Q4: >13.03	Estimation from databases for industrial soy foods from China	HR (95% CI); Soy: Q1 vs. Q4 All: 0.71 (0.52–0.98) er + bc: 0.59 (0.40–0.93) Isofl Q1 vs. Q4 All: 0.62 (1.42–0.90) er + bc: 0.59 (0.40–0.93) er−bc: 0.78 (0.47–0.98)	Soy food and estimated Isofl linked to long-term survival in ER+ BC patients. No *p*-value.	6, 8, 9, 10, 11, 12, 13, 14, 15, 16, 17, 18, 20, 22, 23, 25	Low
[216]	629/34,028	FFQ for Asian foods. Recall. Two patterns: -Vegetable–fruit–soy diet -Meat–dim sum diet	Estimation from database (mg/d) meat–dim sum Q1: 9.68; Q2: 12.55; Q3: 15.07; Q4: 20.33 in vegetable–fruit–soy Q1: 7.45; Q2: 12.00 Q3: 16.28; Q4: 24.58	HR (95% CI) | *p* Q4 vs. Q1 Vegetable–fruit–soy diet All women 0.82 (0.63–1.05) | 0.03 Post-menopause 0.70 (0.51–0.95) | 0.01 After ≥5 y follow-up 0.57 (0.36–0.88) |<0.01	Vegetable–fruit–soy diet prevents BC risk. No effect of soy or estimated Isofl alone.	3, 4, 8, 10, 12, 17, 22, 23, 25	Moderate
[217]	5042 BC survivors 444 deaths 534 recurrences	FFQ for Asian foods Recall Soy proteins (g/d) Q1: ≤5.31 Q2: 5.32–9.45 Q3: 9.46–15.31 Q4: ≥15.31	Estimated from FFQ and database of Chinese foods (mg/d) Q1: ≤20.00 Q2: 20.01–36.50 Q3: 36.51–62.68 Q4: ≥62.68	HR (95% CI) Q1 vs. Q4 soy and tot mortality 0.67 (0.51–0.88) soy and recurrence 0.66 (0.52–0.84) Isofl effect not significant	No effect of estimated Isofl Soy slightly ↓ BC risk of mortality and recurrence. No *p*-value.	1, 2, 3, 6, 8, 9, 10, 12, 15, 18, 19, 21, 22, 25	Low
[218]	592/73,223	Validated FFQ Soy (g/d), recall for adolescence Q1: ≤4.87 Q2: 4.88–7.11 Q3: 7.12–9.48 Q4: 9.49–12.82 Q5: ≥12.82 g/d	Estimated from FFQ Q1: ≤15.93 mg/d Q2: 15.94–23.88 mg/d Q3: 23.89–32.43 mg/d Q4: 32.44–44.23 mg/d Q5: ≥44.24 mg/d	RR (95% CI) | *p* Q1 vs. Q5 Pre-menopause soy 0.41 (0.25–0.70) | <0.01 Isofl 44 (0.26–0.73) | <0.01 Post-menopause soy in youth 0.57 (0.34–0.97) | 0.061 Isofl in youth 1.38 (1.00–1.91) | 0.038	Adult soy or Isofl ↓ BC risk at pre-menopause. Soy in youth ↓ BC risk. Isofl ↑ BC risk post-menopause.	3, 4, 6, 8, 9, 10, 11, 12, 14, 16, 17, 18, 19, 21, 22, 23, 25	Low
[219]	629/34,028	FFQ on 7 soy food in Singapore expressed in equivalent tofu	Estimation from FFQ and database for Asian foods G1 = low: <10.6 mg Isofl/1000 Kcal), G2 = high: > 10.6 mg Isofl/1000 Kcal)	RR (95% CI) high vs. low 0.82 (0.70–0.97). Post-menopause 0.74 (0.61–0.90) Post-menopause above median BMI 0.67 (0.51–0.88)	Estimated Isofl slightly ↓ BC risk post-menopause. No effect pre-menopause. No *p*-value.	3, 4, 8, 10, 11, 12, 15, 18, 20, 22, 23, 25	Low
[220]	145/30,454	FFQ on Japanese soy foods T1: ≤2 times/wk T2: 3–4 times/wk T3: almost daily	Not applicable	No significant effect	No link between soy food intake and BC risk.	3, 4, 12, 16, 17, 18, 19, 22, 23, 24, 25	Low
[221]	179/209,354	Miso soup <1 time/d; 1 cup/d 2 cups/d; ≥3 cups/d Soy foods <2 times/wk 3–4 times/wk Almost every day	Isofl (mg/d) Q1: 6.9 ± 2.6 Q2: 13.0 ± 2.1 Q3: 20.0 ± 2.1 Q4: 25.3 ± 2.2	RR (95% CI); Q1 vs. Q4 post-menopause 0.32 (0.14–0.71)	Estimated Isofl ↓ BC risk. No effect of miso or soy foods. No effect at pre-menopause. No *p*-value.	3, 4, 10, 11, 13, 17, 18, 22, 23, 25	Low
**Multiethnic**							
[222]	427/19,560	FFQ for miso and tofu. (time/wk) T1: ≤1 T2: 2–4 T3: ≥5	Not applicable	No significant effect	No association of soy with breast cancer.	Adjusted for radiation exposure	Low
[223]	Soy: 538,337 Isofl: 842,964	FFQ for soy food and/or soy protein	Estimated from FFQ based on soy products	Not significant	No significant effect.	3, 4, 6, 7, 8, 9, 10, 11, 12, 15, 17, 18, 22, 23, 24	Low
[208]	11,169/648,913	Soy intake mainly assessed by FFQ	Estimated from industrial soy foods High vs. low or moderate vs. low	Limited significance	High soy intake may ↓ BC risk but not Isofl.	3, 4, 5, 6, 8, 9, 10, 12, 14, 15, 16, 17, 18, 22, 23, 25	Low
[224]	3842 Multiethnic cohort study, USA	Specific FFQ Validated 0.0 to 8.1 g soy/d	Estimated from FFQ and specific database T1: 0–<4.3 mg/d T2: 4.3–<10.4 mg/d T3: ≥10.4 mg/d	No significant effect	No link between soy and BC.	1, 2, 3, 6, 7, 8, 9, 10, 11, 12, 15, 16, 17, 18, 19, 20, 22, 23, 24, 25	Low
[225]	9514 BC survivors 1171 deaths 1348 recurrence	Validated FFQ of Shanghai soy foods, with a long time from event to inquiry	Estimation from FFQ and databases for USA or Shanghai Foods (mg/d) SBCSS: 45.9 ± 38.3 WHEL: 2.6 ± 7.9 LACE: 4.1 ± 11.9	≥10 mg Isofl/d HR (95% CI) BC recurrence 0.75 (0.61–0.92)	Tendency to prevent BC recurrence with Isofl > 10 mg/d. No *p* value	1, 2, 3, 4, 6, 8, 10, 12, 14, 15, 18, 19, 22, 23, 25	Low
[226]	3088 BC survivors Q1: 1095 Q2: 1094 Q3: 410 Q4: 137	Recorded with AFFQ, no data used	Estimated from FFQ and US database for Isofl (mg/d) Median (min–max) Q1: 0 (0–0.7) Q2: 0.3 (0.7–1.01) Q3: 4.8 (1.01–16.33) Q4: 26.7 (16.33–86.9)	No significant effect	No effect of Isofl on BC risk. Isofl linked to ↓ mortality.	1, 2, 3, 4, 6, 8, 9, 10, 12, 13, 14, 15, 16, 17, 18, 19, 20, 21, 22, 23, 25	Low
**Western Countries**							
[227]	1057/52,795 women 7th Day Adventist	FFQ validated	Estimated from database 0.3 to 44 mg/d	HR (95% CI) | *p* Isofl and BC risk Substituting soy milk for dairy milk 0.68 (0.54–0.87) | 0.002	When soy ↓, dairy products ↑. Dairy product = confounding factors.	3, 4, 10, 12, 17, 18, 22, 25	Moderate
[228]	3241/76,442	Soy intake low and not considered	Food supplements Isofl content variable T1: Never users T2: Current users T3: Past users	HR (95% CI) | *p* T1: vs. T2, T3; **ER+:** | 0.054 T2: 0.78 (0.60–0.99) T3: 1.03 (0.88–1.22) **ER−:** | 0.0007 T2: 2.01 (1.41–2.86) T3: 0.81 (0.53–1.23) Family history of BC 4.23 (2.21–8.07) | 0.03	↓ risk of ER+ BC ↑ risk of ER− BC. Still significant for ER−PR+ and ER−PR.−	1, 5, 14, 16, 17, 20, 22, 23	Reasonable
[229]	6235 BC survivors	Validated FFQ for soy Inquiry long time before or after BC	Isofl (mg/d) Q1: <0.342 Q2: 0.343–0.674 Q3: 0.675–1.493 Q4: 1.494	HR (95% CI) | *p* Q1 vs. Q4 All-cause mortality 0.79 (0.64–0.97) |0.01 ER−/PR− tumors 0.49 (0.29–0.83) | 0.005 No HRT 0.68 (0.51–0.91) | 0.02	Low doses of Isofl ↓ risk of ER−/PR− tumors.	1, 2, 6, 7, 8, 9, 10, 12, 17, 22, 25	Low
[230]	896 in situ 3873 invasive /84,450	Validated FFQ including tofu, miso, vegetarian meat	Estimation on database of USA foods Q1: 1.7 (0.0–3.2) Q2: 4.8 (3.2–6.7) Q3: 9.1 (6.7–12.9) Q4^-^: 16.0 (12.9–20.3) Q4^+^: 29.6 (20.3–178.7)	No significant effect	No significant effect of large range of Isofl intake on BC risk.	3, 4, 15, 16, 17, 18, 22, 23, 25	Moderate
[231]	1954 BC survivors 282 recurrences	FFQ from [232] FFQ from [172] with Isofl assay in plasmas and low correlation with soy intake; FFQ from [233]	Estimation by FFQ Daidzein (mg/d) Q1: 0.0 Q2: 0.001–0.1495 Q3: 0.1496–9.59654 Q4: ≥9.59655	Estimated daidzein post-menopause: HR (95% CI) | *p* 0.48 (0.21–0.79) | 0.008	Significant ↓ in risk with the highest daidzein intake.	1, 2, 3, 4, 6, 8, 9, 10, 11, 12, 14, 15, 16, 17, 1819, 20, 22, 23, 24, 25	Low
[234]	585/37,643	FFQ on diet used to be consumed the year before; 31% of vegetarians	Estimated from database (mg/d) T1: <10 T2: 10–19.9 T3: ≥20	No significant effect	No link between Isofl or vegetarian diet and BC risk.	1, 2, 12, 17, 18, 20, 22, 23, 24, 25	Moderate
[235]	711/111,526	FFQ based on recall from previous year, no hidden soy	Mean: 1.778 mg/d 20^th^–80th percentile (0.641–2.080 mg/d)	RR and 95% CI Genistein Q1 vs. Q5 1.0 (0.7–1.3) Daidzein Q1 vs. Q5 0.9 (0.7–1.2)	No link between Isofl and BC risk No *p*-value.	3, 4, 10, 11, 12, 17, 18, 21, 22, 23, 24, 25	Low

**Table 10:** Summary of case–control studies on soy and/or isoflavones and breast cancer risk, involving isoflavone measurements in urine samples.

Ref	Number of Subjects	Samples Collected	Isoflavone Measurements	Isoflavone Exposure and Biomarkers	Results	Effect Recorded	Reliability
**Asian**							
[236]	60/60	Overnight urine samples adjusted for creatinine	HPLC-DAD (nmol/mg creatinine)	Soy protein intake (g/d) Cases: 8.7 (5.2, 13.3) Cont: 8.7 (4.6, 15.4) *p* = 0.80 Excretion of total Isofl (mean ± SD) Cases: 13.95 ± 20.76 Control: 19.52 ± 25.36 *p* = 0.04	OR (95% CI) Isofl: 0.50 (0.19–1.31) Gen: 0.70 (0.27–1.84) Daid: 0.54 (0.22–1.32)	No significant effect of Isofl on BC risk	Low
[239]	250/250	Overnight urine samples adjusted for creatinine	LC-Mass (nmol/m creatinine)	Soy protein intake (g/d) Cases: 10.69 ± 0.61 Control: 11.49 ± 0.61 Isofl in urine Cases: 32.32 ± 43.70 Control: 40.50 ± 62.55 *p* < 0.01	**All subjects** OR (95% CI) Isofl: 0.62 (0.39–0.99) Gen: 0.79 (0.41–1.03) Daid: 0.54 (0.34–0.85) **Pre-menopause** Isofl: 0.72 (0.36–1.44) **Post-menopause** Isofl: 0.54 (0.28–1.06)	No effect of Isofl. Potential ↓ of BC risk for Daid and glycitein. No effect of Gen ↓ of BC risk with lignans. Tertiles not defined. No *p*-value.	Low
[240]	117/117	Urine sample collected in time-matched controls and cases	LC-Mass (nmoles/mg creatinine)	Soy protein intake (g/d) Cases: 10.8 ± 0.8 Control: 13.2 ± 0.8 *p* = 0.03 Isofl in urine Median (25th, 75th percentiles) Cases: 18.38 (5.36, 44.91) Cont: 26.41 (8.31, 62.32) *p* = 0.04	OR (95% CI) All population Isofl: 0.46 (0.22–0.95) Tertile not defined	Total Isofl ↓ BC risk when E2 is high, and E1-S and SHBG are low No *p*-value.	Low
**Western**							
[241]	88/268	Overnight urine samples for Genistein and enterolactone	TRFIA (nmol/mol creatinine)	Mean intake Gen ± SD: Cases: 107.7 ± 85.7 Cont: 111.6 ± 82.0 Range (median) T1: 10.2–67.1 (48.4) T2: 67.2–112.2 (87.8) T3: 112.3–523.8 (196.6)	OR (95% CI) | *p* Genistein T1 vs. T3 0.83 (0.46–1.51) | 0.6 Enterolactone T1 vs. T3 1.43 (0.79–2.59) | 0.25	No significant effect of Gen or enterolactone on BC risk.	Low
[238]	114/219	Spot urine samples adjusted for creatinine	7-day dietary diaries LC-MS GC-MS (µmol/mmol creatinine)	Mean ± SD Gen: T1: 6.5 ± 2.8 T2: 7.9 ± 2.8 T3: 12.8 ± 2. Daid: T1: 11.5 ± 3.4 T2: 16.1 ± 3.2 T3: 22.4 ± 3.3	OR (95% CI) | *p* Genistein: 1.16 (0.97–1.39) | 0.097 Daidzein: 1.12 (0.96–1.31) | 0.138 Equol: 1.34 (1.06–1.70) | 0.013	No significant effect of Isofl on BC risk. Equol ↑ BC risk.	Low
[242]	219/891	Spot urine samples adjusted for creatinine	7-day dietary diaries LC-MS GC-MS (μg/mmol creatinine)	Median urine Controls: Gen: 5.71 Daid: 14.82 Equol: 0.011 Cases Gen: 6.47 Daid: 14.63 Equol: 0.011	No significant effect on BC risk, except for equol in ER+ BC OR (95% CI) | *p* 1.07 (1.01–1.12) | 0.013	No significant effect of Isofl on BC risk. Equol ↑ BC risk.	Moderate
[243]	251/462	Urine collection overnight or first thing in the morning Samples collected during recruitment	HPLC-MS (nmoles/mg creatinine	**Daid:** Q1: ≤0.183 Q2: 0.184–0.617 Q3: 0.618–2.535 Q4: ≥2.536 **Gen:** Q1: ≤0.022 Q2: 0.023–0.101 Q3: 0.102–0.646 Q4: ≥ 0.647 **Equol:** Q1: ≤0.001 Q2: 0.002–0.004 Q3: 0.005–0.013 Q4: ≥0.014	OR (95% CI) | *p* **Japanese–American** Daidzein Q4 vs. Q1 0.41 (0.19–0.89) | 0.005 Genistein Q4 vs. Q1 0.62 (0.29–1.32) | 0.08 Equol Q4 vs. Q1 1.32 (0.70–2.49) | 0.06 Daid + Gen Q4 vs. Q1 0.51 (0.23–1.13) | 0.008 D + G + Eq Q4 vs. Q1 0.53 (0.24–1.16) |0.003	In Japanese American women, Daid alone or in combination with Gen and Equol ↓ BC risk.	Low

**Table 11:** Summary of case–control studies on soy and/or isoflavones and breast cancer risk, involving isoflavone measurements in plasma or serum samples.

Ref	Number of Subjects	Samples Collected	Isoflavone Measurements	Isoflavones in Samples	Results	Effect Recorded	Reliability
**Asian**							
[246]	500/1002 (196 BC+ 304 benign breast changes)	Plasma No data on collection time	LC–Coularray LC–MS (ng/mL)	**Genistein**: Q1: <9.418 Q2: 9.418–31.761 Q3: 31.761–76.954 Q4: >76.954 **Daidzein**: Q1: <6.718 Q2: 6.718–18.515 Q3: 18.515–42.092 Q4: >42.092	OR (95% CI) | *p* Q4 vs. Q1 **Benign vs. control** Gen: 0.40 (0.23–0.70) | <0.0001 Daid: 0.24 (0.13–0.45) | <0.0001 **Cancer vs. control** Gen: 0.26 (0.13–0.50) | <0.0001 Daid: 0.23 (0.12–0.48) | <0.0001	Nanomolar plasma concentrations of Gen and Daid associated with ↓ BC risk	Low
[247]	144/288	Plasma samples No data about time of collection	LC-Coularray (ng/mL)	Median Genistein: Q1: 31.9 Q2: 108.1 Q3: 190.8 Q4: 353.9 Median Daidzein: Q1: 0 Q2: 12.0 Q3: 27.0 Q4: 53.7	Q1 vs. Q4 Adjusted OR (95% CI) | *p* Gen: 0.34 (0.16–0.74) | 0.02 Daid: 0.71 (0.35–1.44) | 0.54	Nanomolar plasma concentrations of Gen associated with ↓ BC risk No effect of Daid	Moderate
[238]	114/219	Plasma samples No data about time of collection	LC-MS GC-MS (ng/mL)	**Daidzein**: T1: 1.6 (3.3) T2: 2.0 (3.4) T3: 2.5 (3.4) **Genistein**: T1: 5.0 (2.3) T2: 5.5 (2.2) T3: 7.7 (2.4)	OR (95% CI) | *p* Serum Gen: 1.24 (0.98–1.57) Serum Daid: 1.22 (1.01–1.48) Serum Equol: 1.46 (1.05–2.02)	Daid and Equol associated with ↑ BC risk Gen has no effect Serum levels are low	Low
**Western**							
[248]	383/383 87 pre- menopause 296 post-menopause	Plasma No data on collection time	LC-MS-MS (ng/mL)	Isofl Mean (min–max) Daid: 2.6 (0–78) Gen: 3.9 (0.28–57.6) Gly: 0.28 (0–1.38) Tertiles not stated	OR (95% CI) **All women** Gen: 0.68 (0.47–0.98) Daid: 0.83 (0.58–1.19) **Pre–Peri-menopause** Gen: 0.80 (0.38–1.69) Daid: 0.80 (0.34–1.88) **Post-menopause** Gen: 0.69 (0.45–1.04) Daid: 0.88 (0.59–1.32)	Gen tends to ↓ BC risk in the total population but not in subpopulations No effect of Daid No *p*-value	Low
[242]	219/891	Serum No data on collection time	GC-MS HPLC-MS (ng/mL)	Medians (ng/mL) for Control–cases | *p* Genistein: 5.00–4.77 | 0.608 Daidzein: 2.00–1.98 | 0.206 Equol: 0.01–0.01 | 0.005 Glycitein: 0.01–0.01 | <0.0001	OR (95% CI) **All women** Isofl: 1.03 (0.95–1.11) Gen: 1.00 (0.94–1.05) Daid: 1.04 (0.98–1.10) **Pre–peri-menopause** Isofl: 1.30 (1.04–1.64)	No significant link between serum Isofl and BC risk in all women ↑ BC risk in pre- and peri- menopause No *p*-value	Low

## References

[1] WHO (2025). Breast Cancer. https://www.who.int/fr/news-room/fact-sheets/detail/breast-cancer#cms.

[2] Das A., Lavanya K.J., Nandini, Kaur K., Jaitak V. (2023). Effectiveness of Selective Estrogen Receptor Modulators in BC Therapy: An Update. Curr. Med. Chem..

[3] Doucet M., De Berti M., Arbion F., Goupille C., Body G., Ouldamer L. (2025). The impact of the new histological classification of BC with the introduction of HER2 low status. J. Gynecol. Obstet. Hum. Reprod..

[4] Chen Z., Liu Y., Lyu M., Chan C.H., Sun M., Yang X., Qiao S., Chen Z., Yu S., Ren M. (2025). Classifications of triple-negative breast cancer: Insights and current therapeutic approaches. Cell Biosci..

[5] Méndez-Luna D., Martínez-Archundia M., Maroun R.C., Ceballos-Reyes G., Fragoso-Vázquez M.J., González-Juárez D.E., Correa-Basurto J. (2015). Deciphering the GPER/GPR30-agonist and antagonists interactions using molecular modeling studies, molecular dynamics, and docking simulations. J. Biomol. Struct. Dyn..

[6] Mitra S., Dash R. (2018). Natural Products for the Management and Prevention of Breast Cancer. Evid. Based Complement. Altern. Med..

[7] Mierziak J., Kostyn K., Kulma A. (2014). Flavonoids as important molecules of plant interactions with the environment. Molecules.

[8] Bennetau-Pelissero C., Mérillon J.M., Ramawat K. (2018). Plant Proteins from Legumes. Bioactive Molecules in Food.

[9] Gómez J.D., Vital C.E., Oliveira M.G.A., Ramos H.J.O. (2018). Broad range flavonoid profiling by LC/MS of soybean genotypes contrasting for resistance to *Anticarsia gemmatalis* (Lepidoptera: Noctuidae). PLoS ONE.

[10] Canivenc-Lavier M.C., Bennetau-Pelissero C. (2023). Phytoestrogens and Health Effects. Nutrients.

[11] Bennetau-Pelissero C. (2016). Risks and benefits of phytoestrogens: Where are we now?. Curr. Opin. Clin. Nutr. Metab. Care.

[12] Chen S.I., Tseng H.T., Hsieh C.C. (2020). Evaluating the impact of soy compounds on BC using the data mining approach. Food Funct..

[13] Zheng J., Zhu T., Li F., Wu H., Jiang S., Shivappa N., Hébert J.R., Li X., Li Y., Wang H. (2023). Diet Quality and Mortality among Chinese Adults: Findings from the China Health and Nutrition Survey. Nutrients..

[14] Li M.J., Yin Y.C., Wang J., Jiang Y.F. (2014). Green tea compounds in BC prevention and treatment. World J. Clin. Oncol..

[15] Thompson A.S., Tresserra-Rimbau A., Karavasiloglou N., Jennings A., Cantwell M., Hill C., Perez-Cornago A., Bondonno N.P., Murphy N., Rohrmann S. (2023). Association of Healthful Plant-based Diet Adherence with Risk of Mortality and Major Chronic Diseases Among Adults in the UK. JAMA Netw. Open.

[16] Bensaada S., Peruzzi G., Cubizolles L., Denayrolles M., Bennetau-Pelissero C. (2024). Traditional and Domestic Cooking Dramatically Reduce Estrogenic Isoflavones in Soy Foods. Foods.

[17] Otaki N., Kimira M., Katsumata S., Uehara M., Watanabe S., Suzuki K. (2009). Distribution and major sources of flavonoid intakes in the middle-aged Japanese women. J. Clin. Biochem. Nutr..

[18] Hirose K., Imaeda N., Tokudome Y., Goto C., Wakai K., Matsuo K., Ito H., Toyama T., Iwata H., Tokudome S. (2005). Soybean products and reduction of BC risk: A case-control study in Japan. Br. J. Cancer.

[19] Iggo R., MacGrogan G. (2025). Classification of BC Through the Perspective of Cell Identity Models. Adv. Exp. Med. Biol..

[20] Kuiper G.G., Lemmen J.G., Carlsson B., Corton J.C., Safe S.H., van der Saag P.T., van der Burg B., Gustafsson J.A. (1998). Interaction of estrogenic chemicals and phytoestrogens with estrogen receptor beta. Endocrinology.

[21] Arnal J.F., Lenfant F., Metivier R., Flouriot G., Henrion D., Adlanmerini M., Fontaine C., Gourdy P., Chambon P., Katzenellenbogen B. (2017). Membrane and Nuclear Estrogen Receptor Alpha Actions: From Tissue Specificity to Medical Implications. Physiol. Rev..

[22] Chantalat E., Boudou F., Laurell H., Palierne G., Houtman R., Melchers D., Rochaix P., Filleron T., Stella A., Burlet-Schiltz O. (2016). The AF-1-deficient estrogen receptor ERalpha46 isoform is frequently expressed in human breast tumors. BC Res..

[23] Raica M., Jung I., Cîmpean A.M., Suciu C., Mureşan A.M. (2009). From conventional pathologic diagnosis to the molecular classification of breast carcinoma: Are we ready for the change?. Rom. J. Morphol. Embryol..

[24] Rosati R., Oppat K., Huang Y., Kim S., Ratnam M. (2020). Clinical association of progesterone receptor isoform A with BC metastasis consistent with its unique mechanistic role in preclinical models. BMC Cancer.

[25] Liu C., Sun L., Niu N., Hou P., Chen G., Wang H., Zhang Z., Jiang X., Xu Q., Zhao Y. (2025). Molecular classification of hormone receptor-positive /HER2-positive BC reveals potential neoadjuvant therapeutic strategies. Signal Transduct. Target. Ther..

[26] Molina Calistro L., Arancibia Y., Olivera M.A., Domke S., Torres R.F. (2025). Interaction of GPER-1 with the endocrine signaling axis in breast cancer. Front. Endocrinol..

[27] Yu T., Cheng H., Ding Z., Wang Z., Zhou L., Zhao P., Tan S., Xu X., Huang X., Liu M. (2020). GPER mediates decreased chemosensitivity via regulation of ABCG_2_ expression and localization in tamoxifen-resistant BC cells. Mol. Cell Endocrinol..

[28] Seo H., Seo H., Lee S.H., Park Y. (2024). Receptor mediated biological activities of phytoestrogens. Int. J. Biol. Macromol..

[29] Ariyani W., Miyazaki W., Amano I., Hanamura K., Shirao T., Koibuchi N. (2020). Soy Isoflavones Accelerate Glial Cell Migration via GPER-Mediated Signal Transduction Pathway. Front. Endocrinol..

[30] Shinkaruk S., Durand M., Lamothe V., Carpaye A., Martinet A., Chantre P., Vergne S., Nogues X., Moore N., Bennetau-Pelissero C. (2012). Bioavailability of glycitein relatively to other soy Isoflavones in healthy young Caucasian men. Food Chem..

[31] Soukup S.T., Al-Maharik N., Botting N., Kulling S.E. (2014). Quantification of soy Isoflavones and their conjugative metabolites in plasma and urine: An automated and validated UHPLC-MS/MS method for use in large-scale studies. Anal. Bioanal. Chem..

[32] Bolca S., Urpi-Sarda M., Blondeel P., Roche N., Vanhaecke L., Possemiers S., Al-Maharik N., Botting N., De Keukeleire D., Bracke M. (2010). Disposition of soy isoflavones in normal human breast tissue. Am. J. Clin. Nutr..

[33] Ávila-Gálvez M.Á., González-Sarrías A., Martínez-Díaz F., Abellán B., Martínez-Torrano A.J., Fernández-López A.J., Giménez-Bastida J.A., Espín J.C. (2021). Disposition of Dietary Polyphenols in BC Patients’ Tumors, and Their Associated Anticancer Activity: The Particular Case of Curcumin. Mol. Nutr. Food Res..

[34] Pawlicka M.A., Zmorzyński S., Popek-Marciniec S., Filip A.A. (2022). The Effects of Genistein at Different Concentrations on MCF-7 BC Cells and BJ Dermal Fibroblasts. Int. J. Mol. Sci..

[35] Uifălean A., Schneider S., Ionescu C., Lalk M., Iuga C.A. (2015). Soy Isoflavones and BC Cell Lines: Molecular Mechanisms and Future Perspectives. Molecules.

[36] Clark J.W., Santos-Moore A., Stevenson L.E., Frackelton A.R. (1996). Effects of tyrosine kinase inhibitors on the proliferation of human BC cell lines and proteins important in the ras signaling pathway. Int. J. Cancer.

[37] Takamura-Enya T., Ishihara J., Tahara S., Goto S., Totsuka Y., Sugimura T., Wakabayashi K. (2003). Analysis of estrogenic activity of foodstuffs and cigarette smoke condensates using a yeast estrogen screening method. Food Chem. Toxicol..

[38] Bennetau-Pelissero C., Latonnelle K., Lamothe V., Shinkaruk-Poix S., Kaushik S.J. (2004). Screening for oestrogenic activity of plant and food extracts using in vitro trout hepatocyte cultures. Phytochem. Anal..

[39] Bodinet C., Freudenstein J. (2004). Influence of marketed herbal menopause preparations on MCF-7 cell proliferation. Menopause.

[40] Vergne S., Bennetau-Pelissero C., Lamothe V., Chantre P., Potier M., Asselineau J., Perez P., Durand M., Moore N., Sauvant P. (2008). Higher bioavailability of isoflavones after a single ingestion of a soya-based supplement than a soya-based food in young healthy males. Br. J. Nutr..

[41] Pelissero C., Flouriot G., Foucher J.L., Bennetau B., Dunoguès J., Le Gac F., Sumpter J.P. (1993). Vitellogenin synthesis in cultured hepatocytes; an in vitro test for the estrogenic potency of chemicals. J. Steroid Biochem. Mol. Biol..

[42] Bursztyka J., Perdu E., Pettersson K., Pongratz I., Fernández-Cabrera M., Olea N., Debrauwer L., Zalko D., Cravedi J.P. (2008). Biotransformation of genistein and bisphenol A in cell lines used for screening endocrine disruptors. Toxicol. Vitr..

[43] van Die M.D., Bone K.M., Visvanathan K., Kyrø C., Aune D., Ee C., Paller C.J. (2024). Phytonutrients and outcomes following breast cancer: A systematic review and meta-analysis of observational studies. JNCI Cancer Spectr..

[44] Fioravanti L., Cappelletti V., Miodini P., Ronchi E., Brivio M., Di Fronzo G. (1998). Genistein in the control of BC cell growth: Insights into the mechanism of action in vitro. Cancer Lett..

[45] Rahman S.A., Grant L.K., Gooley J.J., Rajaratnam S.M.W., Czeisler C.A., Lockley S.W. (2019). Endogenous Circadian Regulation of Female Reproductive Hormones. J. Clin. Endocrinol. Metab..

[46] Vergne S., Titier K., Bernard V., Asselineau J., Durand M., Lamothe V., Potier M., Perez P., Demotes-Mainard J., Chantre P. (2007). Bioavailability and urinary excretion of isoflavones in humans: Effects of soy-based supplements formulation and equol production. J. Pharm. Biomed. Anal..

[47] Sarkar F.H., Li Y. (2002). Mechanisms of cancer chemoprevention by soy isoflavone genistein. Cancer Metastasis Rev..

[48] Li Z., Li J., Mo B., Hu C., Liu H., Qi H., Wang X., Xu J. (2008). Genistein induces cell apoptosis in MDA-MB-231 BC cells via the mitogen-activated protein kinase pathway. Toxicol. Vitr..

[49] Xie Q., Bai Q., Zou L.Y., Zhang Q.Y., Zhou Y., Chang H., Yi L., Zhu J.D., Mi M.T. (2014). Genistein inhibits DNA methylation and increases expression of tumor suppressor genes in human BC cells. Genes Chromosomes Cancer.

[50] Onoda A., Ueno T., Uchiyama S., Hayashi S., Kato K., Wake N. (2011). Effects of S-equol and natural S-equol supplement (SE5-OH) on the growth of MCF-7 in vitro and as tumors implanted into ovariectomized athymic mice. Food Chem. Toxicol..

[51] Maggiolini M., Vivacqua A., Fasanella G., Recchia A.G., Sisci D., Pezzi V., Montanaro D., Musti A.M., Picard D., Andò S. (2004). The G protein-coupled receptor GPR30 mediates c-fos up-regulation by 17beta-estradiol and phytoestrogens in BC cells. J. Biol. Chem..

[52] Schmitt E., Dekant W., Stopper H. (2001). Assaying the estrogenicity of phytoestrogens in cells of different estrogen sensitive tissues. Toxicol. Vitr..

[53] Choi E.J., Kim G.H. (2013). Antiproliferative activity of daidzein and genistein may be related to ERalpha/c-erbB-2 expression in human BC cells. Mol. Med. Rep..

[54] Dampier K., Hudson E.A., Howells L.M., Manson M.M., Walker R.A., Gescher A. (2001). Differences between human breast cell lines in susceptibility towards growth inhibition by genistein. Br. J. Cancer.

[55] Tonetti D.A., Zhang Y., Zhao H., Lim S.B., Constantinou A.I. (2007). The effect of the phytoestrogens genistein, daidzein, and equol on the growth of tamoxifen-resistant T47D/PKC alpha. Nutr. Cancer.

[56] Yuan B., Wang L., Jin Y., Zhen H., Xu P., Xu Y., Li C., Xu H. (2012). Role of metabolism in the effects of genistein and its phase II conjugates on the growth of human breast cell lines. AAPS J..

[57] Xing J., Chen X., Zhong D. (2005). Absorption and enterohepatic circulation of baicalin in rats. Life Sci..

[58] Gu L., House S.E., Prior R.L., Fang N., Ronis M.J., Clarkson T.B., Wilson M.E., Badger T.M. (2006). Metabolic phenotype of isoflavones differ among female rats, pigs, monkeys, and women. J. Nutr..

[59] Soukup S.T., Helppi J., Müller D.R., Zierau O., Watzl B., Vollmer G., Diel P., Bub A., Kulling S.E. (2016). Phase II metabolism of the soy Isoflavones genistein and daidzein in humans, rats and mice: A cross-species and sex comparison. Arch. Toxicol..

[60] National Toxicology Program (2008). Toxicology and carcinogenesis studies of genistein (Cas No. 446-72-0) in Sprague-Dawley rats (feed study). Natl. Toxicol. Program Tech. Rep. Ser..

[61] National Toxicology Program (2008). Multigenerational reproductive study of genistein (Cas No. 446-72-0) in Sprague-Dawley rats (feed study). Natl. Toxicol. Program Tech. Rep. Ser..

[62] Fritz W.A., Coward L., Wang J., Lamartiniere C.A. (1998). Dietary genistein: Perinatal mammary cancer prevention, bioavailability and toxicity testing in the rat. Carcinogenesis.

[63] Appelt L.C., Reicks M.M. (1999). Soy induces phase II enzymes but does not inhibit dimethylbenz[a]anthracene-induced carcinogenesis in female rats. J. Nutr..

[64] Constantinou A.I., Lantvit D., Hawthorne M., Xu X., van Breemen R.B., Pezzuto J.M. (2001). Chemopreventive effects of soy protein and purified soy isoflavones on DMBA-induced mammary tumors in female Sprague-Dawley rats. Nutr. Cancer.

[65] Lamartiniere C.A., Cotroneo M.S., Fritz W.A., Wang J., Mentor-Marcel R., Elgavish A. (2002). Genistein chemoprevention: Timing and mechanisms of action in murine mammary and prostate. J. Nutr..

[66] Pugalendhi P., Manoharan S. (2010). Chemopreventive potential of genistein and daidzein in combination during 7,12-dimethylbenz[a]anthracene (DMBA) induced mammary carcinogenesis in Sprague-Dawley rats. Pak. J. Biol. Sci..

[67] Brown N.M., Belles C.A., Lindley S.L., Zimmer-Nechemias L., Witte D.P., Kim M.O., Setchell K.D. (2010). Mammary gland differentiation by early life exposure to enantiomers of the soy isoflavone metabolite equol. Food Chem. Toxicol..

[68] Nielsen T.S., Purup S., Wärri A., Godschalk R.W., Hilakivi-Clarke L. (2011). Effects of maternal exposure to cow’s milk high or low in isoflavones on carcinogen-induced mammary tumorigenesis among rat offspring. Cancer Prev. Res..

[69] Hakkak R., Shaaf S., Jo C.H., Macleod S., Korourian S. (2011). Effects of high-isoflavone soy diet vs. casein protein diet and obesity on DMBA-induced mammary tumor development. Oncol. Lett..

[70] Sahin K., Tuzcu M., Sahin N., Akdemir F., Ozercan I., Bayraktar S., Kucuk O. (2011). Inhibitory effects of combination of lycopene and genistein on 7,12- dimethyl benz(a)anthracene-induced BC in rats. Nutr. Cancer.

[71] Kakehashi A., Tago Y., Yoshida M., Sokuza Y., Wei M., Fukushima S., Wanibuchi H. (2012). Hormonally active doses of isoflavone aglycones promote mammary and endometrial carcinogenesis and alter the molecular tumor environment in Donryu rats. Toxicol. Sci..

[72] Phrakonkham P., Brouland J.P., Saad Hel S., Bergès R., Pimpie C., Pocard M., Canivenc-Lavier M.-C., Perrot-Applanat M. (2015). Dietary exposure in utero and during lactation to a mixture of genistein and an anti-androgen fungicide in a rat mammary carcinogenesis model. Reprod. Toxicol..

[73] Zhang X., Cook K.L., Warri A., Cruz I.M., Rosim M., Riskin J., Helferich W., Doerge D., Clarke R., Hilakivi-Clarke L. (2017). Lifetime Genistein Intake Increases the Response of Mammary Tumors to Tamoxifen in Rats. Clin. Cancer Res..

[74] Banys K., Giebultowicz J., Sobczak M., Wyrebiak R., Bielecki W., Wrzesien R., Bobrowska-Korczak B. (2021). Effect of Genistein Supplementation on the Progression of Neoplasms and the Level of the Modified Nucleosides in Rats with Mammary Cancer. In Vivo.

[75] Phrakonkham P., Chevalier J., Desmetz C., Pinnert M.F., Bergès R., Jover E., Davicco M.J., Bennetau-Pelissero C., Coxam V., Artur Y. (2007). Isoflavonoid-based bone-sparing treatments exert a low activity on reproductive organs and on hepatic metabolism of estradiol in ovariectomized rats. Toxicol. Appl. Pharmacol..

[76] Chang H.C., Churchwell M.I., Delclos K.B., Newbold R.R., Doerge D.R. (2000). Mass spectrometric determination of Genistein tissue distribution in diet-exposed Sprague-Dawley rats. J. Nutr..

[77] Murray S.A., Yang S., Demicco E., Ying H., Sherr D.H., Hafer L.J., Rogers A.E., Sonenshein G.E., Xiao Z.X. (2005). Increased expression of MDM2, cyclin D1, and p27Kip1 in carcinogen-induced rat mammary tumors. J. Cell Biochem..

[78] Li M., Zhang Z., Hill D.L., Chen X., Wang H., Zhang R. (2005). Genistein, a dietary isoflavone, down-regulates the MDM2 oncogene at both transcriptional and posttranslational levels. Cancer Res..

[79] Ueda M., Niho N., Imai T., Shibutani M., Mitsumori K., Matsui T., Hirose M. (2003). Lack of significant effects of genistein on the progression of 7,12-dimethylbenz(a)anthracene-induced mammary tumors in ovariectomized Sprague-Dawley rats. Nutr. Cancer.

[80] Allred C.D., Allred K.F., Ju Y.H., Clausen L.M., Doerge D.R., Schantz S.L., Korol D.L., Wallig M.A., Helferich W.G. (2004). Dietary genistein results in larger MNU-induced, estrogen-dependent mammary tumors following ovariectomy of Sprague-Dawley rats. Carcinogenesis.

[81] Qin L.Q., Xu J.Y., Tezuka H., Wang P.Y., Hoshi K. (2007). Commercial soy milk enhances the development of 7,12-dimethylbenz(a)anthracene-induced mammary tumors in rats. In Vivo.

[82] Liu X., Suzuki N., Santosh Laxmi Y.R., Okamoto Y., Shibutani S. (2012). Anti-BC potential of daidzein in rodents. Life Sci..

[83] Ma D., Zhang Y., Yang T., Xue Y., Wang P. (2014). Isoflavone intake inhibits the development of 7,12-dimethylbenz(a)anthracene(DMBA)-induced mammary tumors in normal and ovariectomized rats. J. Clin. Biochem. Nutr..

[84] Yanaka K., Takebayashi J., Matsumoto T., Ishimi Y. (2012). Determination of 15 isoflavone isomers in soy foods and supplements by high-performance liquid chromatography. J. Agric. Food Chem..

[85] Gotoh T., Yamada K., Yin H., Ito A., Kataoka T., Dohi K. (1998). Chemoprevention of N-nitroso-N-methylurea-induced rat mammary carcinogenesis by soy foods or biochanin A. Jpn. J. Cancer Res..

[86] Park K., Choi K., Kim H., Kim K., Lee M.H., Lee J.H., Kim Rim J.C. (2009). Isoflavone-deprived soy peptide suppresses mammary tumorigenesis by inducing apoptosis. Exp. Mol. Med..

[87] Jadhav R.R., Santucci-Pereira J., Wang Y.V., Liu J., Nguyen T.D., Wang J., Jenkins S., Russo J., Huang T.H., Jin V.X. (2017). DNA Methylation Targets Influenced by Bisphenol A and/or Genistein Are Associated with Survival Outcomes in BC Patients. Genes.

[88] Cotroneo M.S., Wang J., Fritz W.A., Eltoum I.E., Lamartiniere C.A. (2002). Genistein action in the prepubertal mammary gland in a chemoprevention model. Carcinogenesis.

[89] Wang J., Jenkins S., Lamartiniere C.A. (2014). Cell proliferation and apoptosis in rat mammary glands following combinational exposure to bisphenol A and genistein. BMC Cancer.

[90] Bensaada S., Raymond I., Pellegrin I., Viallard J.F., Bennetau-Pelissero C. (2023). Validation of ELISAs for Isoflavones and Enterolactone for Phytoestrogen Intake Assessment in the French Population. Nutrients.

[91] Goh J., Niksirat N., Campbell K.L. (2014). Exercise training and immune crosstalk in BC microenvironment: Exploring the paradigms of exercise-induced immune modulation and exercise-induced myokines. Am. J. Transl. Res..

[92] Hsieh C.Y., Santell R.C., Haslam S.Z., Helferich W.G. (1998). Estrogenic effects of genistein on the growth of estrogen receptor-positive human BC (MCF-7) cells in vitro and in vivo. Cancer Res..

[93] Allred C.D., Allred K.F., Ju Y.H., Virant S.M., Helferich W.G. (2001). Soy Diets Containing Varying Amounts of Genistein Stimulate Growth of Estrogen-Dependent (MCF-7) Tumors in a Dose-Dependent Manner. Cancer Res..

[94] Allred C.D., Ju Y.H., Allred K.F., Chang J., Helferich W.G. (2001). Dietary genistin stimulates growth of estrogen-dependent BC tumors similar to that observed with genistein. Carcinogenesis.

[95] Ju Y.H., Allred C.D., Allred K.F., Karko K.L., Doerge D.R., Helferich W.G. (2001). Physiological concentrations of dietary genistein dose-dependently stimulate growth of estrogen-dependent human BC (MCF-7) tumors implanted in athymic nude mice. J. Nutr..

[96] Ju Y.H., Doerge D.R., Allred K.F., Allred C.D., Helferich W.G. (2002). Dietary genistein negates the inhibitory effect of tamoxifen on growth of estrogen-dependent human BC (MCF-7) cells implanted in athymic mice. Cancer Res..

[97] Allred C.D., Allred K.F., Ju Y.H., Goeppinger T.S., Doerge D.R., Helferich W.G. (2004). Soy processing influences growth of estrogen-dependent BC tumors. Carcinogenesis.

[98] Zhou J.R., Yu L., Mai Z., Blackburn G.L. (2004). Combined inhibition of estrogen-dependent human breast carcinoma by soy and tea bioactive components in mice. Int. J. Cancer.

[99] Ju Y.H., Fultz J., Allred K.F., Doerge D.R., Helferich W.G. (2006). Effects of dietary daidzein and its metabolite, equol, at physiological concentrations on the growth of estrogen-dependent human BC (MCF-7) tumors implanted in ovariectomized athymic mice. Carcinogenesis.

[100] Ju Y.H., Allred K.F., Allred C.D., Helferich W.G. (2006). Genistein stimulates growth of human BC cells in a novel, postmenopausal animal model, with low plasma estradiol concentrations. Carcinogenesis.

[101] Gallo D., Ferlini C., Fabrizi M., Prislei S., Scambia G. (2006). Lack of stimulatory activity of a phytoestrogen-containing soy extract on the growth of BC tumors in mice. Carcinogenesis.

[102] Ju Y.H., Doerge D.R., Woodling K.A., Hartman J.A., Kwak J., Helferich W.G. (2008). Dietary genistein negates the inhibitory effect of letrozole on the growth of aromatase-expressing estrogen-dependent human BC cells (MCF-7Ca) in vivo. Carcinogenesis.

[103] Jiang X., Patterson N.M., Ling Y., Xie J., Helferich W.G., Shapiro D.J. (2008). Low concentrations of the soy phytoestrogen genistein induce proteinase inhibitor 9 and block killing of BC cells by immune cells. Endocrinology.

[104] Du M., Yang X., Hartman J.A., Cooke P.S., Doerge D.R., Ju Y.H., Helferich W.G. (2012). Low-dose dietary genistein negates the therapeutic effect of tamoxifen in athymic nude mice. Carcinogenesis.

[105] Andrade J.E., Ju Y.H., Baker C., Doerge D.R., Helferich W.G. (2015). Long-term exposure to dietary sources of genistein induces estrogen-independence in the human BC (MCF-7) xenograft model. Mol. Nutr. Food Res..

[106] Jiang H., Fan J., Cheng L., Hu P., Liu R. (2018). The anticancer activity of genistein is increased in estrogen receptor beta 1-positive BC cells. OncoTargets Ther..

[107] Song H., Hughes J.R., Turner R.T., Iwaniec U.T., Doerge D.R., Helferich W.G. (2020). (±)-Equol does not interact with genistein on estrogen-dependent breast tumor growth. Food Chem. Toxicol..

[108] Allred C.D., Twaddle N.C., Allred K.F., Goeppinger T.S., Churchwell M.I., Ju Y.H., Helferich W.G., Doerge D.R. (2005). Soy processing affects metabolism and disposition of dietary isoflavones in ovariectomized BALB/c mice. J. Agric. Food Chem..

[109] Santell R.C., Kieu N., Helferich W.G. (2000). Genistein inhibits growth of estrogen-independent human BC cells in culture but not in athymic mice. J. Nutr..

[110] Kim H.A., Jeong K.S., Kim Y.K. (2008). Soy extract is more potent than genistein on tumor growth inhibition. Anticancer Res..

[111] Li Y., Meeran S.M., Patel S.N., Chen H., Hardy T.M., Tollefsbol T.O. (2013). Epigenetic reactivation of estrogen receptor-α (ERα) by genistein enhances hormonal therapy sensitivity in ERα-negative breast cancer. Mol. Cancer.

[112] Wei W., Chen Z.J., Zhang K.S., Yang X.L., Wu Y.M., Chen X.H., Huang H.B., Liu H.L., Cai S.H., Du J. (2014). The activation of G protein-coupled receptor 30 (GPR30) inhibits proliferation of estrogen receptor-negative BC cells in vitro and in vivo. Cell Death Dis..

[113] Ford J.A., Clark S.G., Walters E.M., Wheeler M.B., Hurley W.L. (2006). Estrogenic effects of genistein on reproductive tissues of ovariectomized gilts. J. Anim. Sci..

[114] Farmer C., Robertson P., Gilani G.S. (2013). Effects of dose and route of administration of genistein on isoflavone concentrations in post-weaned and gestating sows. Animal.

[115] Farmer C., Palin M.F., Gilani G.S., Weiler H., Vignola M., Choudhary R.K., Capuco A.V. (2010). Dietary genistein stimulates mammary hyperplasia in gilts. Animal.

[116] Mercer K.E., Bhattacharyya S., Sharma N., Chaudhury M., Lin H., Yeruva L., Ronis M.J. (2020). Infant Formula Feeding Changes the Proliferative Status in Piglet Neonatal Mammary Glands Independently of Estrogen Signaling. J. Nutr..

[117] McCarver G., Bhatia J., Chambers C., Clarke R., Etzel R., Foster W., Hoyer P., Leeder J.S., Peters J.M., Rissman E. (2011). NTP-CERHR expert panel report on the developmental toxicity of soy infant formula. Birth Defects Res. B Dev. Reprod. Toxicol..

[118] Wood C.E., Appt S.E., Clarkson T.B., Franke A.A., Lees C.J., Doerge D.R., Cline J.M. (2006). Effects of high-dose soy isoflavones and equol on reproductive tissues in female cynomolgus monkeys. Biol. Reprod..

[119] Wood C.E., Kaplan J.R., Stute P., Cline J.M. (2006). Effects of soy on the mammary glands of premenopausal female monkeys. Fertil. Steril..

[120] Foth D., Cline J.M. (1998). Effects of mammalian and plant estrogens on mammary glands and uteri of macaques. Am. J. Clin. Nutr..

[121] Wood C.E., Hester J.M., Appt S.E., Geisinger K.R., Cline J.M. (2008). Estrogen effects on epithelial proliferation and benign proliferative lesions in the postmenopausal primate mammary gland. Lab. Investig..

[122] Schwen R.J., Nguyen L., Plomley J.B., Jackson R.L. (2012). Toxicokinetics and lack of uterotropic effect of orally administered S-equol. Food Chem. Toxicol..

[123] Möller F.J., Pemp D., Soukup S.T., Wende K., Zhang X., Zierau O., Muders M.H., Bosland M.C., Kulling S.E., Lehmann L. (2016). Soy isoflavone exposure through all life stages accelerates 17β-estradiol-induced mammary tumor onset and growth, yet reduces tumor burden, in ACI rats. Arch. Toxicol..

[124] Santen R.J., Feingold K.R., Ahmed S.F., Anawalt B., Blackman M.R., Boyce A., Chrousos G., Corpas E., de Herder W.W., Dhatariya K., Dungan K. (2000). Benign Breast Disease in Women. Endotext [Internet].

[125] Mylonas I., Jeschke U., Shabani N., Kuhn C., Kunze S., Dian D., Friedl C., Kupka M.S., Friese K. (2007). Steroid receptors ERalpha, ERbeta, PR-A and PR-B are differentially expressed in normal and atrophic human endometrium. Histol. Histopathol..

[126] Yang X.R., Figueroa J.D., Hewitt S.M., Falk R.T., Pfeiffer R.M., Lissowska J., Peplonska B., Brinton L.A., Garcia-Closas M., Sherman M.E. (2013). Estrogen receptor and progesterone receptor expression in normal terminal duct lobular units surrounding invasive breast cancer. BC Res. Treat..

[127] Ellmann S., Sticht H., Thiel F., Beckmann M.W., Strick R., Strissel P.L. (2009). Estrogen and progesterone receptors: From molecular structures to clinical targets. Cell Mol. Life Sci..

[128] Taneja V. (2021). Chapter Fourteen—Sexual dimorphism, aging and immunity. Vitam. Horm..

[129] Flores V.A., Pal L., Manson J.E. (2021). Hormone Therapy in Menopause: Concepts, Controversies, and Approach to Treatment. Endocr. Rev..

[130] Benz C.C. (2008). Impact of aging on the biology of breast cancer. Crit. Rev. Oncol. Hematol..

[131] Gardini E.S., Fiacco S., Mernone L., Ehlert U. (2020). Sleep and Methylation of Estrogen Receptor Genes, ESR1 and GPER, in Healthy Middle-Aged and Older Women: Findings from the Women 40+ Healthy Aging Study. Nat. Sci. Sleep.

[132] Petrakis N.L., Barnes S., King E.B., Lowenstein J., Wiencke J., Lee M.M., Miike R., Kirk M., Coward L. (1996). Stimulatory influence of soy protein isolate on breast secretion in pre- and postmenopausal women. Cancer Epidemiol. Biomark. Prev..

[133] McMichael-Phillips D.F., Harding C., Morton M., Roberts S.A., Howell A., Potten C.S., Bundred N.J. (1998). Effects of soy-protein supplementation on epithelial proliferation in the histologically normal human breast. Am. J. Clin. Nutr..

[134] Hargreaves D.F., Potten C.S., Harding C., Shaw L.E., Morton M.S., Roberts S.A., Howell A., Bundred N.J. (1999). Two-week dietary soy supplementation has an estrogenic effect on normal premenopausal breast. J. Clin. Endocrinol. Metab..

[135] Lu L.W., Chen N.W., Brunder D.G., Nayeem F., Nagamani M., Nishino T.K., Anderson K.E., Khamapirad T. (2022). Soy isoflavones decrease fibroglandular breast tissue measured by magnetic resonance imaging in premenopausal women: A 2-year randomized double-blind placebo controlled clinical trial. Clin. Nutr. ESPEN.

[136] Maskarinec G., Williams A.E., Carlin L. (2003). Mammographic densities in a one-year isoflavone intervention. Eur. J. Cancer Prev..

[137] Maskarinec G., Takata Y., Franke A.A., Williams A.E., Murphy S.P. (2004). A 2-year soy intervention in premenopausal women does not change mammographic densities. J. Nutr..

[138] Finkeldey L., Schmitz E., Ellinger S. (2021). Effect of the Intake of Isoflavones on Risk Factors of Breast Cancer-A Systematic Review of Randomized Controlled Intervention Studies. Nutrients.

[139] Efsa Panel (2015). Risk assessment for peri- and post-menopausal women taking food supplements containing isolated isoflavones. EFSA J..

[140] Colacurci N., De Franciscis P., Atlante M., Mancino P., Monti M., Volpini G., Benvenuti C. (2013). Endometrial, breast and liver safety of soy isoflavones plus Lactobacillus sporogenes in post-menopausal women. Gynecol. Endocrinol..

[141] Delmanto A., Nahas-Neto J., Traiman P., Uemura G., Pessoa E.C., Nahas E.A. (2013). Effects of soy isoflavones on mammographic density and breast parenchyma in postmenopausal women: A randomized, double-blind, placebo-controlled clinical trial. Menopause.

[142] Verheus M., van Gils C.H., Kreijkamp-Kaspers S., Kok L., Peeters P.H., Grobbee D.E., van der Schouw Y.T. (2008). Soy protein containing isoflavones and mammographic density in a randomized controlled trial in postmenopausal women. Cancer Epidemiol. Biomark. Prev..

[143] Maskarinec G., Verheus M., Steinberg F.M., Amato P., Cramer M.K., Lewis R.D., Murray M.J., Young R.L., Wong W.W. (2009). Various doses of soy isoflavones do not modify mammographic density in postmenopausal women. J. Nutr..

[144] Marini H., Bitto A., Altavilla D., Burnett B.P., Polito F., Di Stefano V., Minutoli L., Atteritano M., Levy R.M., D’Anna R. (2008). Breast safety and efficacy of genistein aglycone for postmenopausal bone loss: A follow-up study. J. Clin. Endocrinol. Metab..

[145] Morabito N., Crisafulli A., Vergara C., Gaudio A., Lasco A., Frisina N., D’Anna R., Corrado F., Pizzoleo M.A., Cincotta M. (2002). Effects of genistein and hormone-replacement therapy on bone loss in early postmenopausal women: A randomized double-blind placebo-controlled study. J. Bone Min. Res..

[146] Atkinson C., Warren R.M., Sala E., Dowsett M., Dunning A.M., Healey C.S., Runswick S., Day N.E., Bingham S.A. (2004). Breast Red-clover-derived isoflavones and mammographic breast density: A double-blind, randomized, placebo-controlled trial [ISRCTN42940165]. Cancer Res..

[147] Powles T.J., Howell A., Evans D.G., McCloskey E.V., Ashley S., Greenhalgh R., Affen J., Flook L.A., Tidy A. (2008). Red clover isoflavones are safe and well tolerated in women with a family history of breast cancer. Menopause Int..

[148] Khan S.A., Chatterton R.T., Michel N., Bryk M., Lee O., Ivancic D., Heinz R., Zalles C.M., Helenowski I.B., Jovanovic B.D. (2012). Soy isoflavone supplementation for BC risk reduction: A randomized phase II trial. Cancer Prev. Res..

[149] Cheng G., Wilczek B., Warner M., Gustafsson J.A., Landgren B.M. (2007). Isoflavone treatment for acute menopausal symptoms. Menopause.

[150] Setchell K.D., Brown N.M., Desai P., Zimmer-Nechemias L., Wolfe B.E., Brashear W.T., Kirschner A.S., Cassidy A., Heubi J.E. (2001). Bioavailability of pure isoflavones in healthy humans and analysis of commercial soy isoflavone supplements. J. Nutr..

[151] Shike M., Doane A.S., Russo L., Cabal R., Reis-Filho J.S., Gerald W., Cody H., Khanin R., Bromberg J., Norton L. (2014). The effects of soy supplementation on gene expression in breast cancer: A randomized placebo-controlled study. J. Natl. Cancer Inst..

[152] Brisken C., Scabia V. (2020). 90 Years of Progesterone: Progesterone receptor signaling in the normal breast and its implications for cancer. J. Mol. Endocrinol..

[153] Russo J., Moral R., Balogh G.A., Mailo D., Russo I.H. (2005). The protective role of pregnancy in breast cancer. BC Res..

[154] Colditz G.A., Rosner B.A., Chen W.Y., Holmes M.D., Hankinson S.E. (2004). Risk factors for BC according to estrogen and progesterone receptor status. J. Natl. Cancer Inst..

[155] Gompel A. (2019). Hormone and breast cancer. Presse Med..

[156] Soto A.M., Sonnenschein C. (2015). Endocrine disruptors: DDT, endocrine disruption and breast cancer. Nat. Rev. Endocrinol..

[157] Hardt L., Mahamat-Saleh Y., Aune D., Schlesinger S. (2022). Plant-Based Diets and Cancer Prognosis: A Review of Recent Research. Curr. Nutr. Rep..

[158] Pathak D.R., Stein A.D., He J.P., Noel M.M., Hembroff L., Nelson D.A., Vigneau F., Shen T., Scott L.J., Charzewska J. (2021). Cabbage and Sauerkraut Consumption in Adolescence and Adulthood and BC Risk among US-Resident Polish Migrant Women. Int. J. Environ. Res. Public Health.

[159] Kim T.L., Jeong G.H., Yang J.W., Lee K.H., Kronbichler A., van der Vliet H.J., Grosso G., Galvano F., Aune D., Kim J.Y. (2020). Tea Consumption and Risk of Cancer: An Umbrella Review and Meta-Analysis of Observational Studies. Adv. Nutr..

[160] González-Palacios Torres C., Barrios-Rodríguez R., Muñoz-Bravo C., Toledo E., Dierssen T., Jiménez-Moleón J.J. (2023). Mediterranean diet and risk of breast cancer: An umbrella review. Clin. Nutr..

[161] Vahid F., Hatami M., Sadeghi M., Ameri F., Faghfoori Z., Davoodi S.H. (2018). The association between the Index of Nutritional Quality (INQ) and BC and the evaluation of nutrient intake of BC patients: A case-control study. Nutrition.

[162] Wajszczyk B., Charzewska J., Godlewski D., Zemła B., Nowakowska E., Kozaczka M., Chilimoniuk M., Pathak D.R. (2021). Consumption of Dairy Products and the Risk of Developing BC in Polish Women. Nutrients.

[163] Jun S., Park H., Kim U.J., Choi E.J., Lee H.A., Park B., Lee S.Y., Jee S.H., Park H. (2023). Cancer risk based on alcohol consumption levels: A comprehensive systematic review and meta-analysis. Epidemiol. Health.

[164] Conti B., Bochaton A., Charreire H., Kitzis-Bonsang H., Desprès C., Baffert S., Ngô C. (2022). Influence of geographic access and socioeconomic characteristics on BC outcomes: A systematic review. PLoS ONE.

[165] Renehan A.G., Tyson M., Egger M., Heller R.F., Zwahlen M. (2008). Body-mass index and incidence of cancer: A systematic review and meta-analysis of prospective observational studies. Lancet.

[166] Ogbenna B.T., He X., Wu A.H., Le Marchand L., Wilkens L.R., Butler J., Dyer T., Cheng I., Dallal C.M. (2025). Healthy Lifestyle Index and BC Risk among Postmenopausal Women: The Multiethnic Cohort Study. Cancer Epidemiol. Biomark. Prev..

[167] Yamamoto S., Sobue T., Sasaki S., Kobayashi M., Arai Y., Uehara M., Adlercreutz H., Watanabe S., Takahashi T., Iitoi Y. (2001). Validity and reproducibility of a self-administered food-frequency questionnaire to assess isoflavone intake in a japanese population in comparison with dietary records and blood and urine isoflavones. J. Nutr..

[168] Shirabe R., Saito E., Sawada N., Ishihara J., Takachi R., Abe S.K., Shimazu T., Yamaji T., Goto A., Iwasaki M. (2021). Fermented and nonfermented soy foods and the risk of BC in a Japanese population-based cohort study. Cancer Med..

[169] Whitton C., Ho J.C.Y., Tay Z., Rebello S.A., Lu Y., Ong C.N., van Dam R.M. (2017). Relative Validity and Reproducibility of a Food Frequency Questionnaire for Assessing Dietary Intakes in a Multi-Ethnic Asian Population Using 24-h Dietary Recalls and Biomarkers. Nutrients.

[170] Ozasa K., Nakao M., Watanabe Y., Hayashi K., Miki T., Mikami K., Mori M., Sakauchi F., Washio M., Ito Y. (2005). Association of serum phytoestrogen concentration and dietary habits in a sample set of the JACC Study. J. Epidemiol..

[171] Thanos J., Cotterchio M., Boucher B.A., Kreiger N., Thompson L.U. (2006). Adolescent dietary phytoestrogen intake and breast cancer risk (Canada). Cancer Causes Control.

[172] Frankenfeld C.L., Patterson R.E., Kalhorn T.F., Skor H.E., Howald W.N., Lampe J.W. (2002). Validation of a soy food frequency questionnaire with plasma concentrations of isoflavones in US adults. J. Am. Diet. Assoc..

[173] Monnier L., Colette C., Schlienger J.-L., Halimi S. (2020). Meta-analyses in clinical research: Strengths and weaknesses. Med. Mal. Metab..

[174] Lee H.P., Gourley L., Duffy S.W., Estéve J., Lee J., Day N.E. (1991). Dietary effects on breast-cancer risk in Singapore. Lancet.

[175] Lee H.P., Gourley L., Duffy S.W., Estève J., Lee J., Day N.E. (1992). Risk factors for BC by age and menopausal status: A case-control study in Singapore. Cancer Causes Control.

[176] Hirose K., Tajima K., Hamajima N., Inoue M., Takezaki T., Kuroishi T., Yoshida M., Tokudome S. (1995). A Large-scale, Hospital-based Case-Control Study of Risk Factors of Breast Cancer According to Menopausal Status. Jpn. J. Cancer Res..

[177] Yuan J.M., Wang Q.S., Ross R.K., Henderson B.E., Yu M.C. (1995). Diet and BC in Shanghai and Tianjin, China. Br. J. Cancer.

[178] Dai Q., Shu X.O., Jin F., Potter J.D., Kushi L.H., Teas J., Gao Y.T., Zheng W. (2001). Population-based case-control study of soyfood intake and BC risk in Shanghai. Br. J. Cancer.

[179] Shu X.O., Jin F., Dai Q., Wen W., Potter J.D., Kushi L.H., Ruan Z., Gao Y.T., Zheng W. (2001). Soyfood intake during adolescence and subsequent risk of BC among Chinese women. Cancer Epidemiol. Biomark. Prev..

[180] Hirose K., Takezaki T., Hamajima N., Miura S., Tajima K. (2003). Dietary factors protective against BC in Japanese premenopausal and postmenopausal women. Int. J. Cancer.

[181] Sanderson M., Shu X.O., Yu H., Dai Q., Malin A.S., Gao Y.T., Zheng W. (2004). Insulin-like growth factor-I, soy protein intake, and BC risk. Nutr. Cancer.

[182] Lee M.M., Chang I.Y., Horng C.F., Chang J.S., Cheng S.H., Huang A. (2005). BC and dietary factors in Taiwanese women. Cancer Causes Control.

[183] Li W., Ray R.M., Lampe J.W., Lin M.-G., Gao D.L., Wu C., Nelson Z.C., Fitzgibbons E.D., Horner N., Hu Y.W. (2005). Dietary and other risk factors in women having fibrocystic breast conditions with and without concurrent breast cancer: A nested case-control study in Shanghai, China. Int. J. Cancer.

[184] Shannon J., Ray R., Wu C., Nelson Z., Gao D.L., Li W., Hu W., Lampe J., Horner N., Satia J. (2005). Food and botanical groupings and risk of breast cancer: A case-control study in Shanghai, China. Cancer Epidemiol. Biomark. Prev..

[185] Do M.H., Lee S.S., Jung P.J., Lee M.H. (2007). Intake of fruits, vegetables, and soy foods in relation to BC risk in Korean women: A case-control study. Nutr. Cancer.

[186] Suzuki T., Matsuo K., Tsunoda N., Hirose K., Hiraki A., Kawase T., Yamashita T., Iwata H., Tanaka H., Tajima K. (2008). Effect of soybean on BC according to receptor status: A case-control study in Japan. Int. J. Cancer.

[187] Kim M.K., Kim J.H., Nam S.J., Ryu S., Kong G. (2008). Dietary intake of soy protein and tofu in association with BC risk based on a case-control study. Nutr. Cancer.

[188] Wu A.H., Yu M.C., Tseng C.C., Pike M.C. (2008). Epidemiology of soy exposures and BC risk. Br. J. Cancer.

[189] Zhang C., Ho S.C., Lin F., Cheng S., Fu J., Chen Y. (2010). Soy product and isoflavone intake and BC risk defined by hormone receptor status. Cancer Sci..

[190] Koh W.P., Van Den Berg D., Jin A., Wang R., Yuan J.-M., Yu M.C. (2011). Combined effects of MDM2 SNP309 and TP53 R72P polymorphisms, and soy isoflavones on BC risk among Chinese women in Singapore. BC Res. Treat..

[191] Toi M., Hirota S., Tomotaki A., Sato N., Hozumi Y., Anan K., Nagashima T., Tokuda Y., Masuda N., Ohsumi S. (2013). Probiotic Beverage with Soy Isoflavone Consumption for BC Prevention: A Case-control Study. Curr. Nutr. Food Sci..

[192] Zhu Y.Y., Zhou L., Jiao S.C., Xu L.Z. (2011). Relationship between soy food intake and BC in China. Asian Pac. J. Cancer Prev..

[193] Chang Y.J., Hou Y.C., Chen L.J., Wu J.H., Wu C.C., Chang Y.J., Chung K.P. (2017). Is vegetarian diet associated with a lower risk of BC in Taiwanese women?. BMC Public Health.

[194] Cao S., Liu L., Zhu Q., Zhu Z., Zhou J., Wei P., Wu M. (2022). Adherence to the vegetable-fruit-soy dietary pattern, a reference from mediterranean diet, protects against postmenopausal BC among Chinese women. Front. Nutr..

[195] Wu A.H., Ziegler R.G., Horn-Ross P.L., Nomura A.M., West D.W., Kolonel L.N., Rosenthal J.F., Hoover R.N., Pike M.C. (1996). Tofu and risk of BC in Asian-Americans. Cancer Epidemiol. Biomark. Prev..

[196] Horn-Ross P.L., John E.M., Lee M., Stewart S.L., Koo J., Sakoda L.C., Shiau A.C., Goldstein J., Davis P., Perez-Stable E.J. (2001). Phytoestrogen consumption and BC risk in a multiethnic population: The Bay Area BC Study. Am. J. Epidemiol..

[197] Wu A.H., Wan P., Hankin J., Tseng C.C., Yu M.C., Pike M.C. (2002). Adolescent and adult soy intake and risk of BC in Asian-Americans. Carcinogenesis.

[198] Wu A.H., Yu M.C., Tseng C.C., Hankin J., Pike M.C. (2003). Green tea and risk of BC in Asian Americans. Int. J. Cancer.

[199] Iwasaki M., Hamada G.S., Nishimoto I.N., Netto M.M., Motola J., Laginha F.M., Kasuga Y., Yokoyama S., Onuma H., Nishimura H. (2009). Dietary isoflavone intake and BC risk in case–control studies in Japanese, Japanese Brazilians, and non-Japanese Brazilians. BC Res. Treat..

[200] Peterson J., Lagiou P., Samoli E., Lagiou A., Katsouyanni K., La Vecchia C., Dwyer J., Trichopoulos D. (2003). Flavonoid intake and BC risk: A case--control study in Greece. Br. J. Cancer.

[201] dos Santos Silva I., Mangtani P., McCormack V., Bhakta D., McMichael A.J., Sevak L. (2004). Phyto-oestrogen intake and BC risk in South Asian women in England: Findings from a population-based case-control study. Cancer Causes Control.

[202] Linseisen J., Piller R., Hermann S., Chang-Claude J., German Case-Control Study (2004). Dietary phytoestrogen intake and premenopausal BC risk in a German case-control study. Int. J. Cancer.

[203] Bohlscheid-Thomas S., Hoting I., Boeing H., Wahrendorf J. (1997). Reproducibility and relative validity of food group intake in a food frequency questionnaire developed for the German part of the EPIC project. European Prospective Investigation into Cancer and Nutrition. Int. J. Epidemiol..

[204] Cotterchio M., Boucher B.A., Kreiger N., Mills C.A., Thompson L.U. (2008). Dietary phytoestrogen intake--lignans and Isofl--and BC risk (Canada). Cancer Causes Control.

[205] Anderson L.N., Cotterchio M., Boucher B.A., Kreiger N. (2013). Phytoestrogen intake from foods, during adolescence and adulthood, and risk of BC by estrogen and progesterone receptor tumor subgroup among Ontario women. Int. J. Cancer.

[206] Qin L.Q., Xu J.Y., Wang P.Y., Hoshi K. (2006). Soyfood intake in the prevention of BC risk in women: A meta-analysis of observational epidemiological studies. J. Nutr. Sci. Vitaminol..

[207] Trock B.J., Hilakivi-Clarke L., Clarke R. (2006). Meta-analysis of soy intake and BC risk. J. Natl. Cancer Inst..

[208] Zhao T.-T., Jin F., Li J.-G., Xu Y.-Y., Dong H.T., Liu Q., Xing P., Zhu G.-L., Xu H., Miao Z.-F. (2019). Dietary isoflavones or isoflavone-rich food intake and BC risk: A meta-analysis of prospective cohort studies. Clin. Nutr..

[209] Shin S., Fu J., Shin W.-K., Huang D., Min S., Kang D. (2023). Association of food groups and dietary pattern with BC risk: A systematic review and meta-analysis. Clin. Nutr..

[210] Ho S.C., Yeo W., Goggins W., Kwok C., Cheng A., Chong M., Lee R., Cheung K.L. (2021). Pre-diagnosis and early post-diagnosis dietary soy isoflavone intake and survival outcomes: A prospective cohort study of early stage BC survivors. Cancer Treat. Res. Commun..

[211] Chan S.G., Murphy P.A., Ho S.C., Kreiger N., Darlington G., So E.K., Chong P.Y. (2009). Isoflavonoid content of Hong Kong soy foods. J. Agric. Food Chem..

[212] Wei Y., Lv J., Guo Y., Bian Z., Gao M., Du H., Yang L., Chen Y., Zhang X., Wang T. (2020). Soy intake and BC risk: A prospective study of 300,000 Chinese women and a dose-response meta-analysis. Eur. J. Epidemiol..

[213] Baglia M.L., Zheng W., Li H., Yang G., Gao J., Gao Y.T., Shu X.O. (2016). The association of soy food consumption with the risk of subtype of breast cancers defined by hormone receptor and HER2 status. Int. J. Cancer.

[214] Wada K., Nakamura K., Tamai Y., Tsuji M., Kawachi T., Hori A., Takeyama N., Tanabashi S., Matsushita S., Tokimitsu N. (2013). Soy isoflavone intake and BC risk in Japan: From the Takayama study. Int. J. Cancer.

[215] Zhang Y.F., Kang H.B., Li B.L., Zhang R.M. (2012). Positive effects of soy isoflavone food on survival of BC patients in China. Asian Pac. J. Cancer Prev..

[216] Butler L.M., Wu A.H., Wang R., Koh W.-P., Yuan J.-M., Yu M.C. (2010). A vegetable-fruit-soy dietary pattern protects against BC among postmenopausal Singapore Chinese women. Am. J. Clin. Nutr..

[217] Shu X.O., Zheng Y., Cai H., Gu K., Chen Z., Zheng W., Lu W. (2009). Soy food intake and BC survival. JAMA.

[218] Lee S.A., Shu X.O., Li H., Yang G., Cai H., Wen W., Ji B.T., Gao J., Gao Y.T., Zheng W. (2009). Adolescent and adult soy food intake and BC risk: Results from the Shanghai Women’s Health Study. Am. J. Clin. Nutr..

[219] Wu A.H., Koh W.P., Wang R., Lee H.P., Yu M.C. (2008). Soy intake and BC risk in Singapore Chinese Health Study. Br. J. Cancer.

[220] Nishio K., Niwa Y., Toyoshima H., Tamakoshi K., Kondo T., Yatsuya H., Yamamoto A., Suzuki S., Tokudome S., Lin Y. (2007). Consumption of soy foods and the risk of breast cancer: Findings from the Japan Collaborative Cohort (JACC) Study. Cancer Causes Control.

[221] Yamamoto S., Sobue T., Kobayashi M., Sasaki S., Tsugane S. (2003). Japan Public Health Center-Based Prospective Study on Cancer Cardiovascular Diseases Group. Soy, isoflavones, and BC risk in Japan. J. Natl. Cancer Inst..

[222] Key T.J., Sharp G.B., Appleby P.N., Beral V., Goodman M.T., Soda M., Mabuchi K. (1999). Soya foods and BC risk: A prospective study in Hiroshima and Nagasaki, Japan. Br. J. Cancer.

[223] Fan Y., Wang M., Li Z., Jiang H., Shi J., Shi X., Liu S., Zhao J., Kong L., Zhang W. (2022). Intake of Soy, Soy Isoflavones and Soy Protein and Risk of Cancer Incidence and Mortality. Front. Nutr..

[224] Conroy S.M., Maskarinec G., Park S.-Y., Wilkens L.R., Henderson B.E., Kolonel L.N. (2013). The effects of soy consumption before diagnosis on BC survival: The Multiethnic Cohort Study. Nutr. Cancer.

[225] Nechuta S.J., Caan B.J., Chen W.Y., Lu W., Chen Z., Kwan M.L., Flatt S.W., Zheng Y., Zheng W., Pierce J.P. (2012). Soy food intake after diagnosis of BC and survival: An in-depth analysis of combined evidence from cohort studies of US and Chinese women. Am. J. Clin. Nutr..

[226] Caan B.J., Natarajan L., Parker B., Gold E.B., Thomson C., Newman V., Rock C.L., Pu M., Al-Delaimy W., Pierce J.P. (2011). Soy food consumption and BC prognosis. Cancer Epidemiol. Biomark. Prev..

[227] Fraser G.E., Jaceldo-Siegl K., Orlich M., Mashchak A., Sirirat R., Knutsen S. (2020). Dairy, soy, and risk of breast cancer: Those confounded milks. Int. J. Epidemiol..

[228] Touillaud M., Gelot A., Mesrine S., Bennetau-Pelissero C., Clavel-Chapelon F., Arveux P., Bonnet F., Gunter M., Boutron-Ruault M.-C., Fournier A. (2019). Use of dietary supplements containing soy isoflavones and BC risk among women aged >50 y: A prospective study. Am. J. Clin. Nutr..

[229] Zhang F.F., Haslam D.E., Terry M.B., Knight J.A., Andrulis I.L., Daly M.B., Buys S.S., John E.M. (2017). Dietary isoflavone intake and all-cause mortality in BC survivors: The BC Family Registry. Cancer.

[230] Morimoto Y., Maskarinec G., Park S.-Y., Ettienne R., Matsuno R.K., Long C., Steffen A.D., Henderson B.E., Kolonel L.N., Marchand L. (2014). Dietary isoflavone intake is not statistically significantly associated with BC risk in the Multiethnic Cohort. Br. J. Nutr..

[231] Guha N., Kwan M.L., Quesenberry C.P., Weltzien E.K., Castillo A.L., Caan B.J. (2009). Soy isoflavones and risk of cancer recurrence in a cohort of BC survivors: The Life After Cancer Epidemiology study. BC Res. Treat..

[232] Kirk P., Patterson R.E., Lampe J. (1999). Development of a soy food frequency questionnaire to estimate isoflavone consumption in US adults. J. Am. Diet. Assoc..

[233] Pillow P.C., Duphorne C.M., Chang S., Contois J.H., Strom S.S., Spitz M.R., Hursting S.D. (1999). Development of a database for assessing dietary phytoestrogen intake. Nutr. Cancer.

[234] Travis R.C., Allen N.E., Appleby P.N., Spencer E.A., Roddam A.W., Key T.J. (2008). A prospective study of vegetarianism and isoflavone intake in relation to BC risk in British women. Int. J. Cancer.

[235] Horn-Ross P.L., Hoggatt K.J., West D.W., Krone M.R., Stewart S.L., Anton H., Bernstei C.L., Deapen D., Peel D., Pinder R. (2002). Recent diet and BC risk: The California Teachers Study (USA). Cancer Causes Control.

[236] Zheng W., Dai Q., Custer L.J., Shu X.O., Wen W.Q., Jin F., Franke A.A. (1999). Urinary excretion of isoflavonoids and the risk of breast cancer. Cancer Epidemiol. Biomark. Prev..

[237] Gamache P.H., Acworth I.N. (1998). Analysis of phytoestrogens and polyphenols in plasma, tissue, and urine using HPLC with coulometric array detection. Proc. Soc. Exp. Biol. Med..

[238] Grace P.B., Taylor J.I., Low Y.L., Luben R.N., Mulligan A.A., Botting N.P., Dowsett M., Welch A.A., Khaw K.T., Wareham N.J. (2004). Phytoestrogen concentrations in serum and spot urine as biomarkers for dietary phytoestrogen intake and their relation to BC risk in European prospective investigation of cancer and nutrition-Norfolk. Cancer Epidemiol. Biomark. Prev..

[239] Dai Q., Franke A.A., Jin F., Shu X.O., Hebert J.R., Custer L.J., Cheng J., Gao Y.T., Zheng W. (2002). Urinary excretion of phytoestrogens and risk of BC among Chinese women in Shanghai. Cancer Epidemiol. Biomark. Prev..

[240] Dai Q., Franke A.A., Yu H., Shu X.O., Jin F., Hebert J.R., Custer L.J., Gao Y.T., Zheng W. (2003). Urinary phytoestrogen excretion and BC risk: Evaluating potential effect modifiers endogenous estrogens and anthropometrics. Cancer Epidemiol. Biomark. Prev..

[241] den Tonkelaar I., Keinan-Boker L., Veer P.V., Arts C.J., Adlercreutz H., Thijssen J.H., Peeters P.H. (2001). Urinary phytoestrogens and postmenopausal BC risk. Cancer Epidemiol. Biomark. Prev..

[242] Ward H., Chapelais G., Kuhnle G.G., Luben R., Khaw K.T., Bingham S. (2008). European Prospective into Cancer-Norfolk cohort. BC risk in relation to urinary and serum biomarkers of phytoestrogen exposure in the European Prospective into Cancer-Norfolk cohort study. BC Res..

[243] Goodman M.T., Shvetsov Y.B., Wilkens L.R., Franke A.A., Le Marchand L., Kakazu K.K., Nomura A.M., Henderson B.E., Kolonel L.N. (2009). Urinary phytoestrogen excretion and postmenopausal BC risk: The multiethnic cohort study. Cancer Prev. Res..

[244] Lee A., Bensaada S., Lamothe V., Lacoste M., Bennetau-Pelissero C. (2022). Endocrine disruptors on and in fruits and vegetables: Estimation of the potential exposure of the French population. Food Chem..

[245] Mathey J., Lamothe V., Coxam V., Potier M., Sauvant P., Bennetau-Pelissero C. (2006). Concentrations of isoflavones in plasma and urine of post-menopausal women chronically ingesting high quantities of soy isoflavones. J. Pharm. Biomed. Anal..

[246] Lampe J.W., Nishino Y., Ray R.M., Wu C., Li W., Lin M.G., Gao D.L., Hu Y., Shannon J., Stalsberg H. (2007). Plasma isoflavones and fibrocystic breast conditions and BC among women in Shanghai, China. Cancer Epidemiol. Biomark. Prev..

[247] Iwasaki M., Inoue M., Otani T., Sasazuki S., Kurahashi N., Miura T., Yamamoto S., Tsugane S., Japan Public Health Center-based prospective study group (2008). Plasma isoflavone level and subsequent risk of BC among Japanese women: A nested case-control study from the Japan Public Health Center-based prospective study group. J. Clin. Oncol..

[248] Verheus M., van Gils C.H., Keinan-Boker L., Grace P.B., Bingham S.A., Peeters P.H. (2007). Plasma phytoestrogens and subsequent BC risk. J. Clin. Oncol..

[249] Yoshikata R., Myint K.Z.Y., Taguchi J. (2024). Comparison of blood and urine concentrations of equol by LC–MS/MS method and factors associated with equol production in 466 Japanese men and women. PLoS ONE.

[250] Uehara M., Arai Y., Watanabe S., Adlercreutz H. (2000). Comparison of plasma and urinary phytoestrogens in Japanese and Finnish women by time-resolved fluoroimmunoassay. Biofactors.

[251] Eustache F., Mondon F., Canivenc-Lavier M.C., Lesaffre C., Fulla Y., Berges R., Cravedi J.P., Vaiman D., Auger J. (2009). Chronic dietary exposure to a low-dose mixture of genistein and vinclozolin modifies the reproductive axis, testis transcriptome, and fertility. Environ. Health Perspect..

[252] Setchell K.D. (1998). Phytoestrogens: The biochemistry, physiology, and implications for human health of soy isoflavones. Am. J. Clin. Nutr..

